# Supplementary material for: A Novel Intraoperative Mapping Device Detects the Thermodynamic Response Function
Source: Brain Sci. 2023 Jul 19;13(7):1091. doi: 10.3390/brainsci13071091 (PMC10377735; doi:10.3390/brainsci13071091)

Supplementary Material

Part 1: Individual Subject PCA Results

Patient 1: Lip Pursing

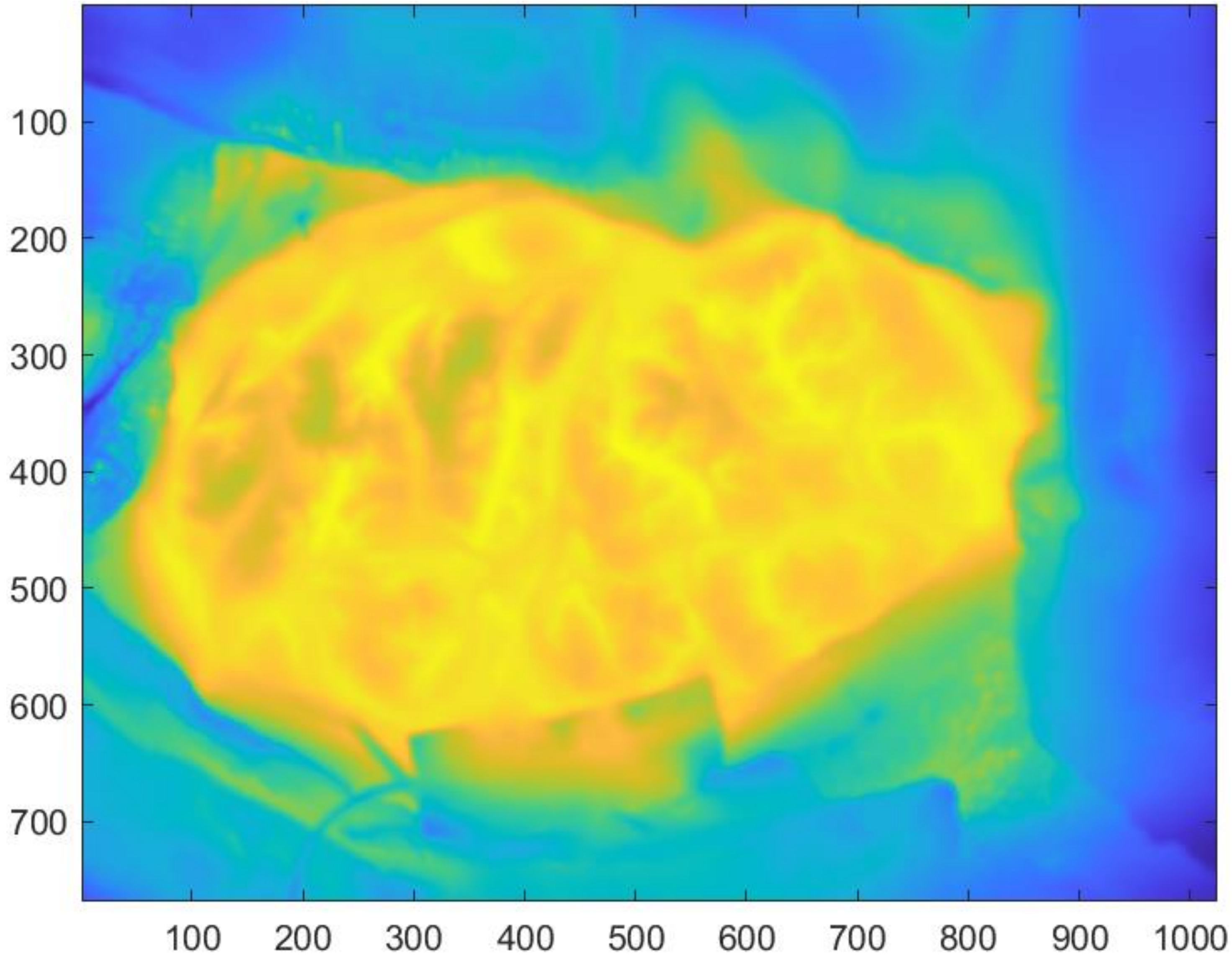
100
200
300
400
500
600
700
100
200
300
400
500
600
700
800
900
1000

P1 (Lip Pursing) DES Key

A. Teeth, Sensory (right)
B. Lips (right) tingling
C. Teeth (right)
D. Teeth, tongue (right)
E. Lips (right)
F. Speech arrest
G. Speech arrest
H. Anomia
I. Anomia

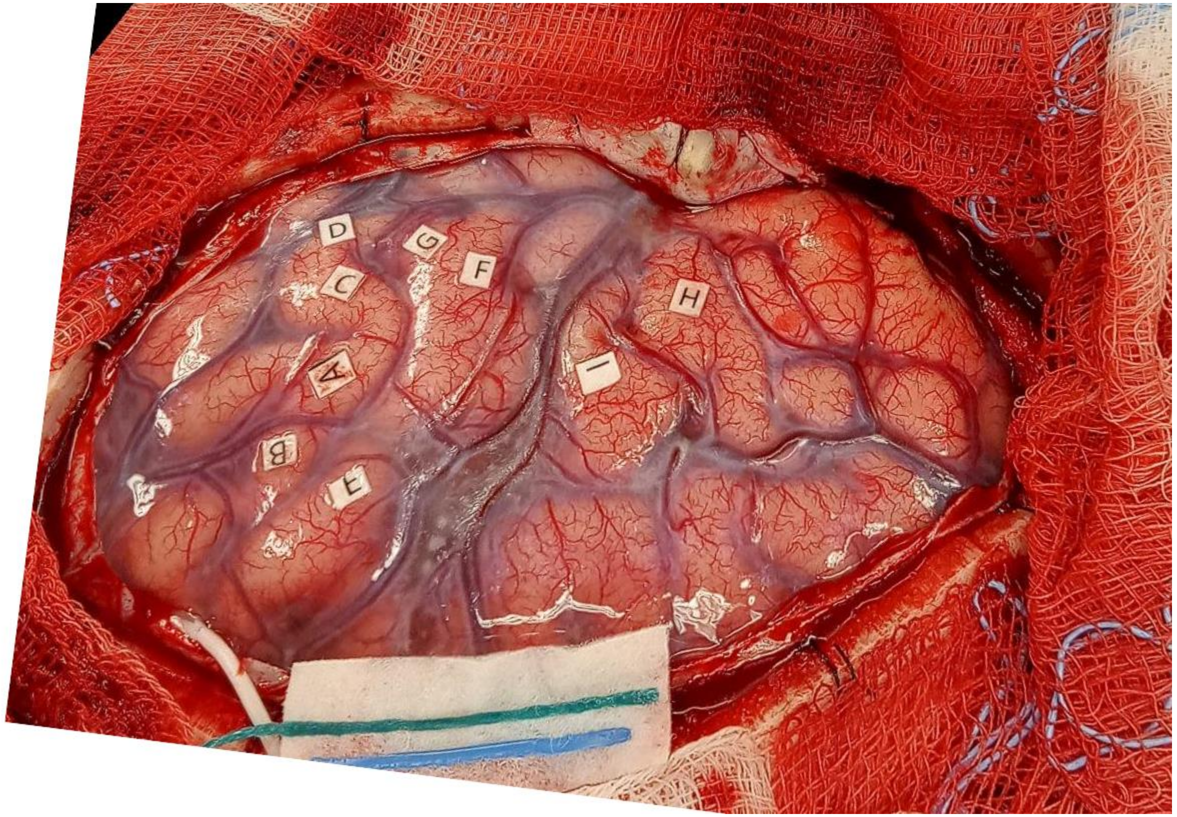

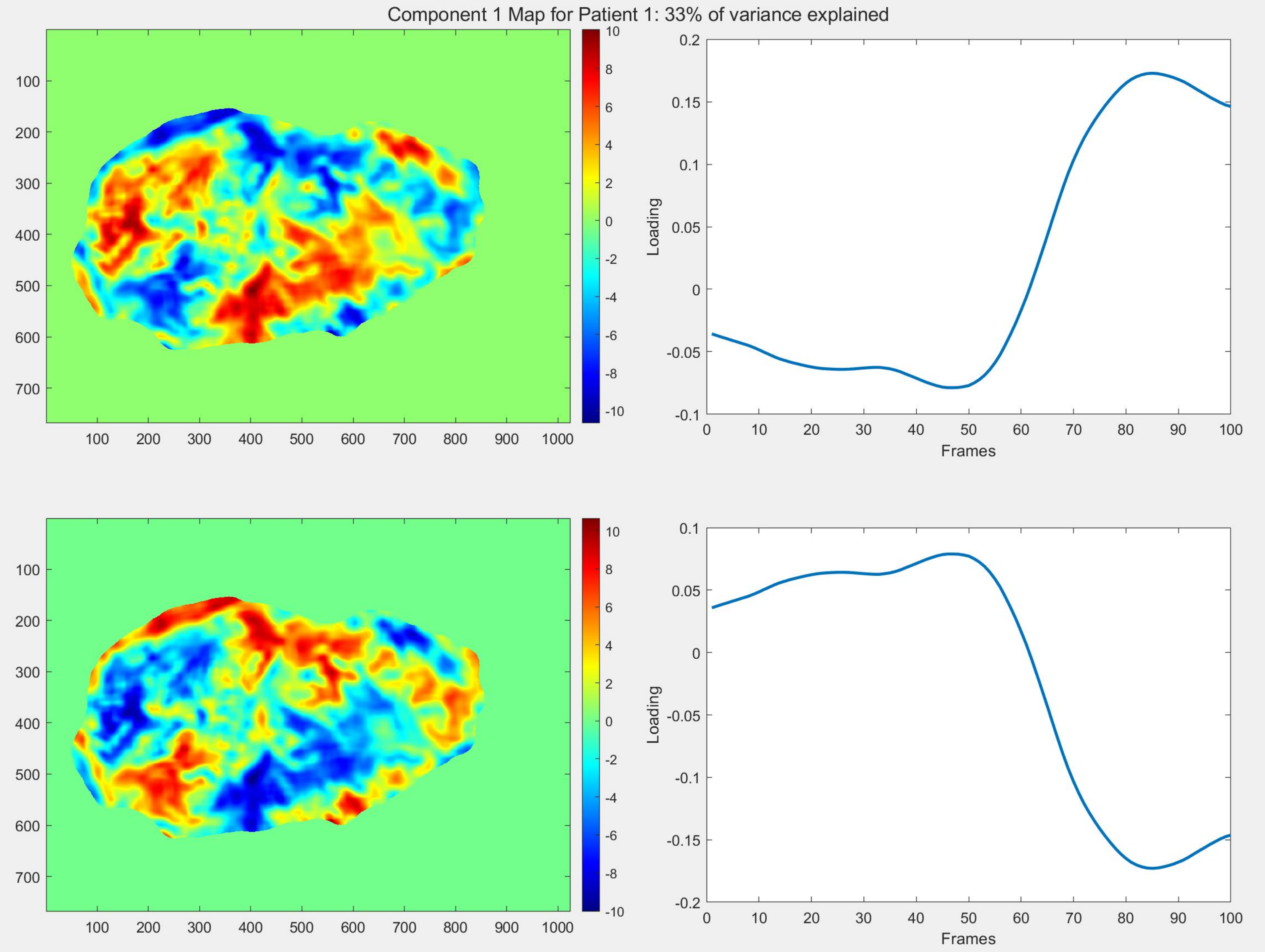

Component 1 Map for Patient 1: 33% of variance explained
Loading
Frames
Loading
Frames

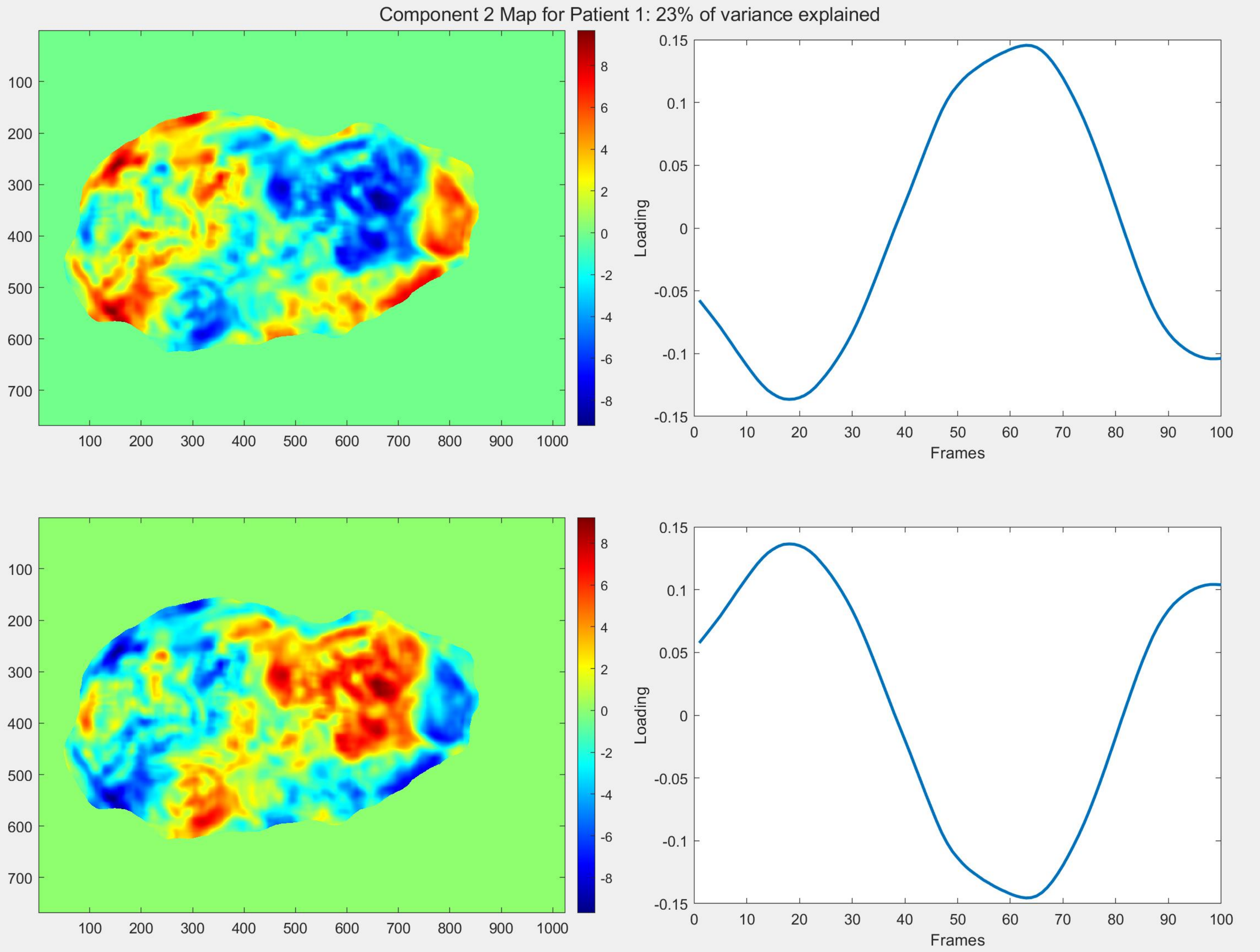

Component 2 Map for Patient 1: 23% of variance explained
Loading
Frames
Loading
Frames

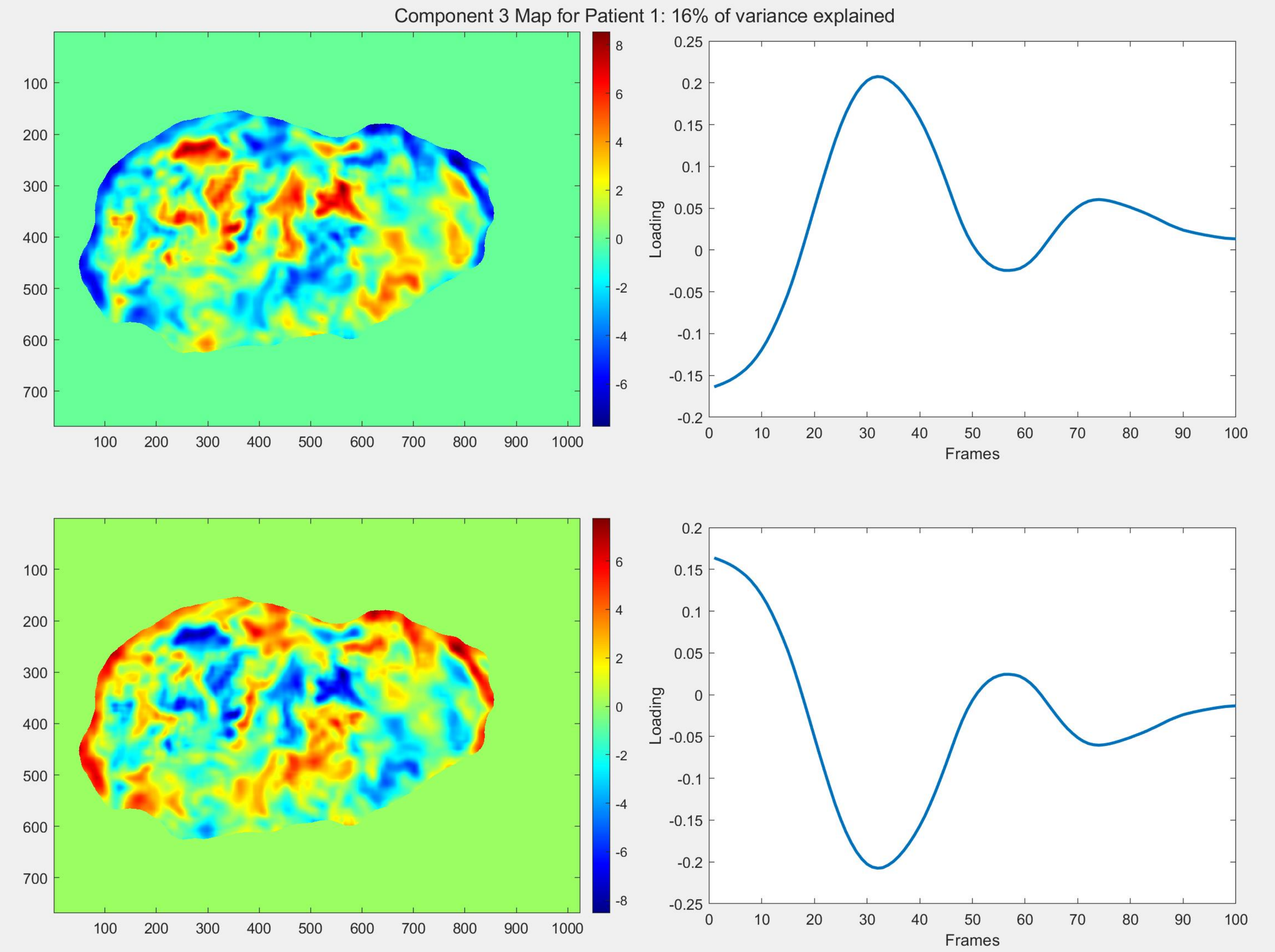
Component 3 Map for Patient 1: 16% of variance explained
Loading
Frames
Loading
Frames

Component 4 Map for Patient 1: 10% of variance explained

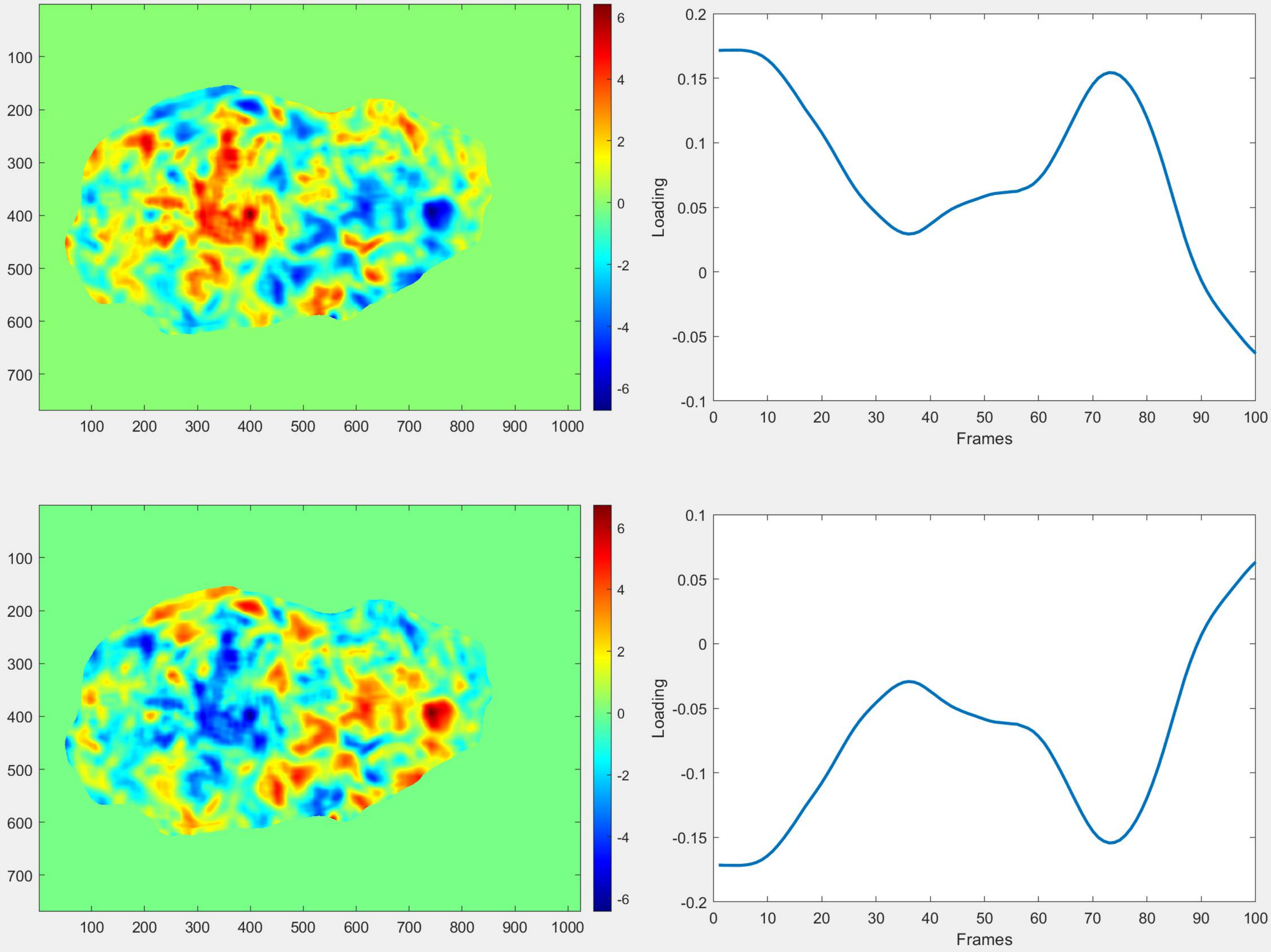

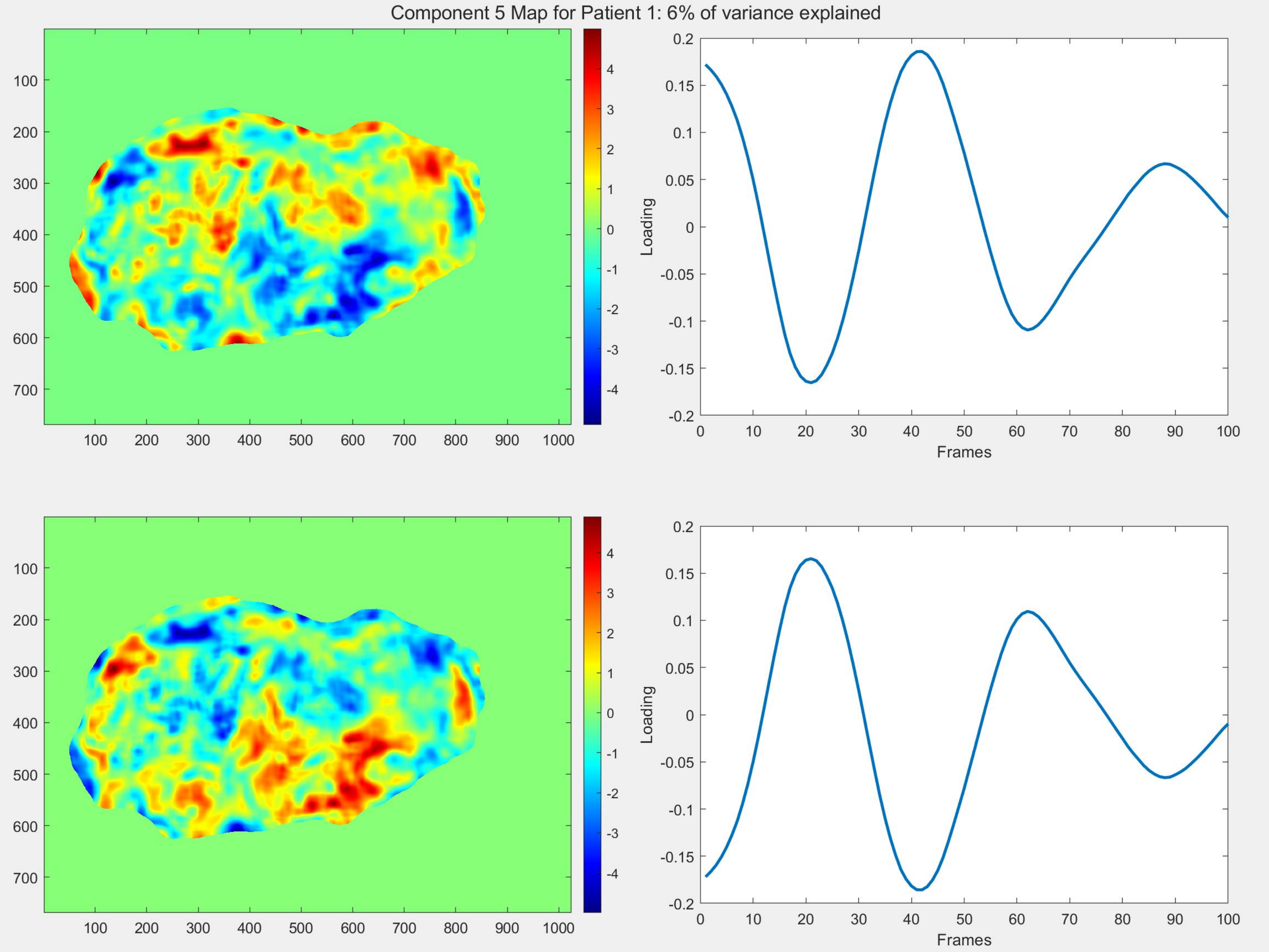

Component 5 Map for Patient 1: 6% of variance explained
Loading
Frames

Patient 2: Lip Pursing

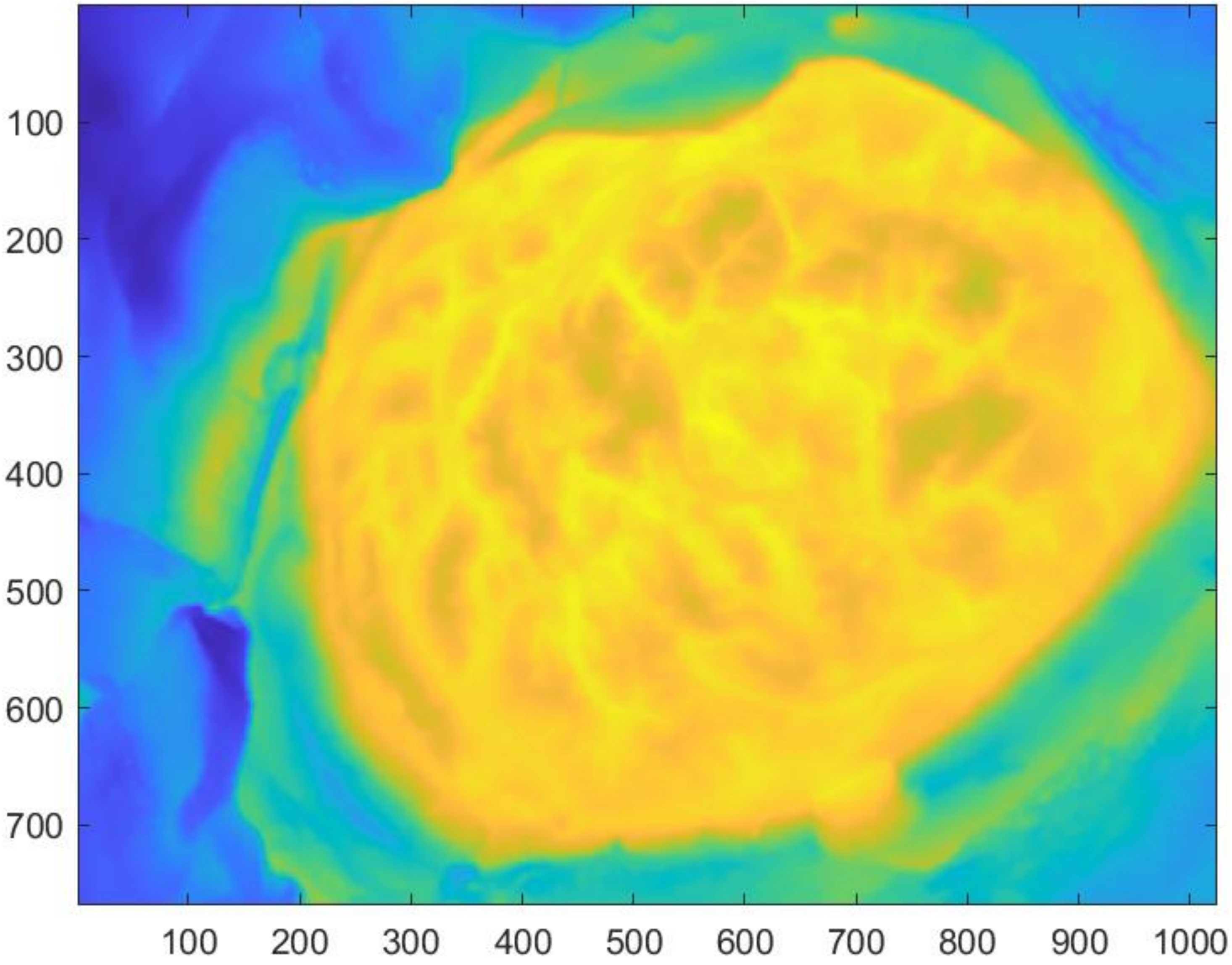
100
200
300
400
500
600
700
100
200
300
400
500
600
700
800
900
1000

P2 (Lip Pursing) DES Key

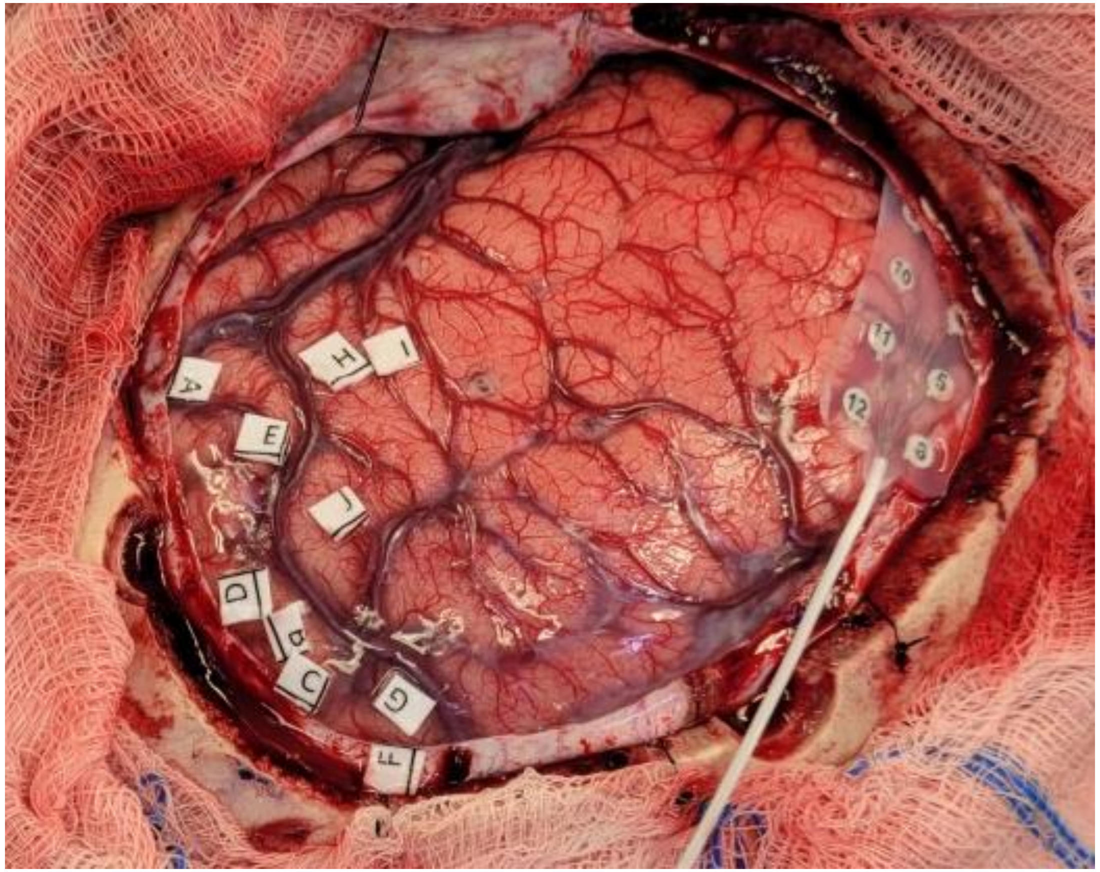

A – Tongue tingling all over
B – Thumb sensory
C – Right hand sensory (3 & 4)
D – Thumb tingling
E – Tongue tingling & movement
F – Wrist flexion
G – Thumb movement
H – Hesitation
I – Hesitation
J – Dysarthria

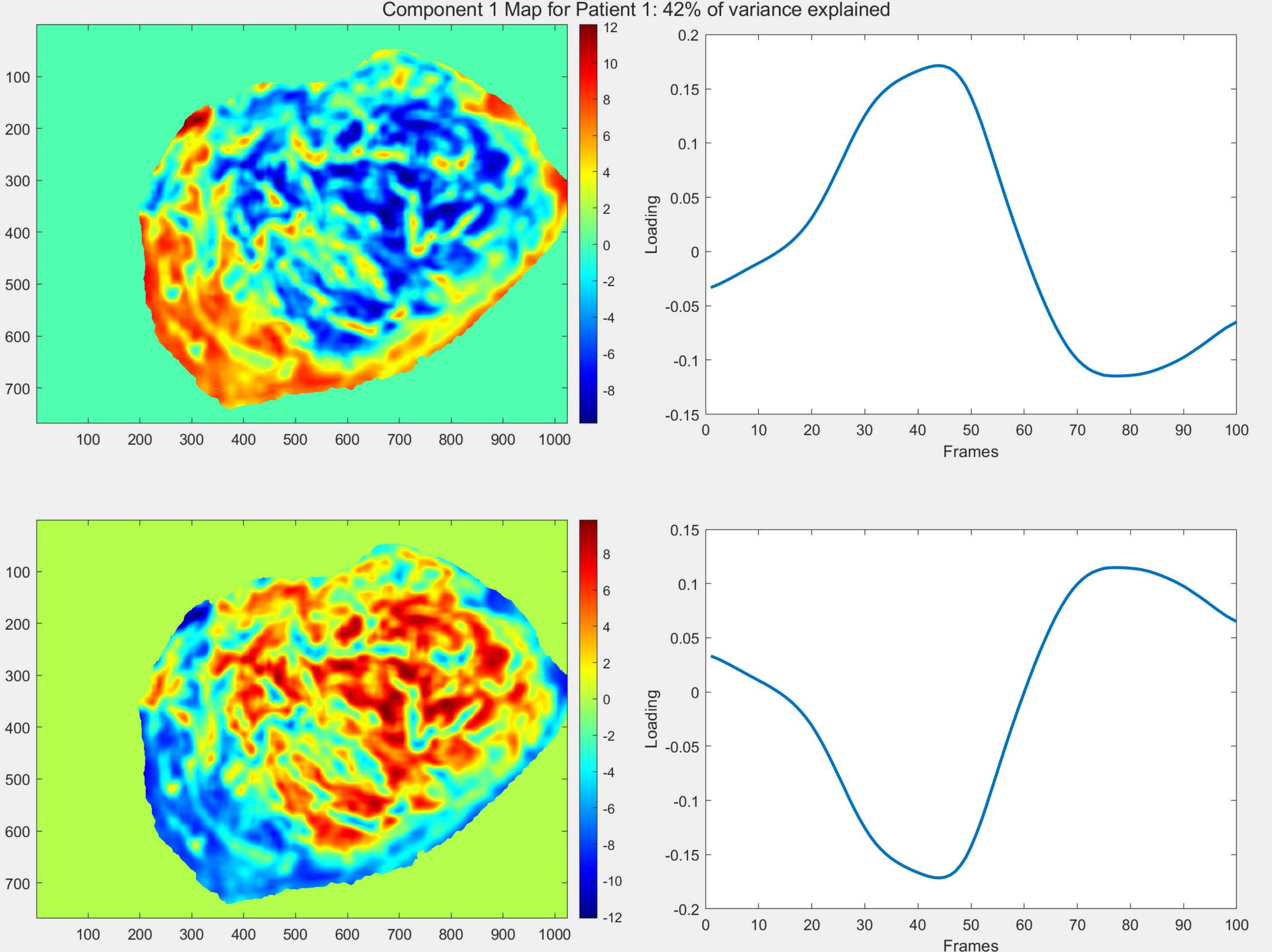
Component 1 Map for Patient 1: 42% of variance explained
Loading
Frames
Loading
Frames

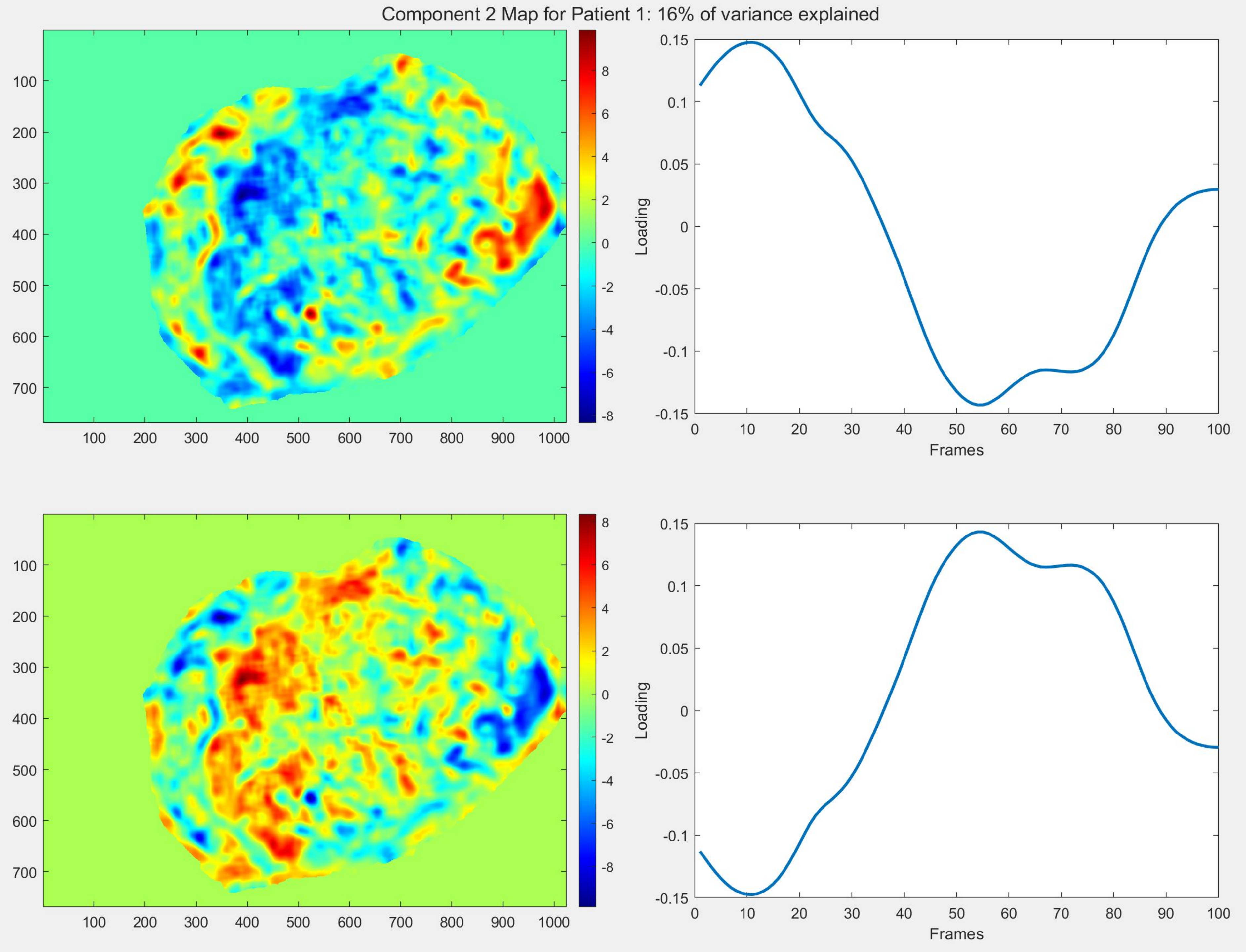
Component 2 Map for Patient 1: 16% of variance explained
Loading
Frames
Loading
Frames

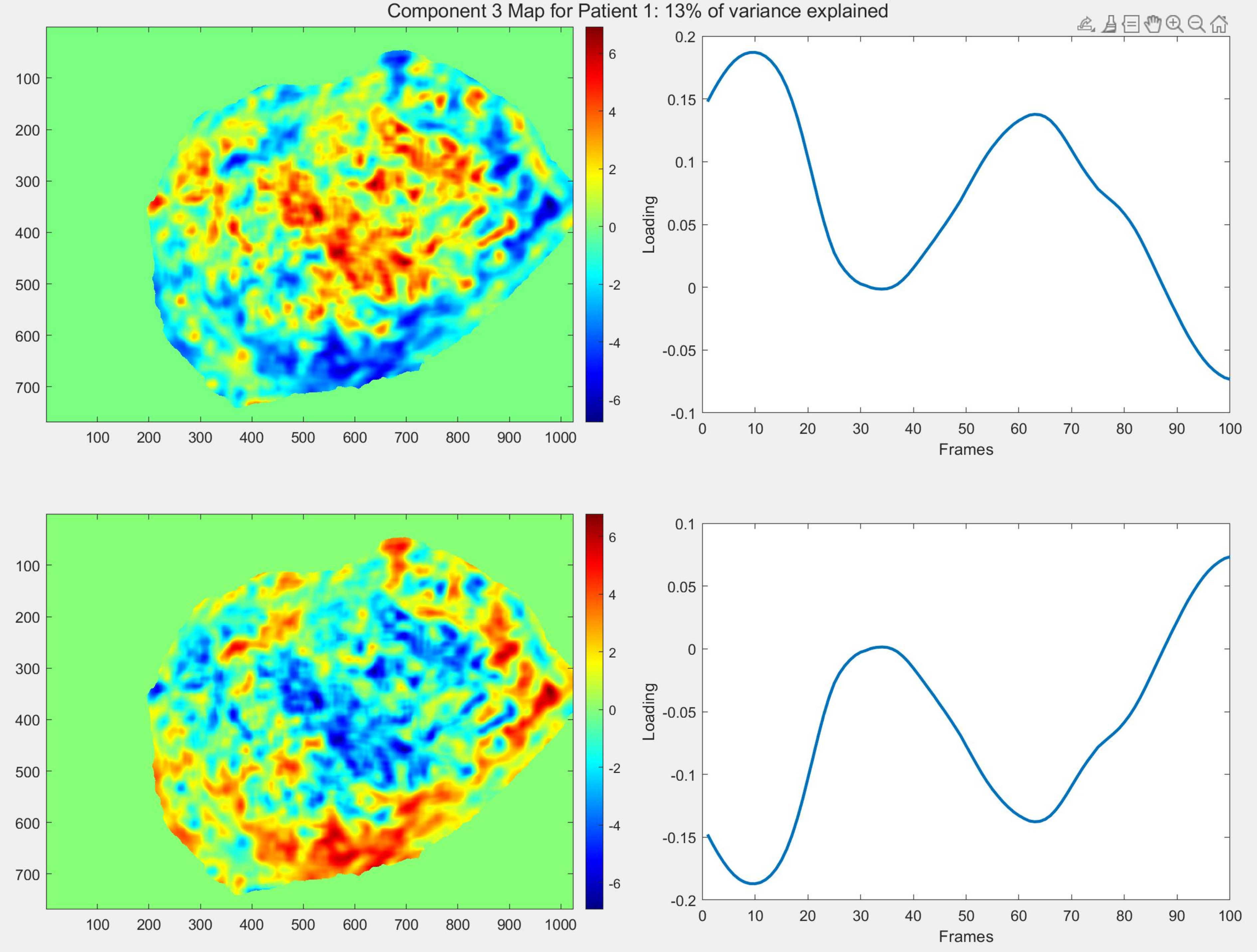
Component 3 Map for Patient 1: 13% of variance explained
Loading
Frames
Loading
Frames

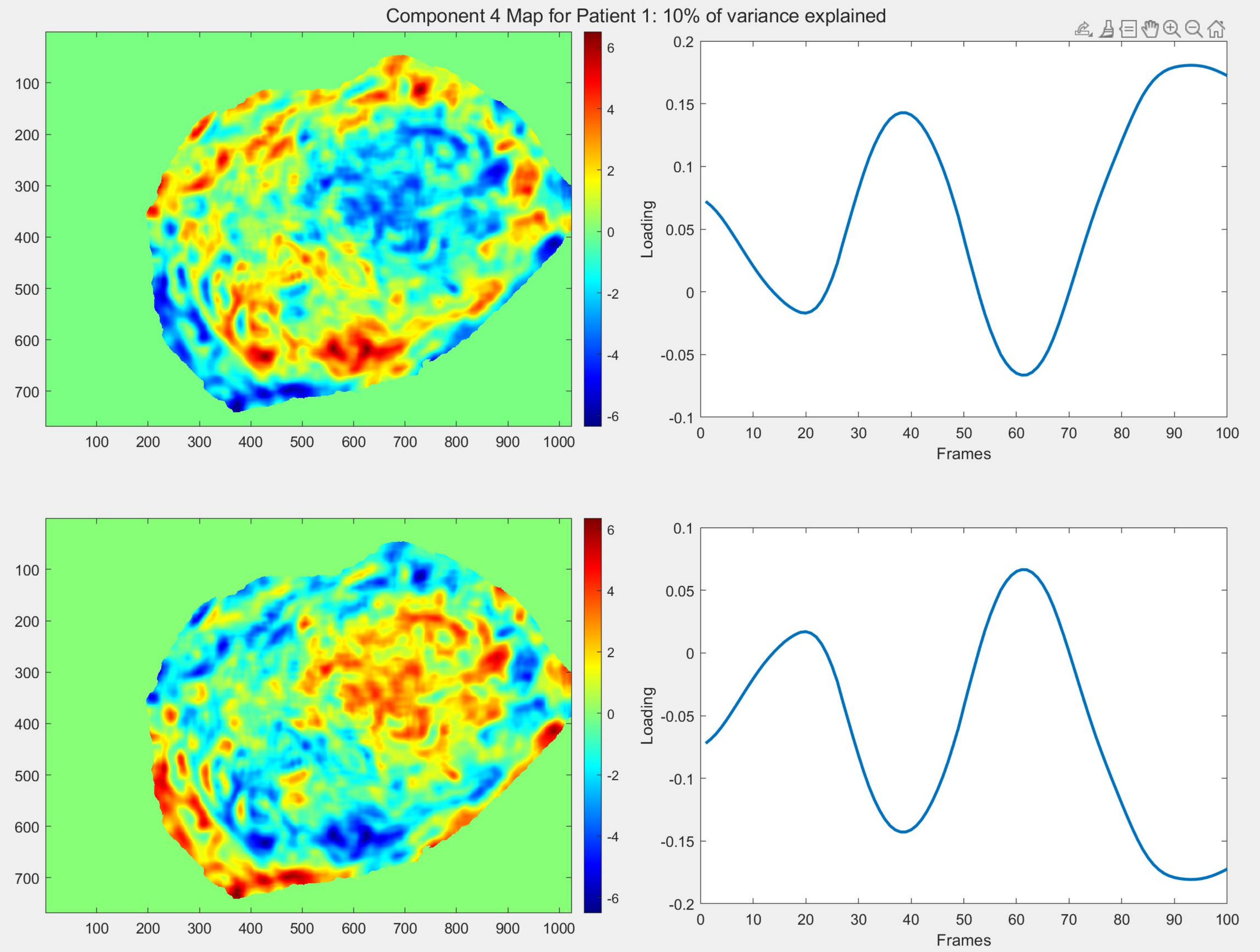
Component 4 Map for Patient 1: 10% of variance explained
Loading
Frames
Loading
Frames

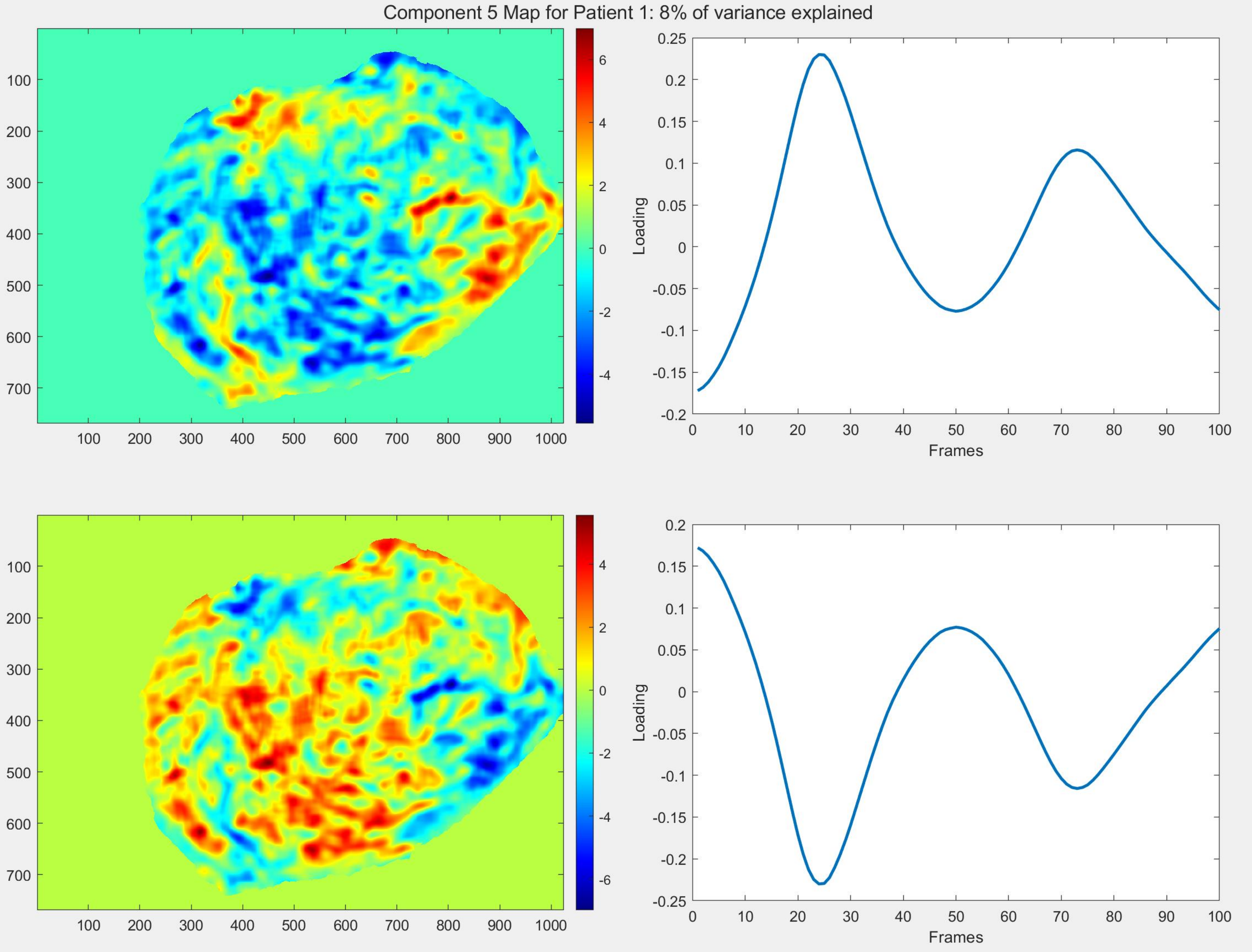

Component 5 Map for Patient 1: 8% of variance explained
Loading
Frames
Loading
Frames

Patient 3: Hand Motor

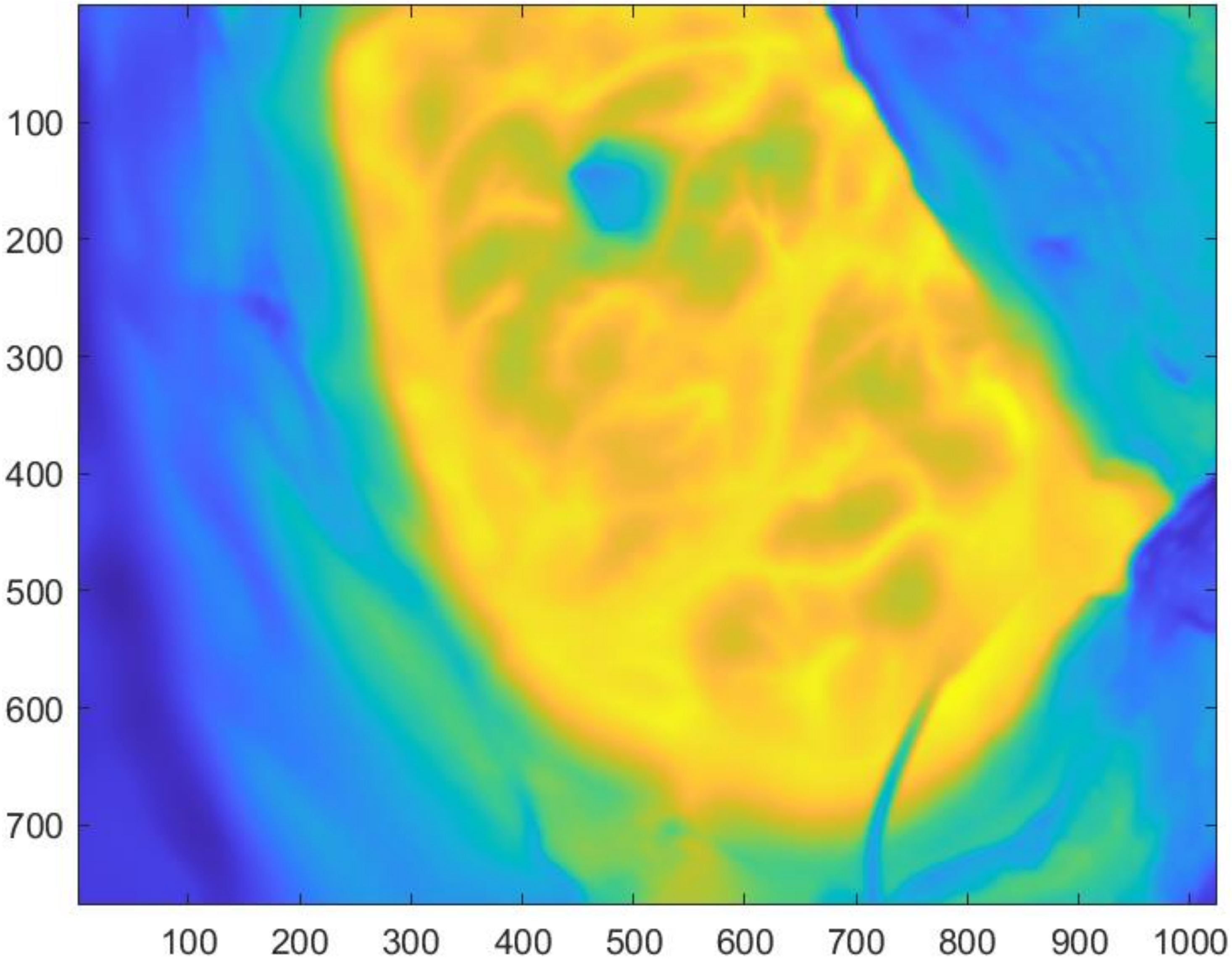
100
200
300
400
500
600
700
100
200
300
400
500
600
700
800
900
1000

P3 (Hand Motor) DES Key

- A – Middle finger tingling
- B – 2nd finger
- C – Thumb
- D – Left pinky / sensory
- **E – Left arm + hand motor**
- F – Left wrist
- 1 – wrist
- 2 – jaw sensation
- 3 – mouth motor
- 4 – lateral mouth motor

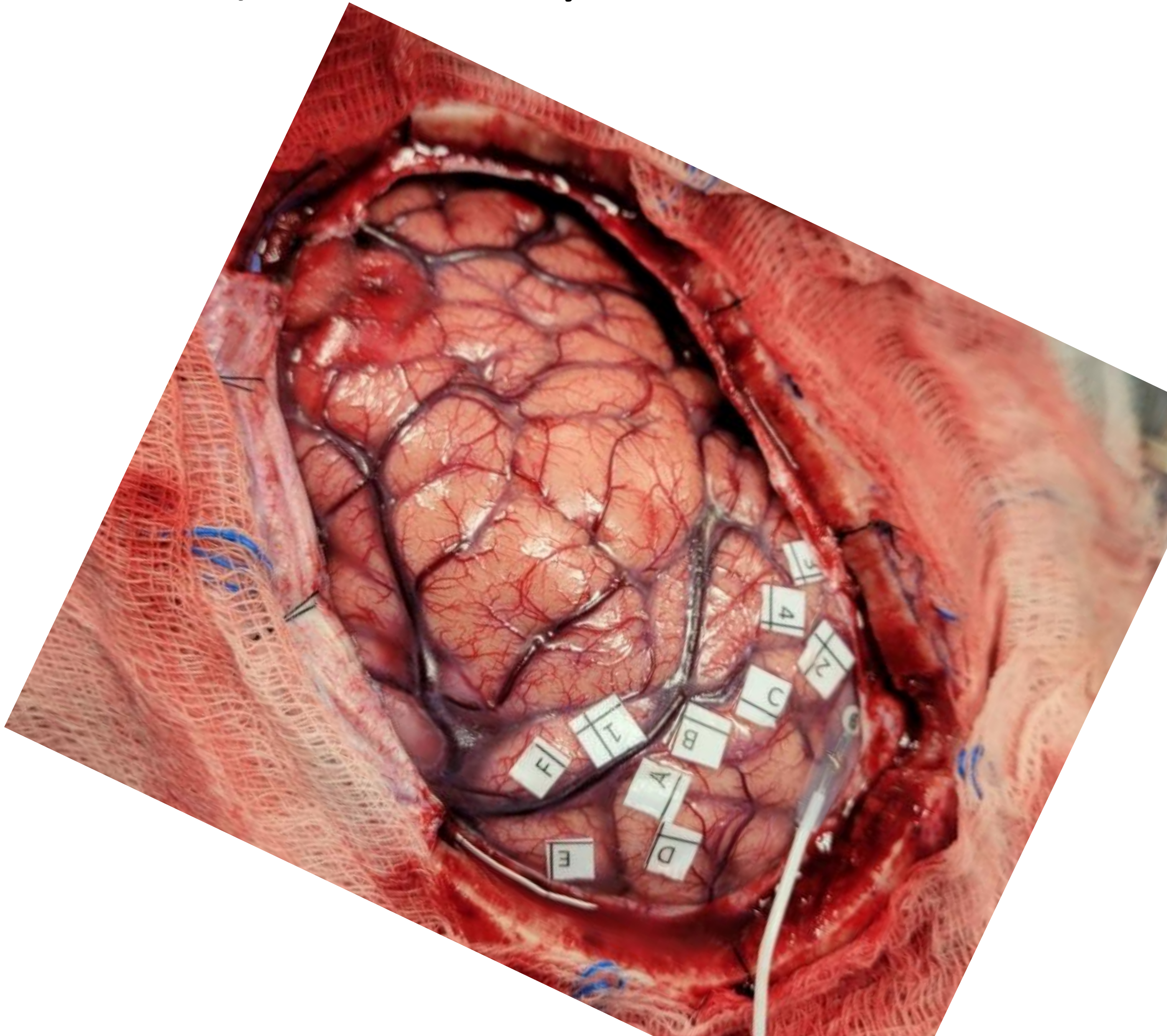

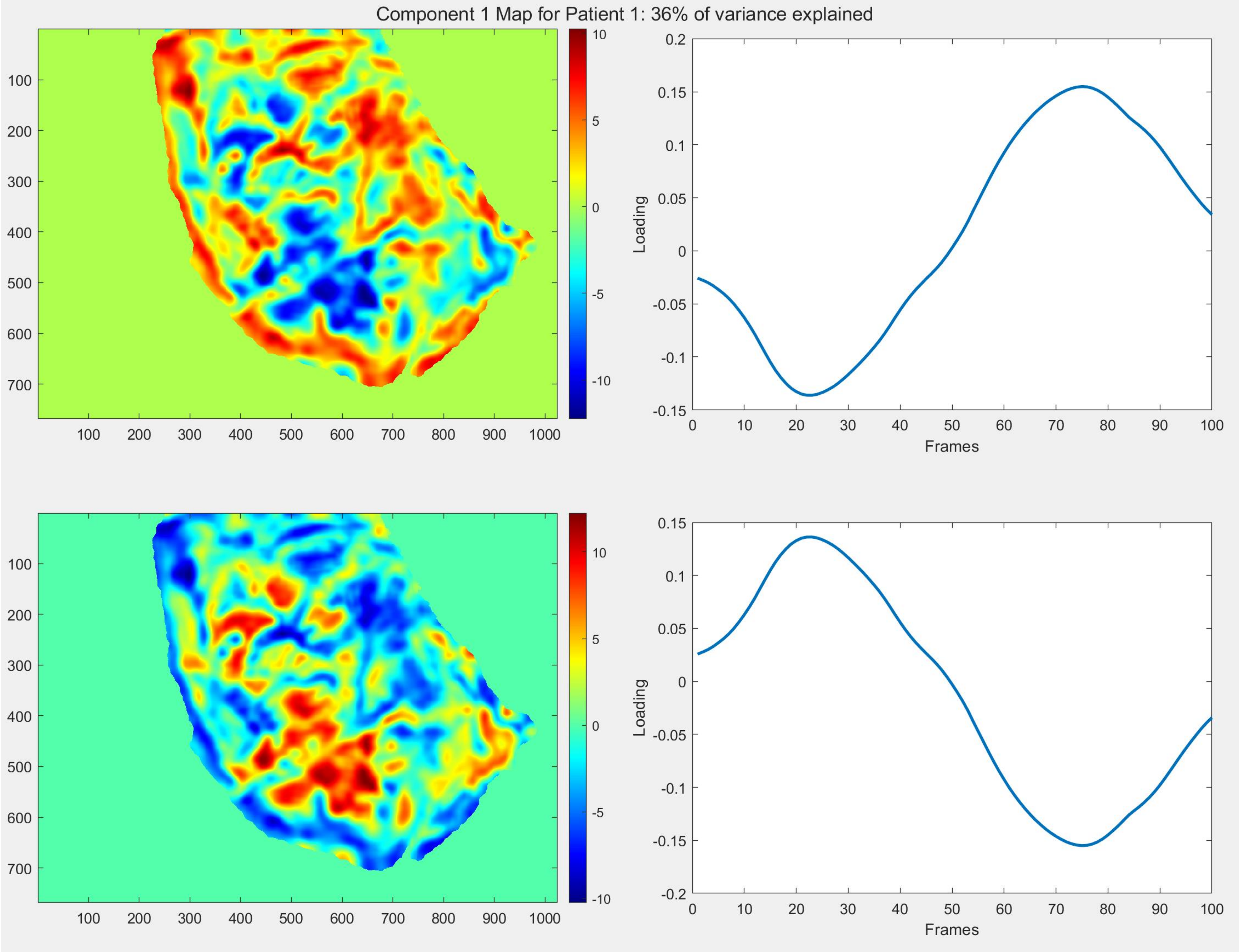
Component 1 Map for Patient 1: 36% of variance explained
Loading
Frames
Loading
Frames

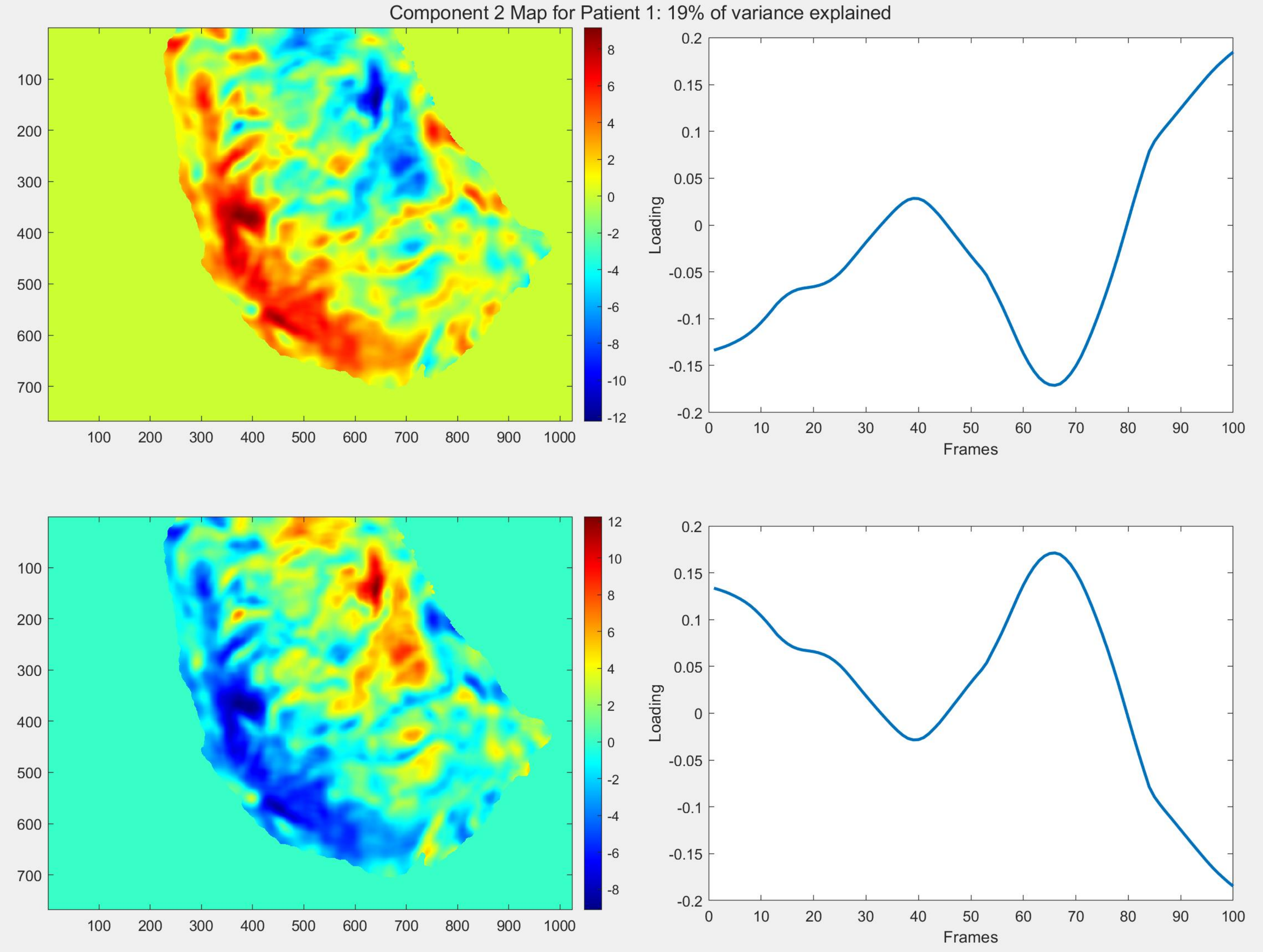

Component 2 Map for Patient 1: 19% of variance explained
Loading
Frames
Loading
Frames

Component 3 Map for Patient 1: 15% of variance explained

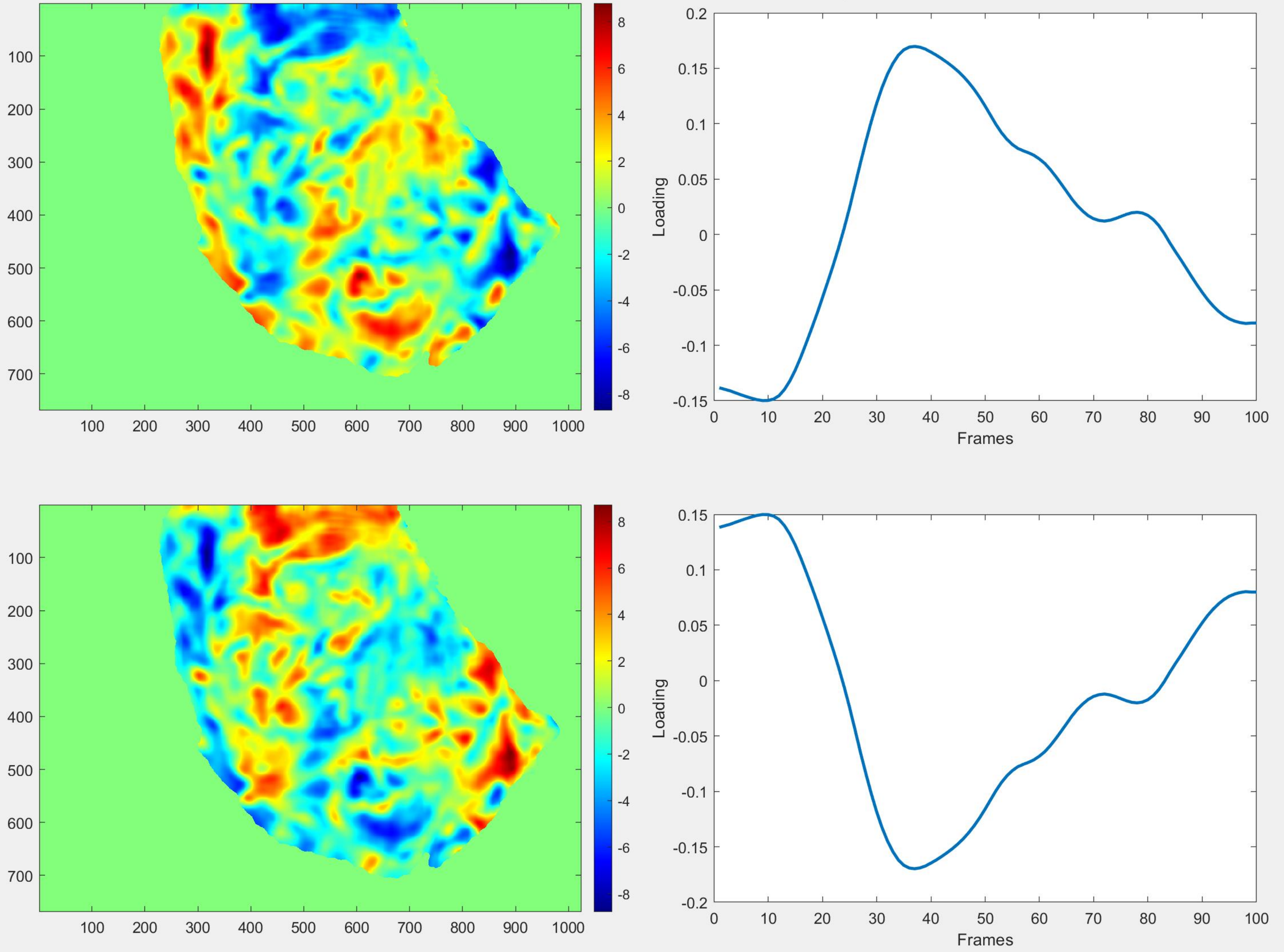

Component 4 Map for Patient 1: 9% of variance explained

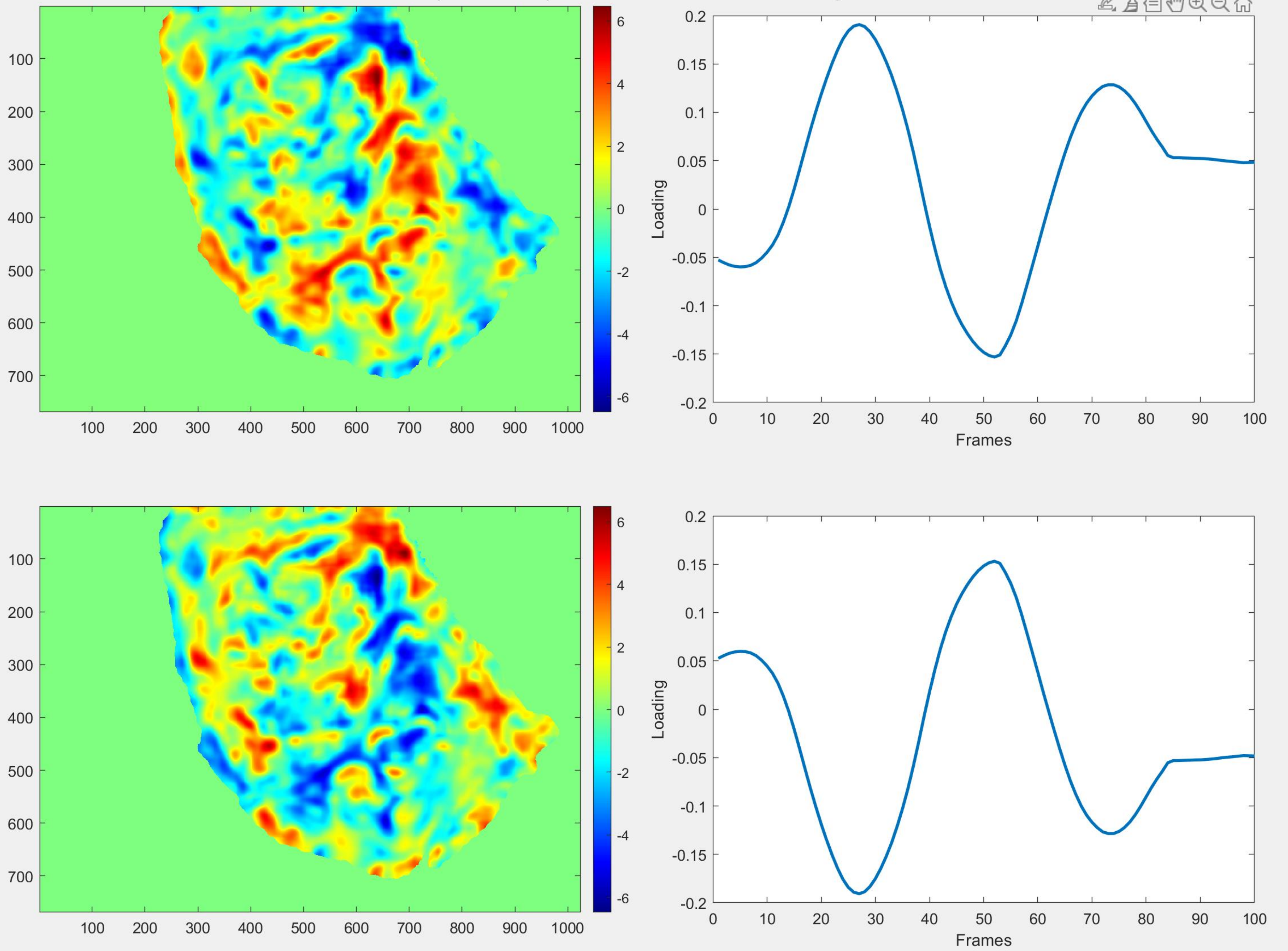

Component 5 Map for Patient 1: 7% of variance explained

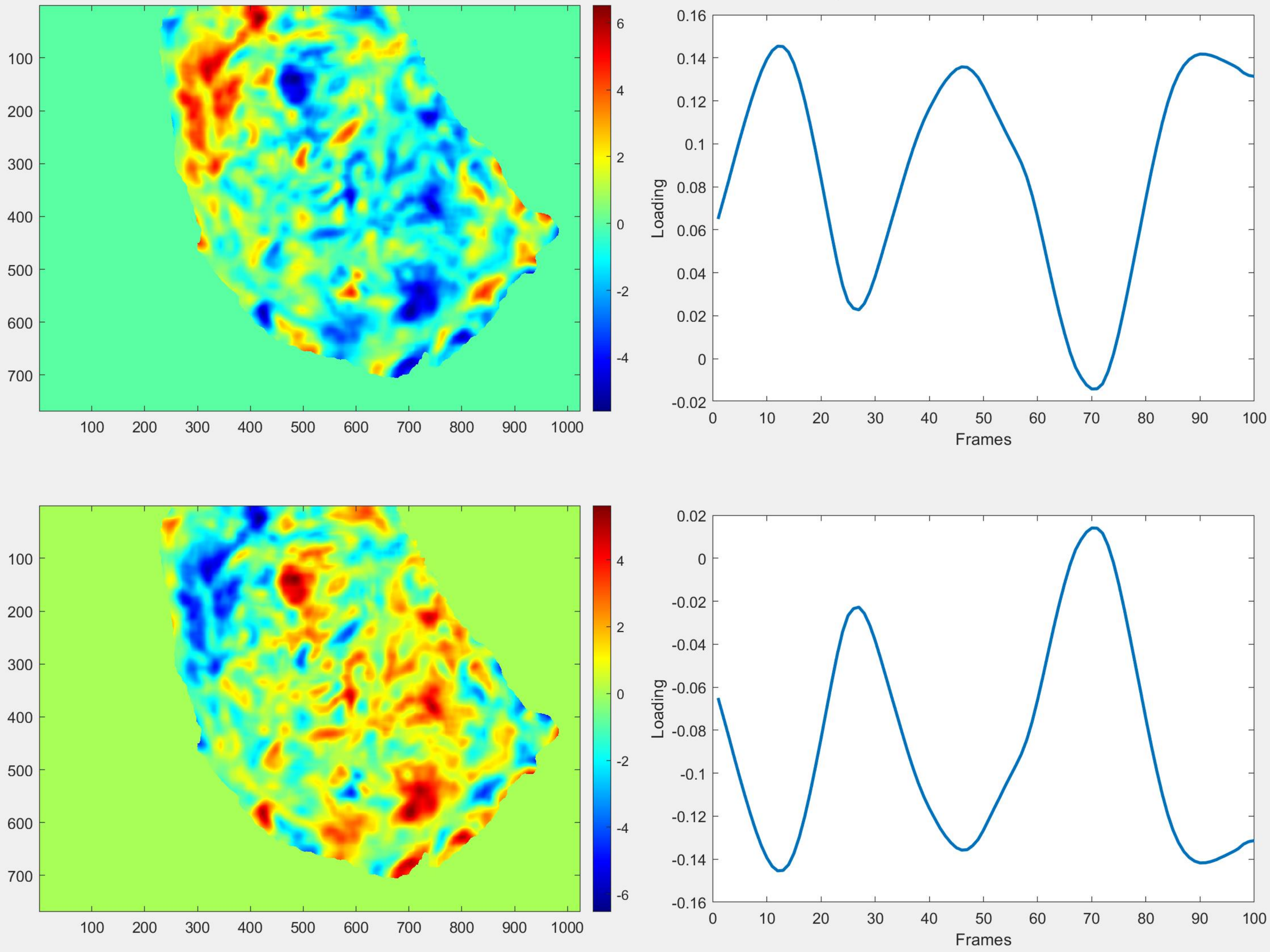

Patient 4: Hand Motor

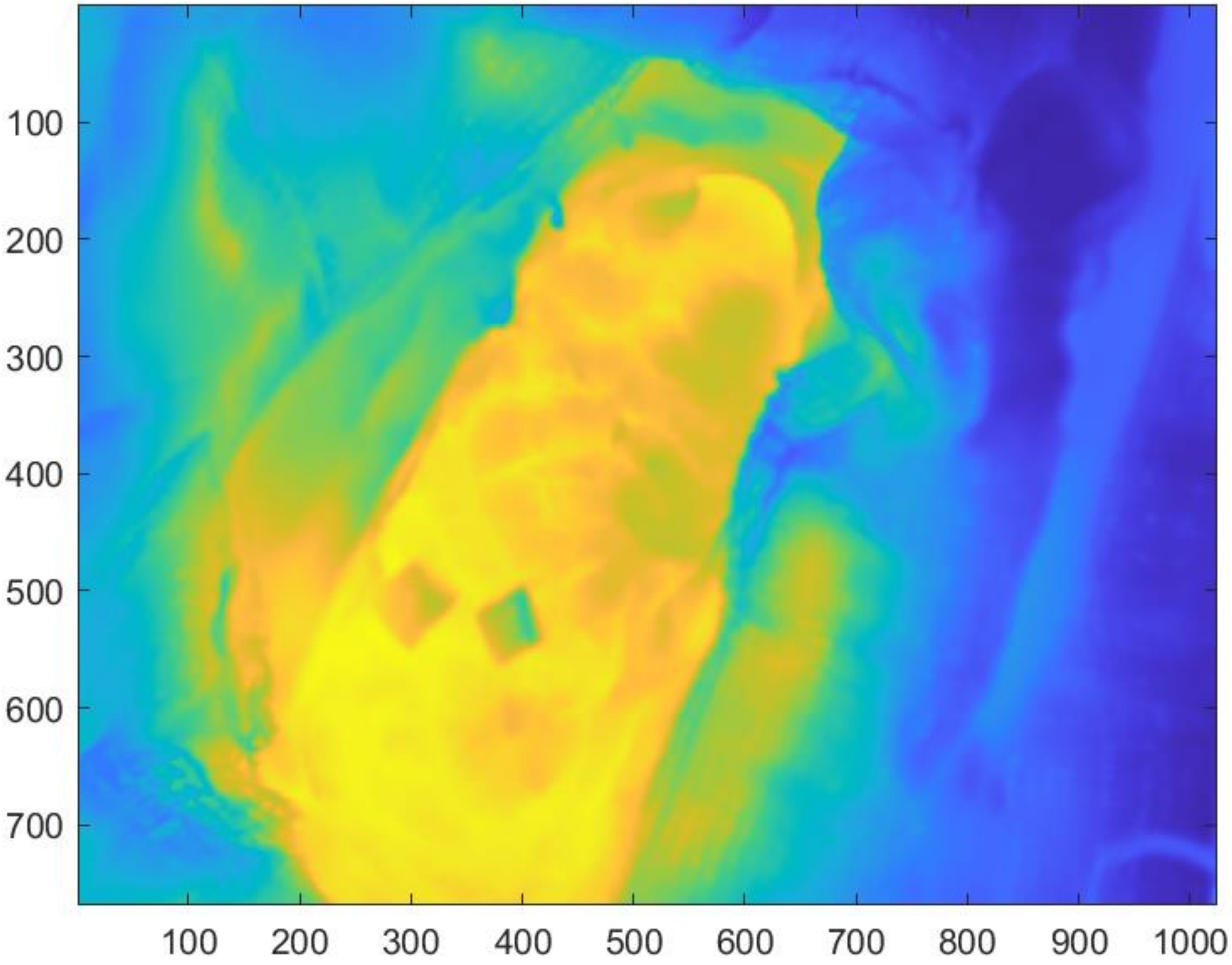
100
200
300
400
500
600
700
100
200
300
400
500
600
700
800
900
1000

P4 (Hand Motor) DES Key

- A – Finger Adduction
- B – Finger Extension
- C – Finger Sensory
- D – Finger Sensory
- E – Finger Sensory
- G – Thumb Sensory
- H – Wrist Motor
- I – Forearm Motor
- J – Hand Motor

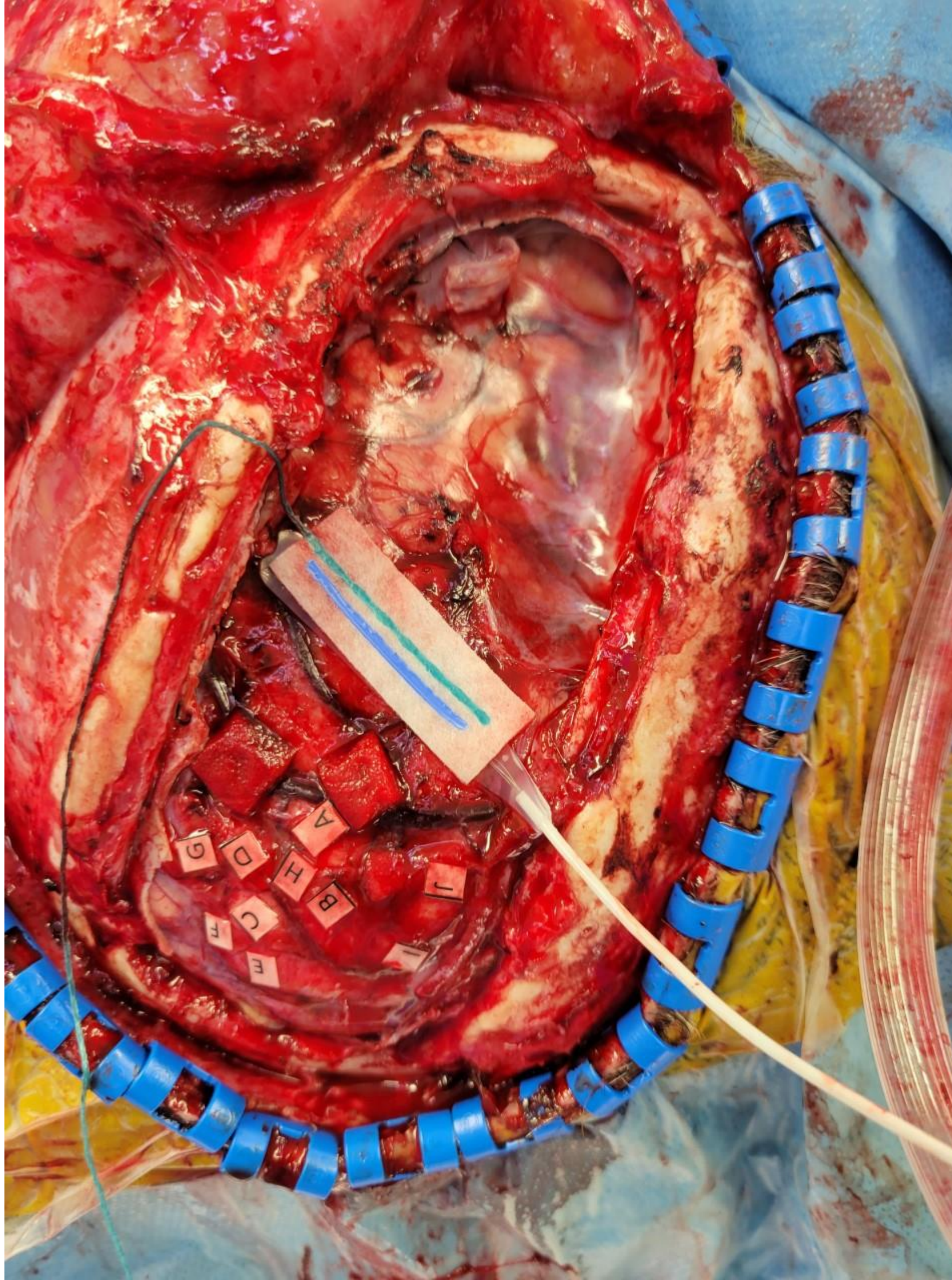

Patient 5: Hand Sensory

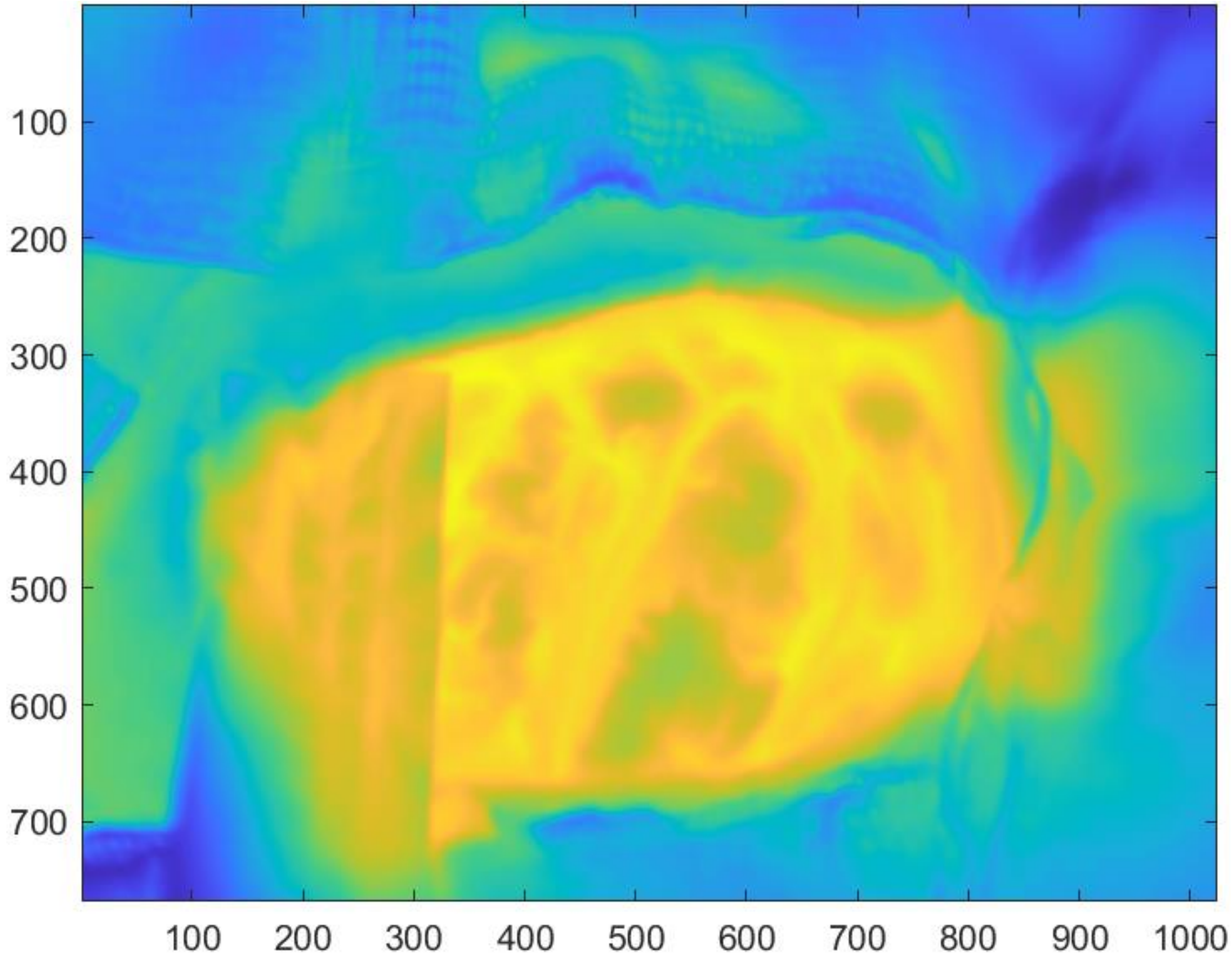
100
200
300
400
500
600
700
100
200
300
400
500
600
700
800
900
1000

1. Forearm Sensory
2. Forearm Tingling
3. Left Palm Tingling
4. 3,4,5 Finger Flexion
5. **Finger Tingling**
6. Left Upper Arm Tingling
7. Torso Tingling (left)
8. Left Upper Side Tingling
9. Left Wrist / Arm Movement
10. Left Wrist Flexion
11. Face Tinging
12. Elbow Flexion
13. Left Shoulder Movement

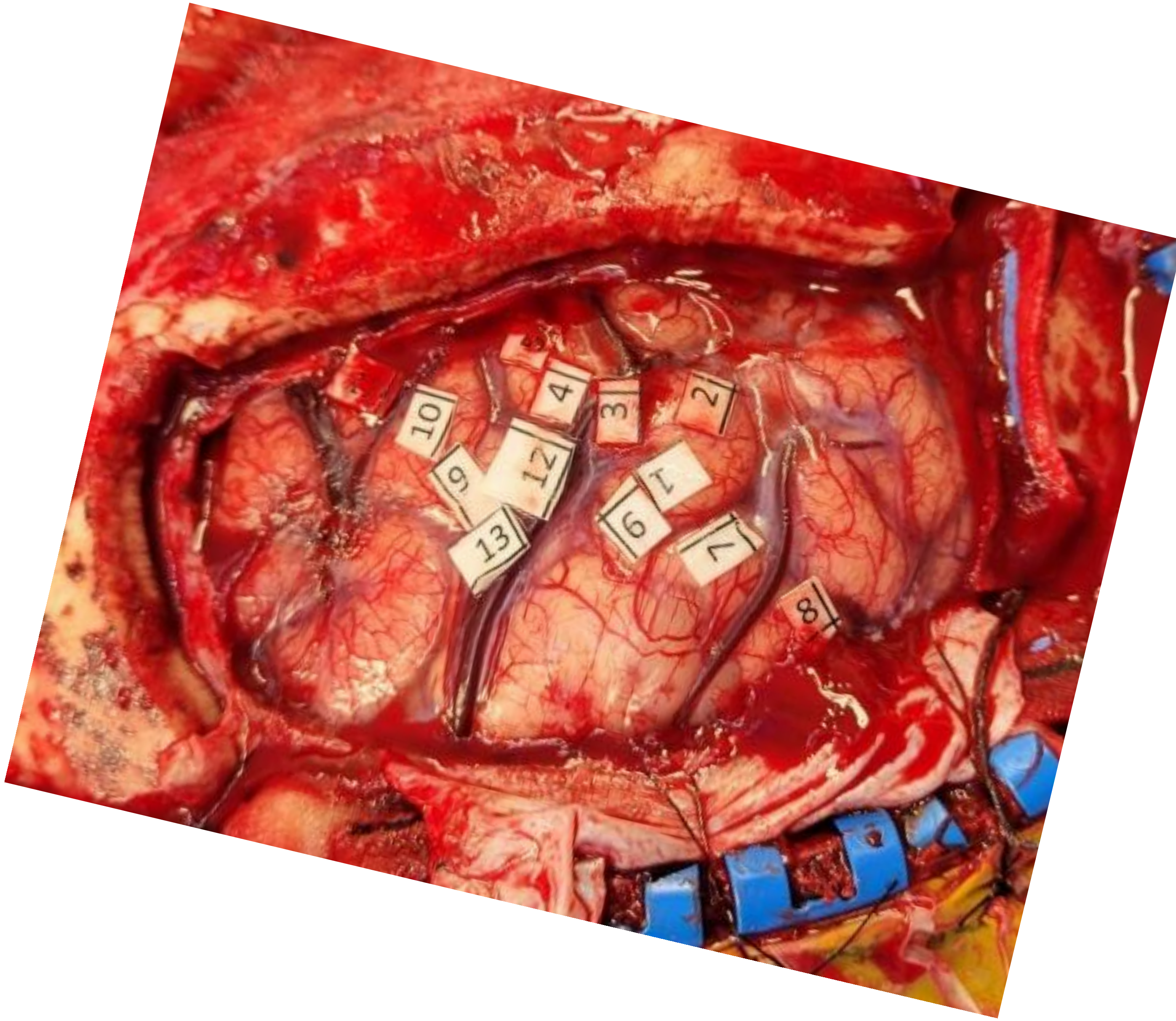

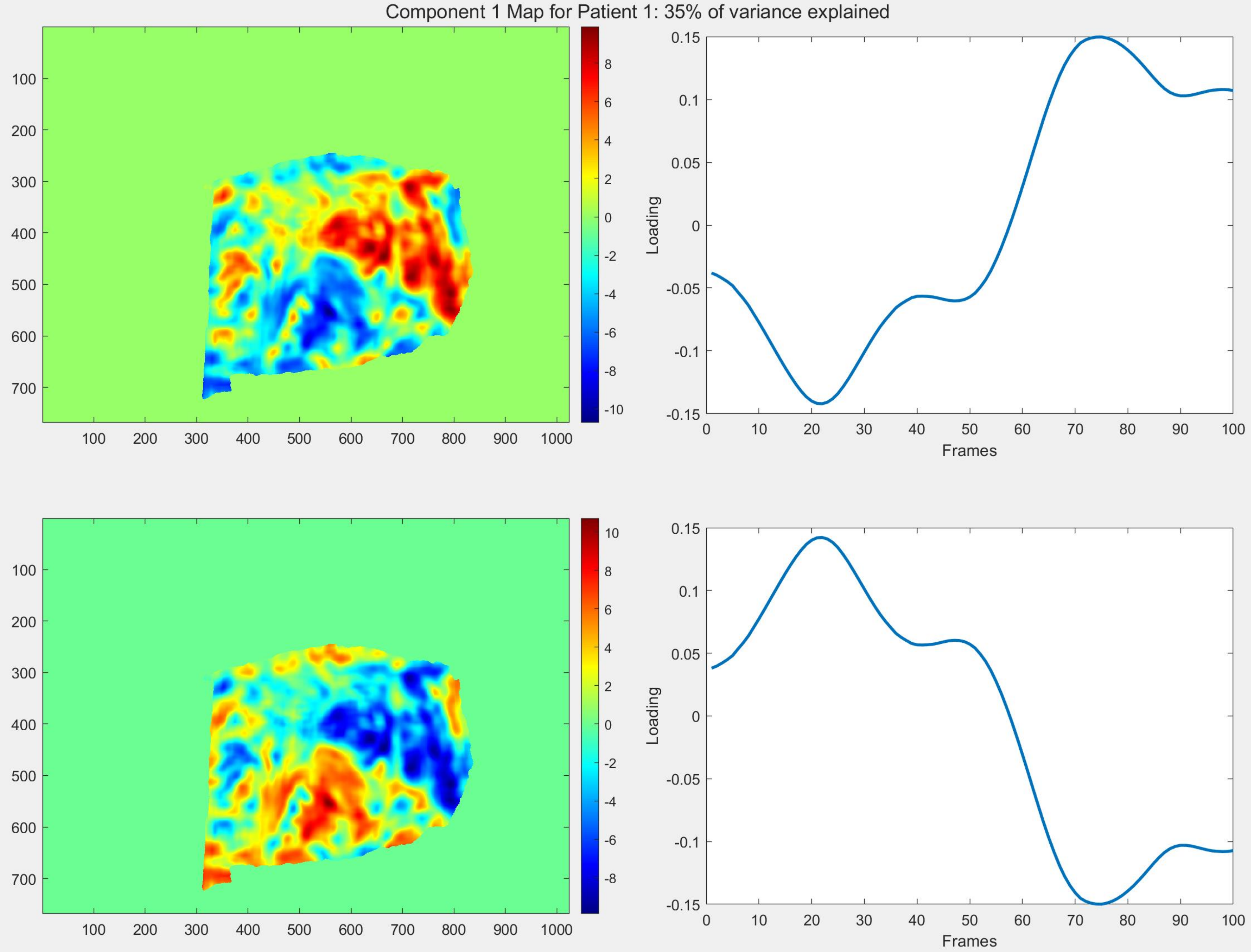

Component 1 Map for Patient 1: 35% of variance explained
Loading
Frames
Loading
Frames

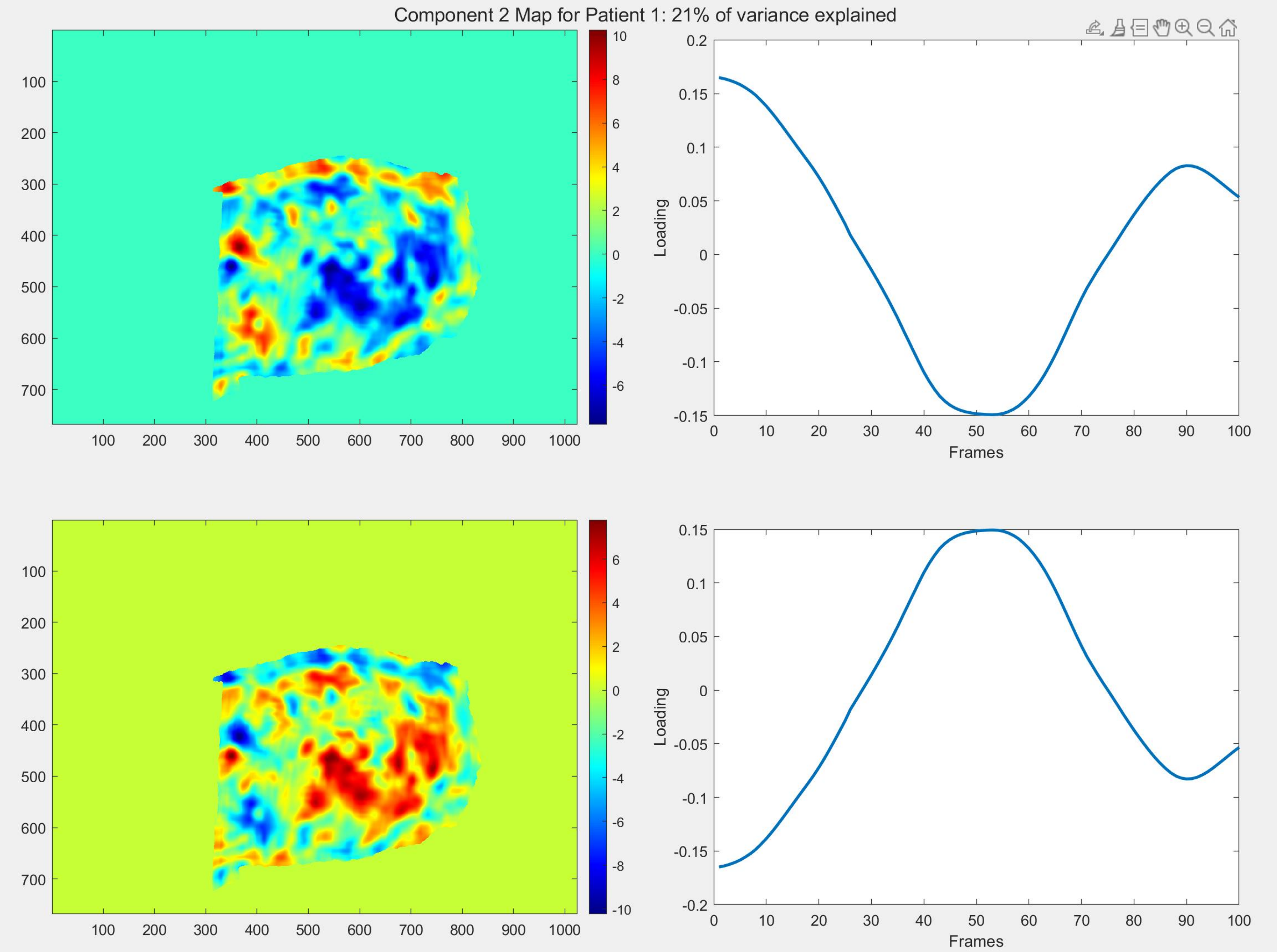
Component 2 Map for Patient 1: 21% of variance explained
Loading
Frames
Loading
Frames

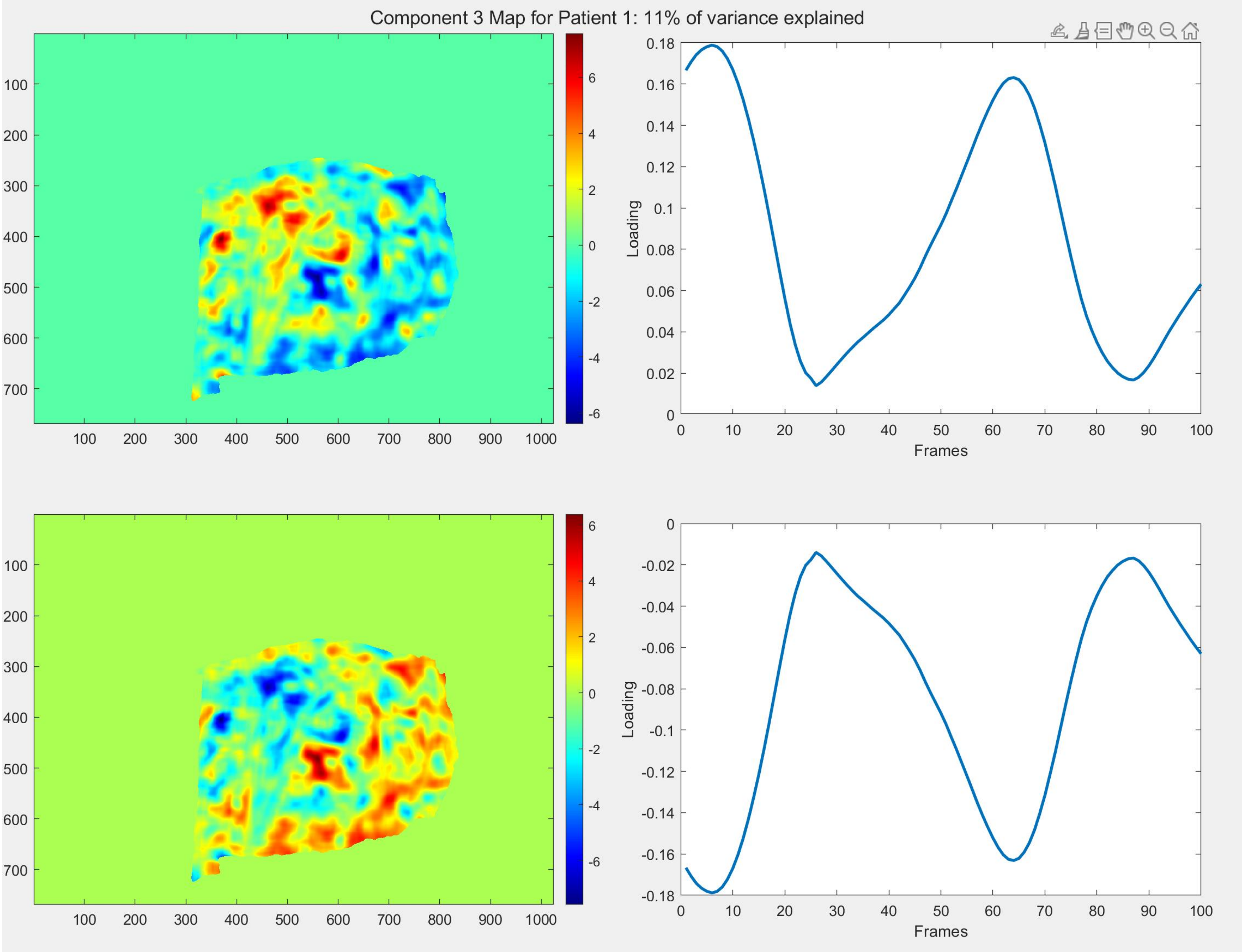

Component 3 Map for Patient 1: 11% of variance explained
Loading
Frames

Component 4 Map for Patient 1: 11% of variance explained

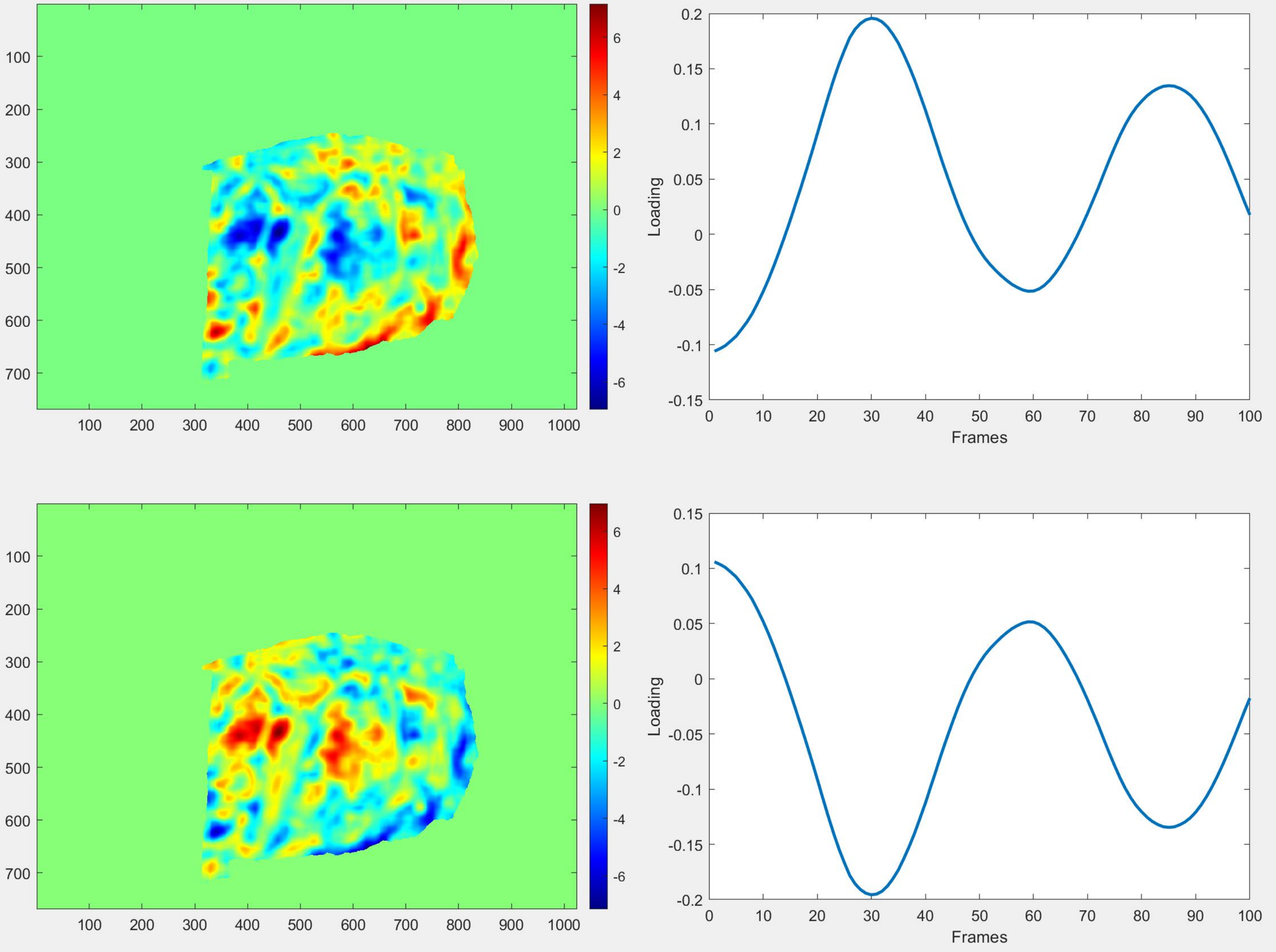

Component 5 Map for Patient 1: 8% of variance explained

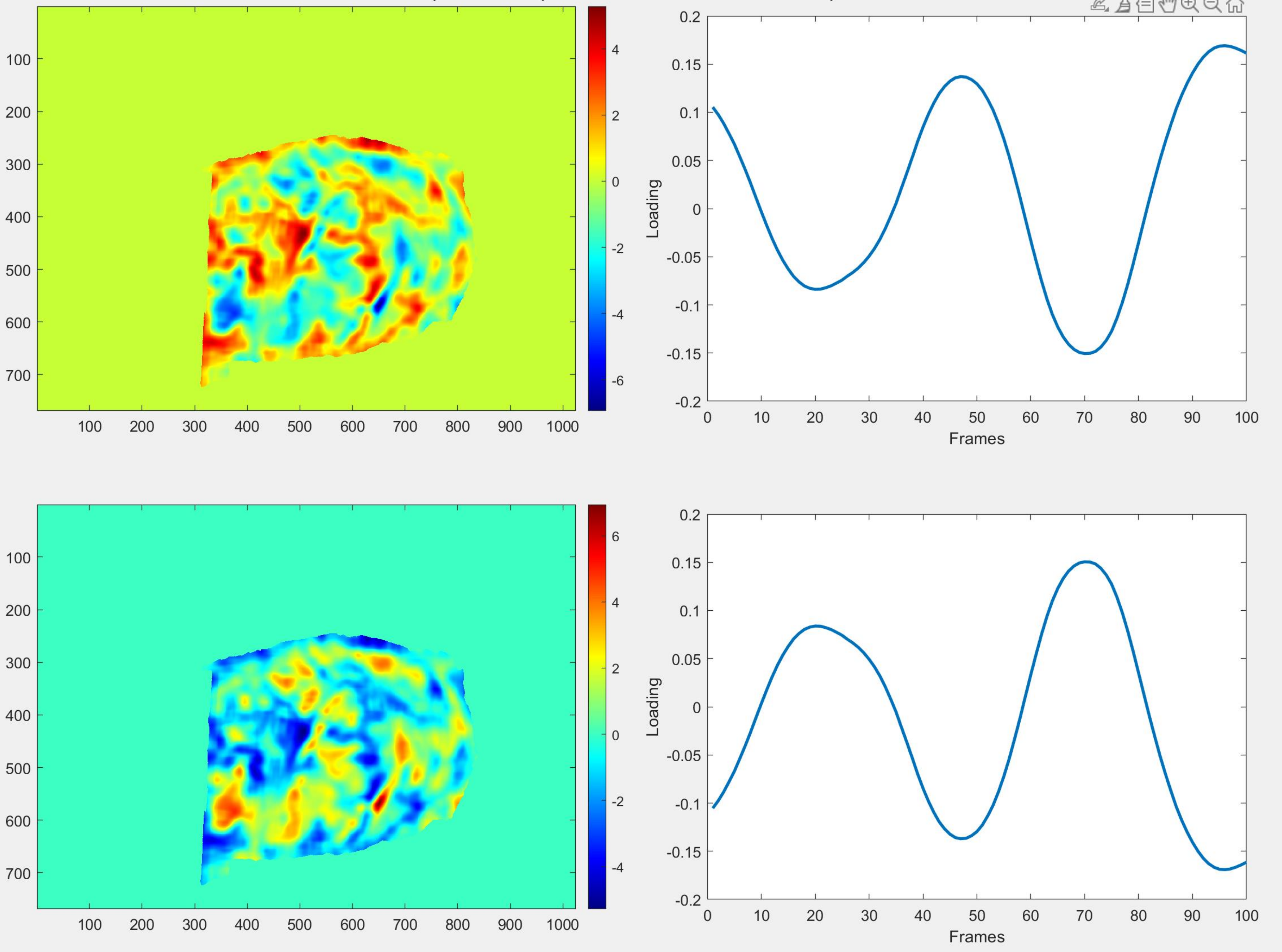

Patient 6: Hand Sensory

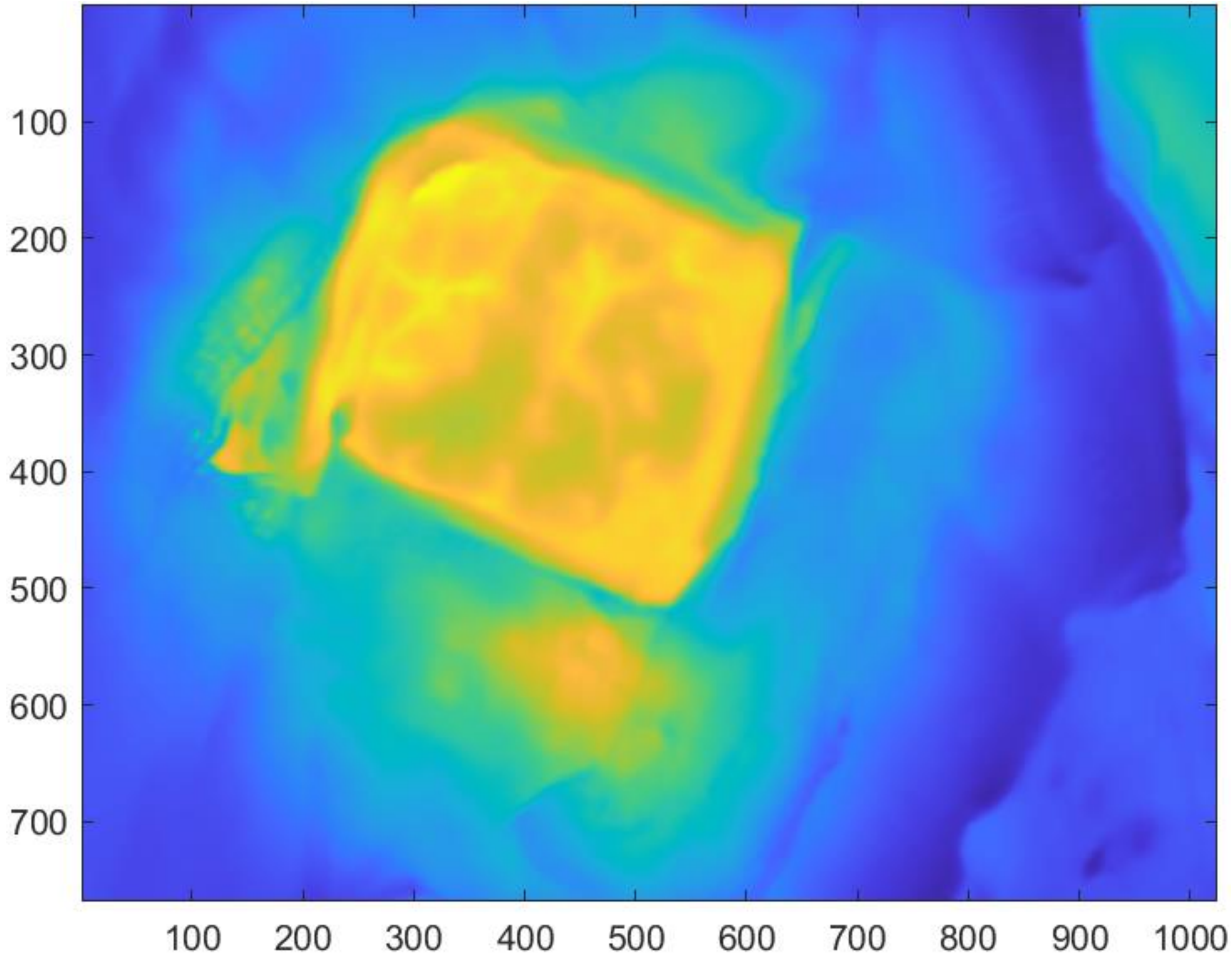
100
200
300
400
500
600
700
100
200
300
400
500
600
700
800
900
1000

P6 (Hand Sensory) DES Key

- A – Left finger extension
- B – Left hand extension
- C – Cheek tingling
- D – Thumb numbness
- E – Pinky Numbness

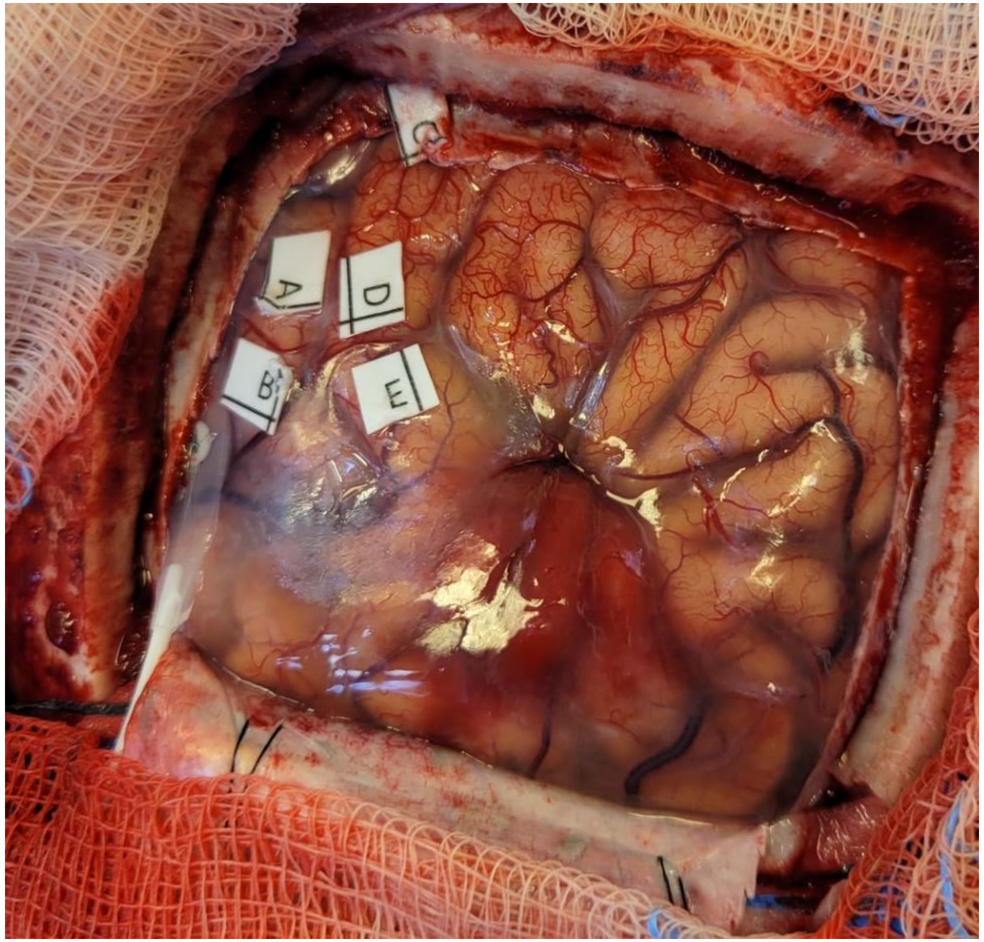

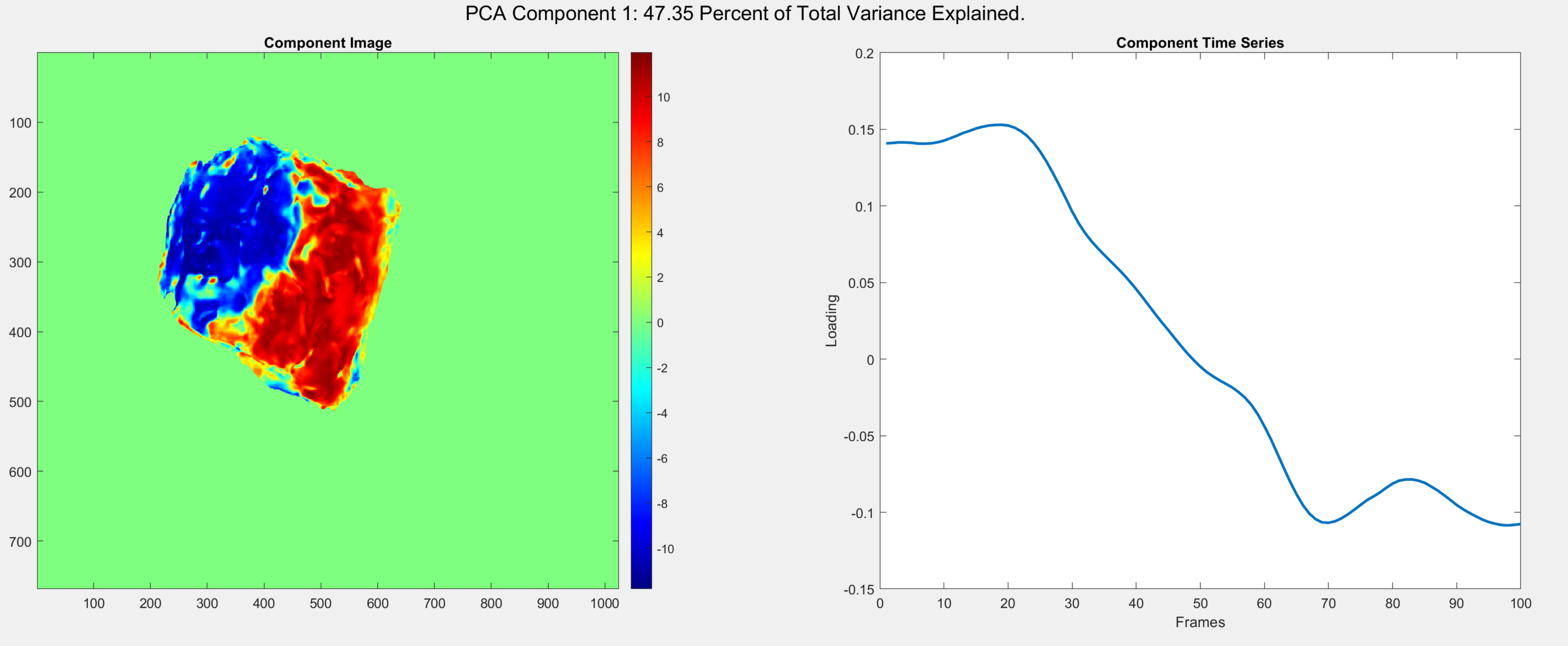
PCA Component 1: 47.35 Percent of Total Variance Explained.
Component Image
Component Time Series
Loading
Frames

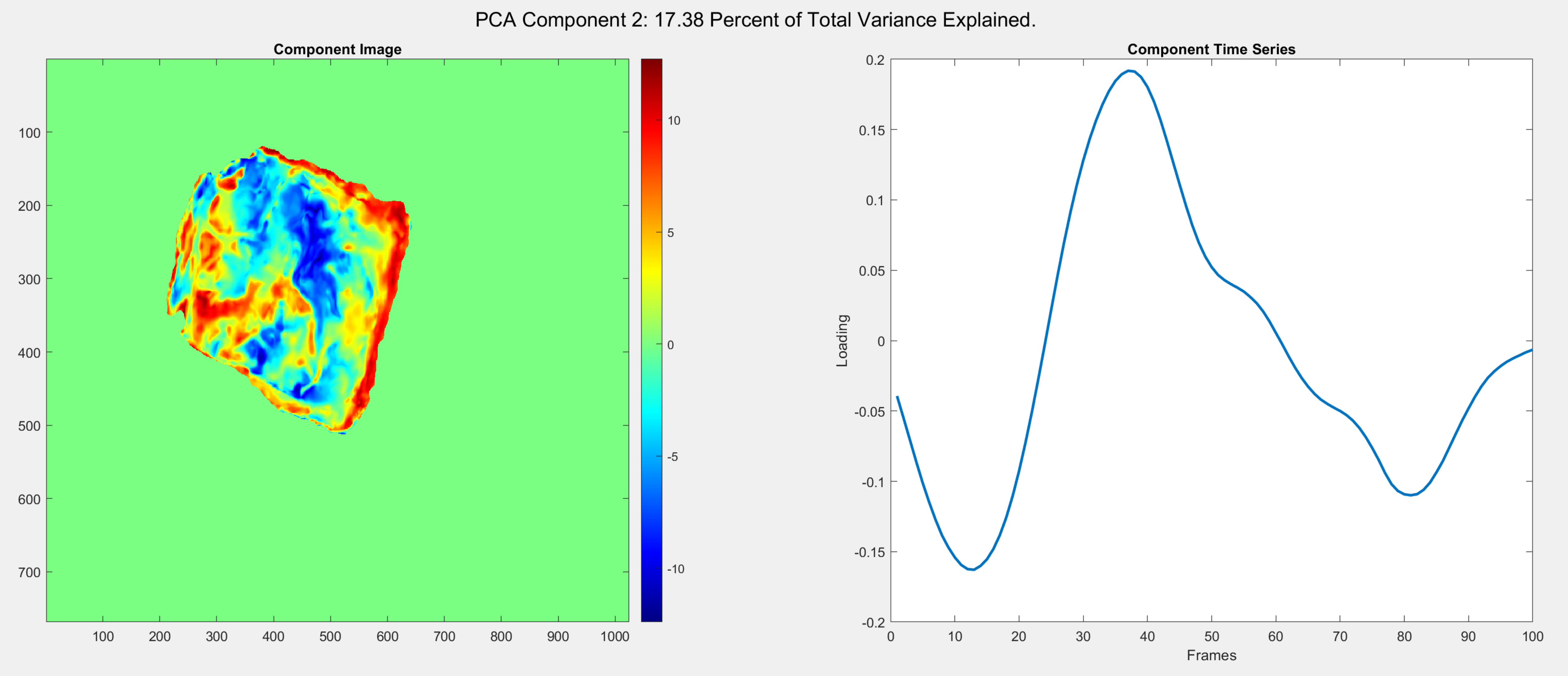

PCA Component 2: 17.38 Percent of Total Variance Explained.
Component Image
Component Time Series
Loading
Frames

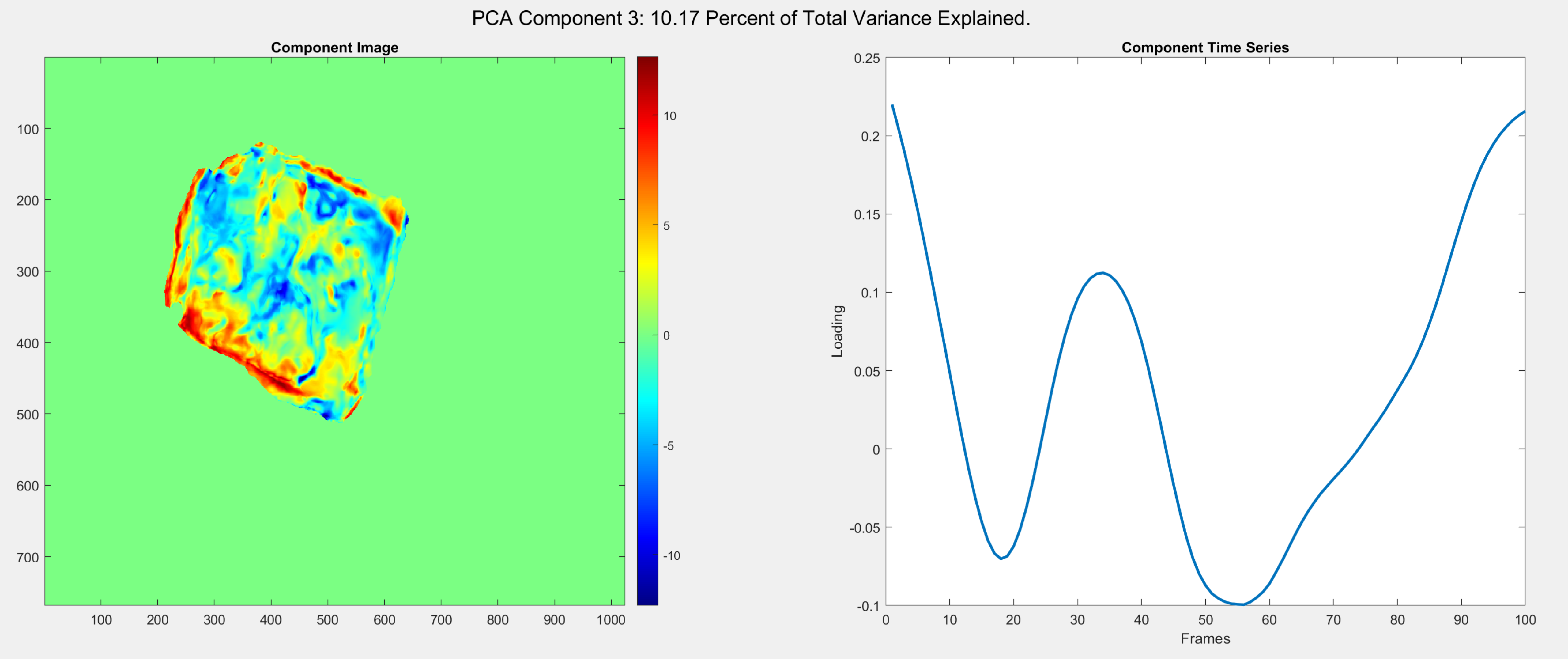
PCA Component 3: 10.17 Percent of Total Variance Explained.
Component Image
Component Time Series
Loading
Frames
100
200
300
400
500
600
700
800
900
1000
10
5
0
-5
-10
0.25
0.2
0.15
0.1
0.05
-0.05
-0.1
10
20
30
40
50
60
70
80
90

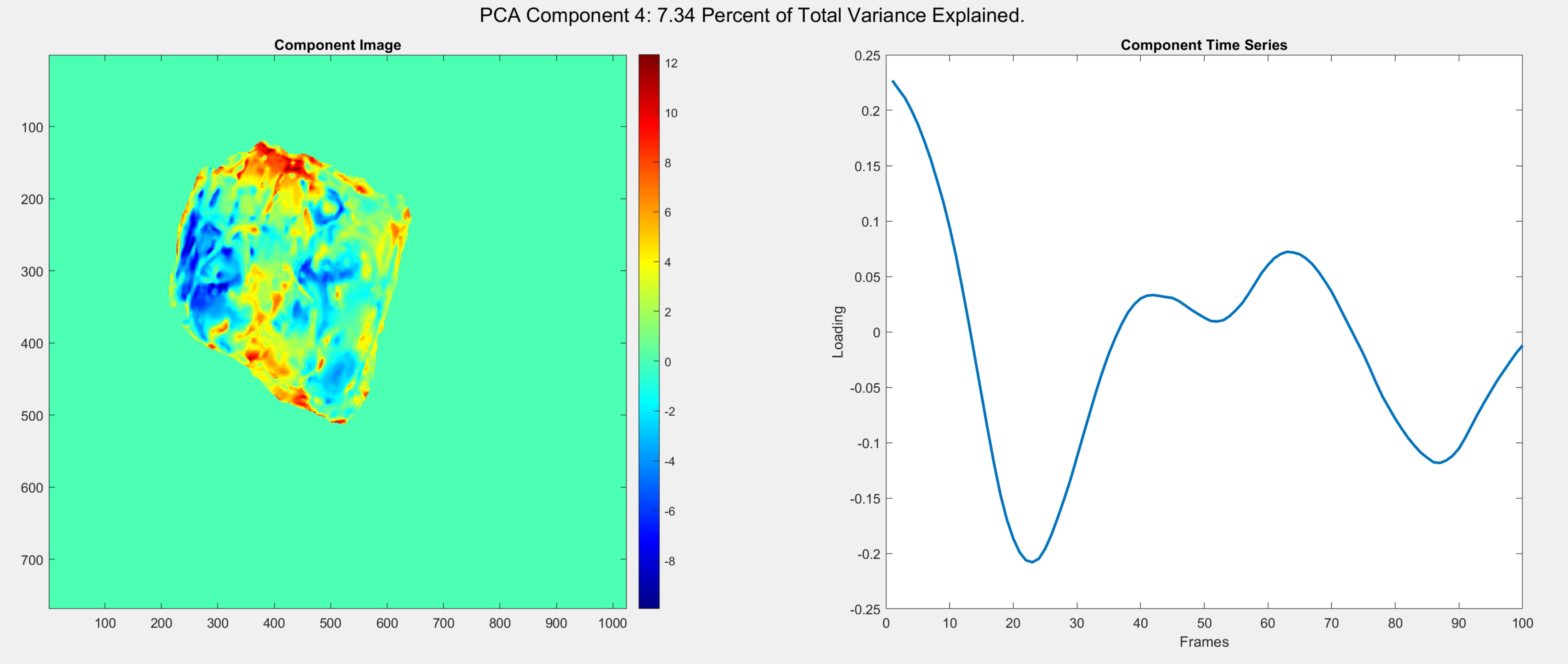
PCA Component 4: 7.34 Percent of Total Variance Explained.
Component Image
100
200
300
400
500
600
700
100
200
300
400
500
600
700
800
900
1000
12
10
8
6
4
2
0
-2
-4
-6
-8
Component Time Series
0.25
0.2
0.15
0.1
0.05
0
-0.05
-0.1
-0.15
-0.2
-0.25
Loading
0
10
20
30
40
50
60
70
80
90
100
Frames

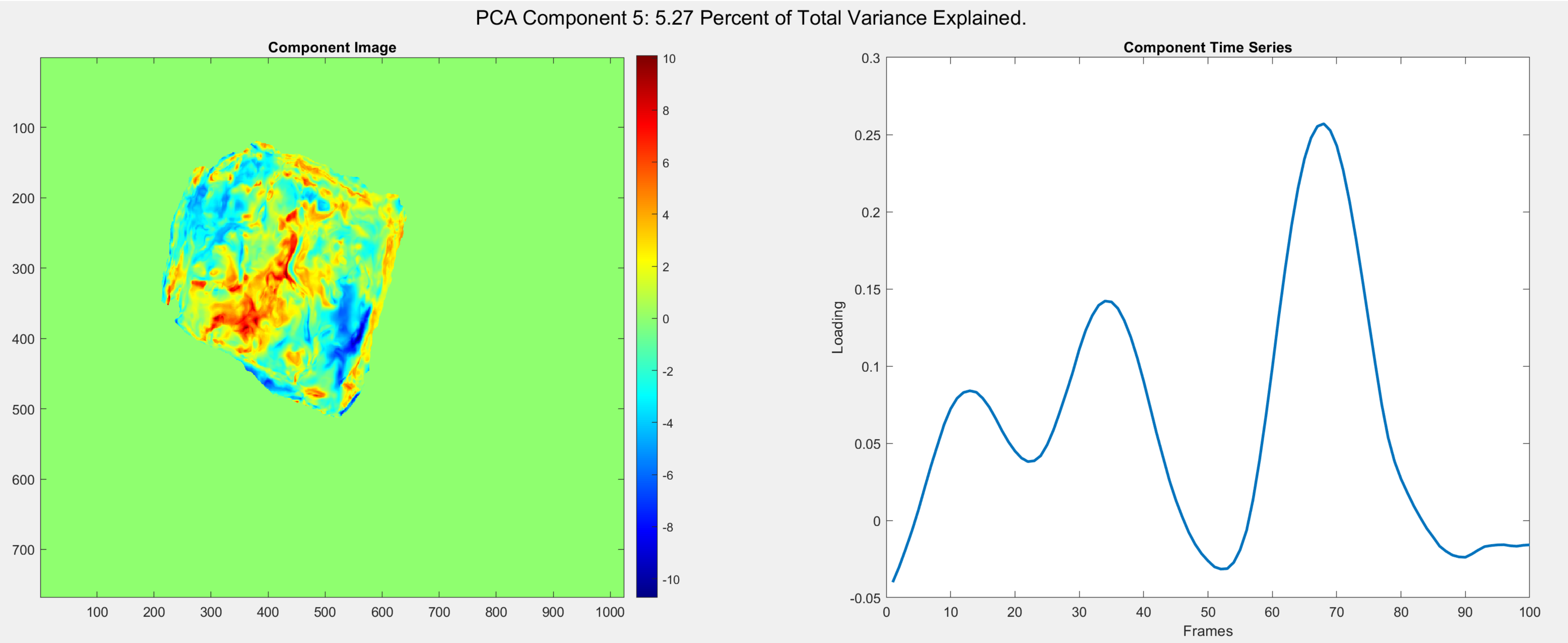
PCA Component 5: 5.27 Percent of Total Variance Explained.
Component Image
Component Time Series
Loading
Frames

Part 2: Group PCA
Time Series

Component 1 Time Series: 34% of Variance

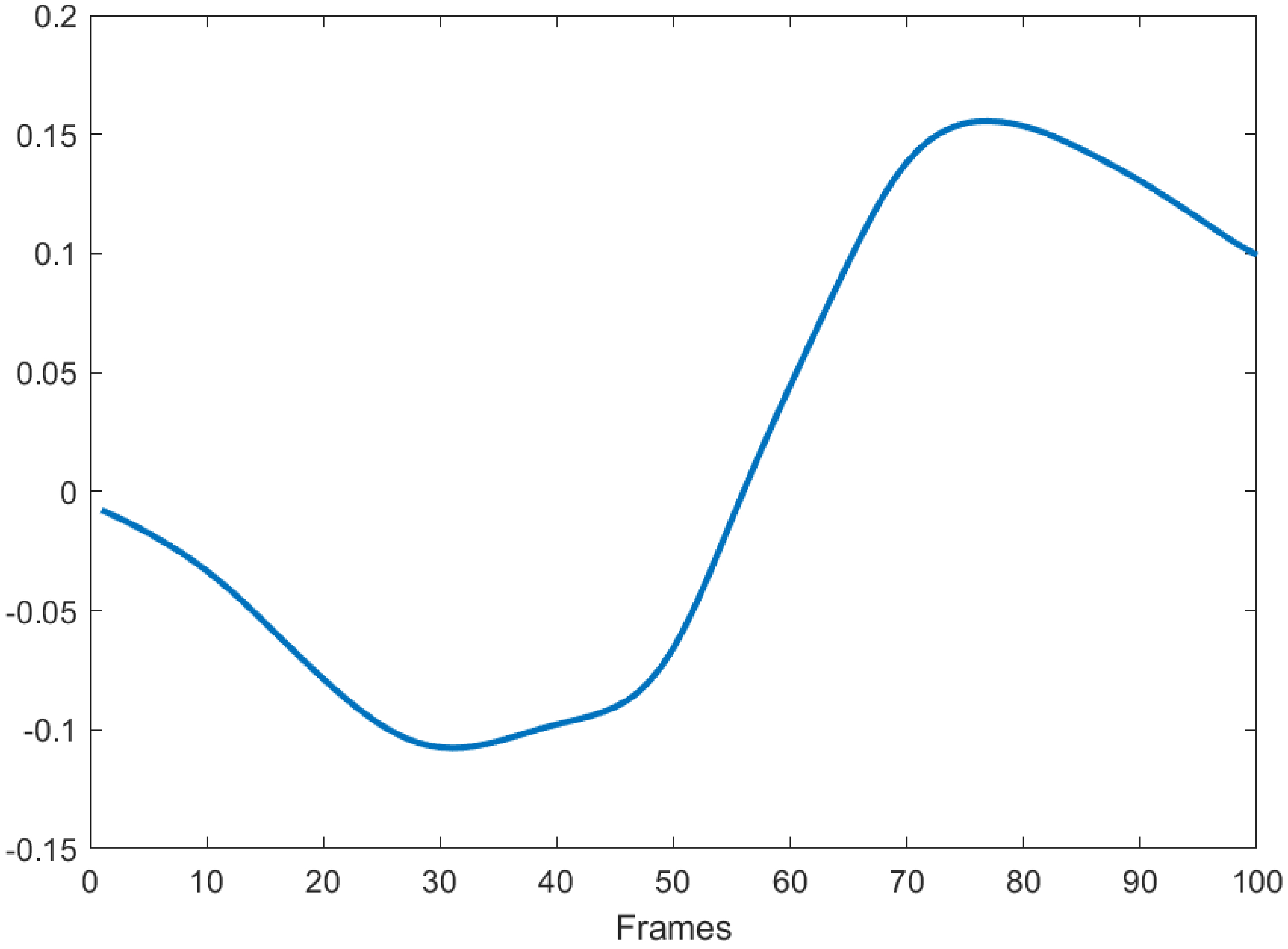

Component 2 Time Series: 19% of Variance

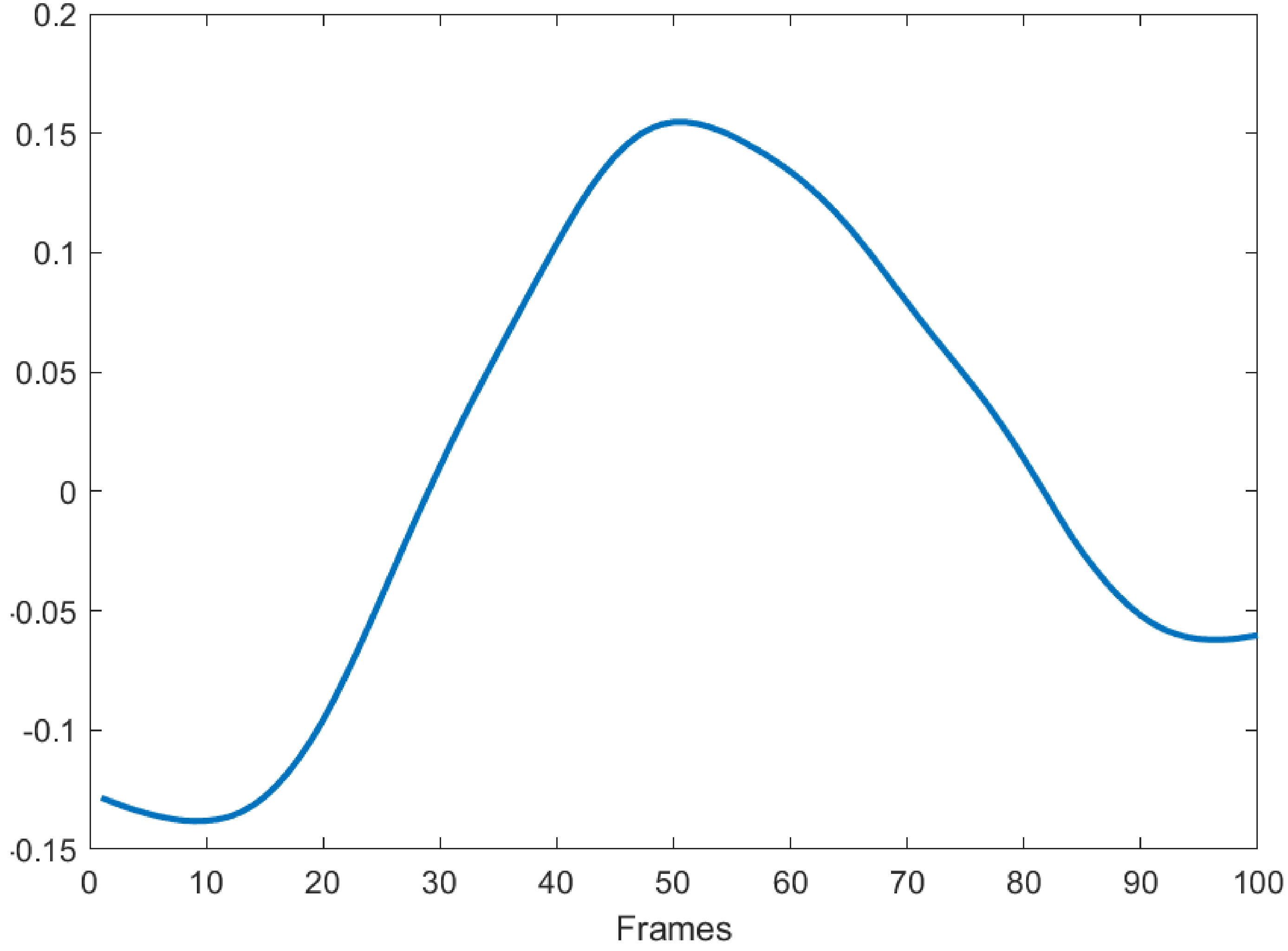

Component 3 Time Series: 13% of Variance

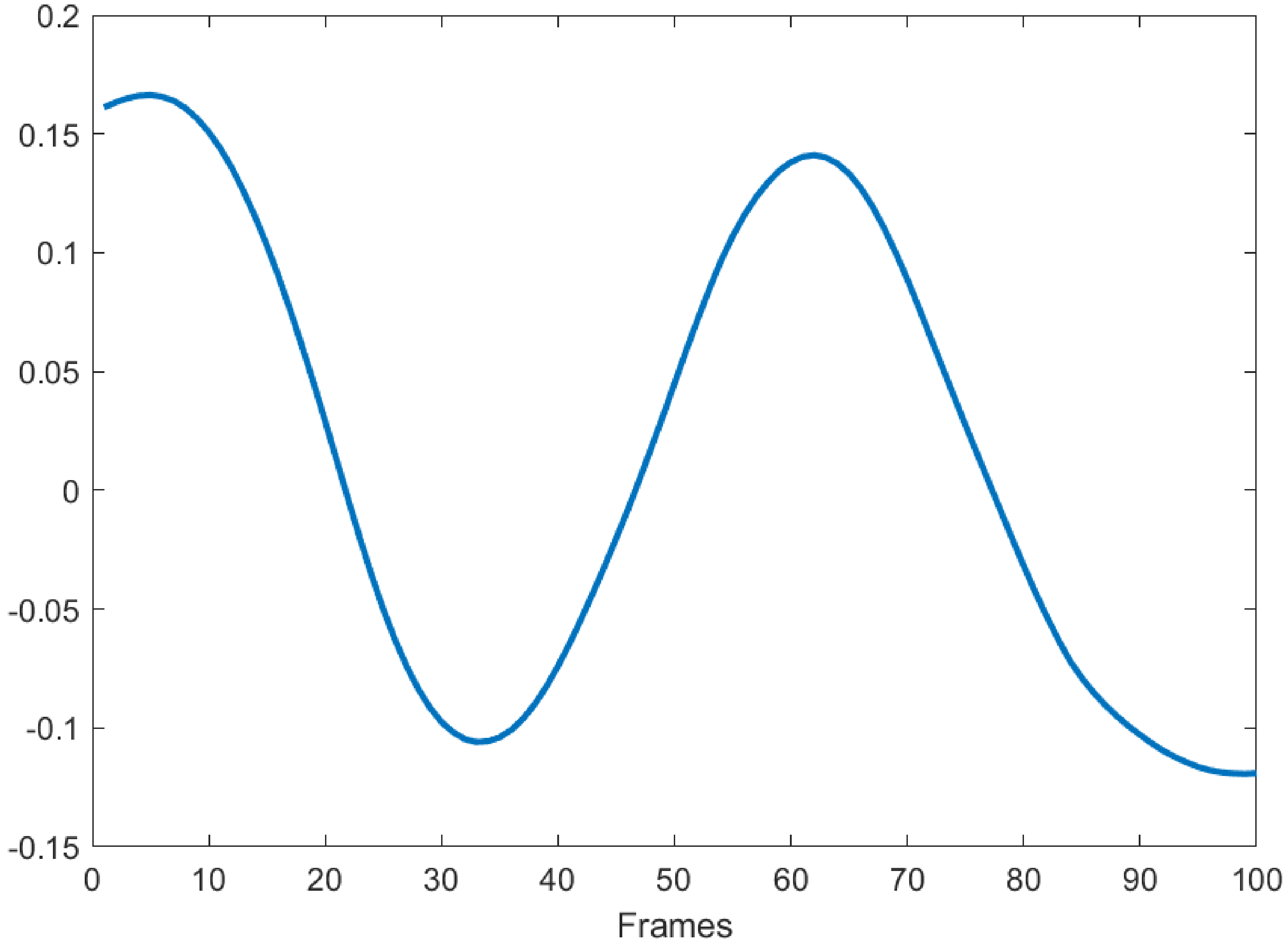

Part 3: Group PCA Components

Patient 1: Lip Pursing

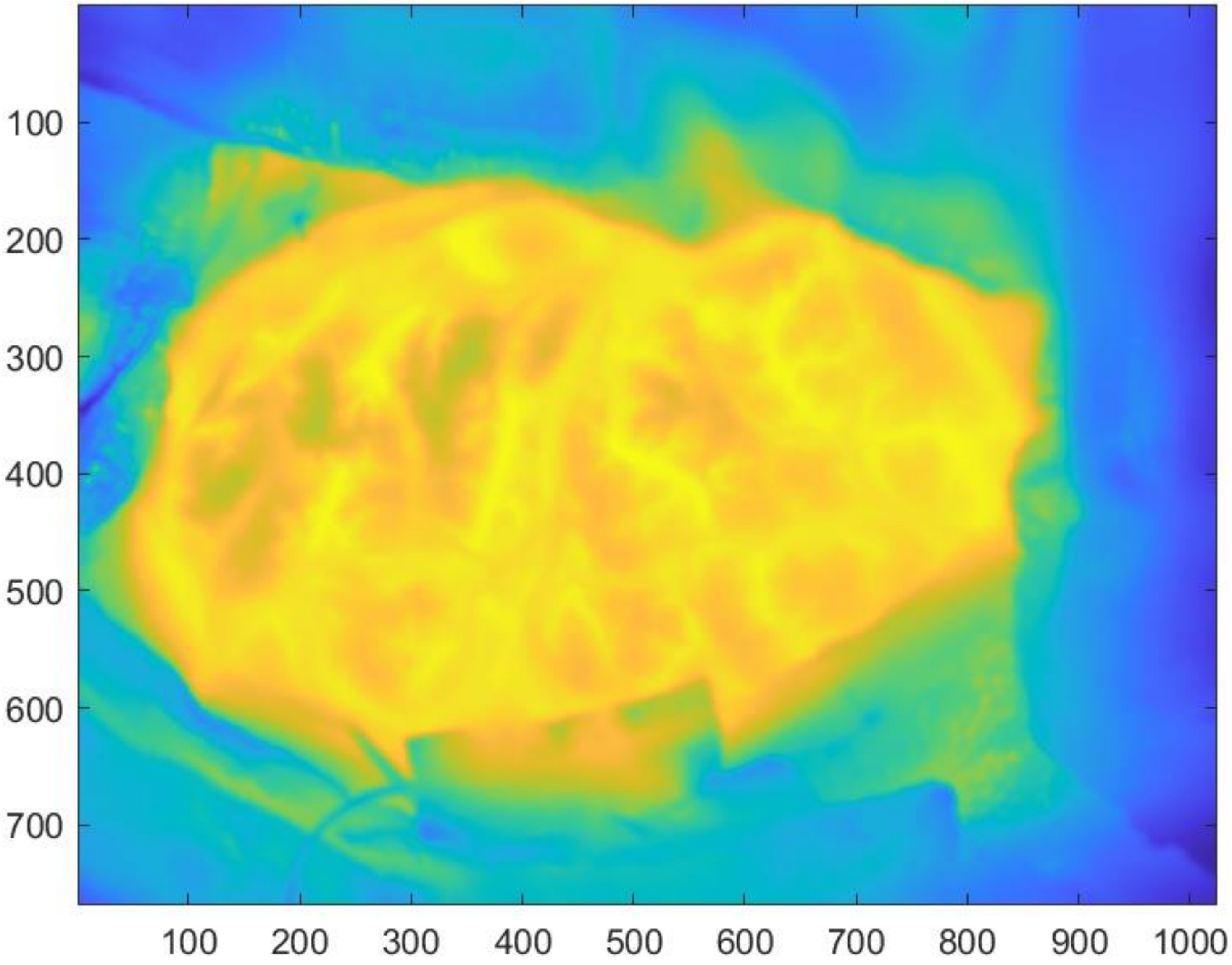
100
200
300
400
500
600
700
100
200
300
400
500
600
700
800
900
1000

P1 (Lip Pursing) DES Key

A. Teeth, Sensory (right)
B. Lips (right) tingling
C. Teeth (right)
D. Teeth, tongue (right)
E. Lips (right)
F. Speech arrest
G. Speech arrest
H. Anomia
I. Anomia

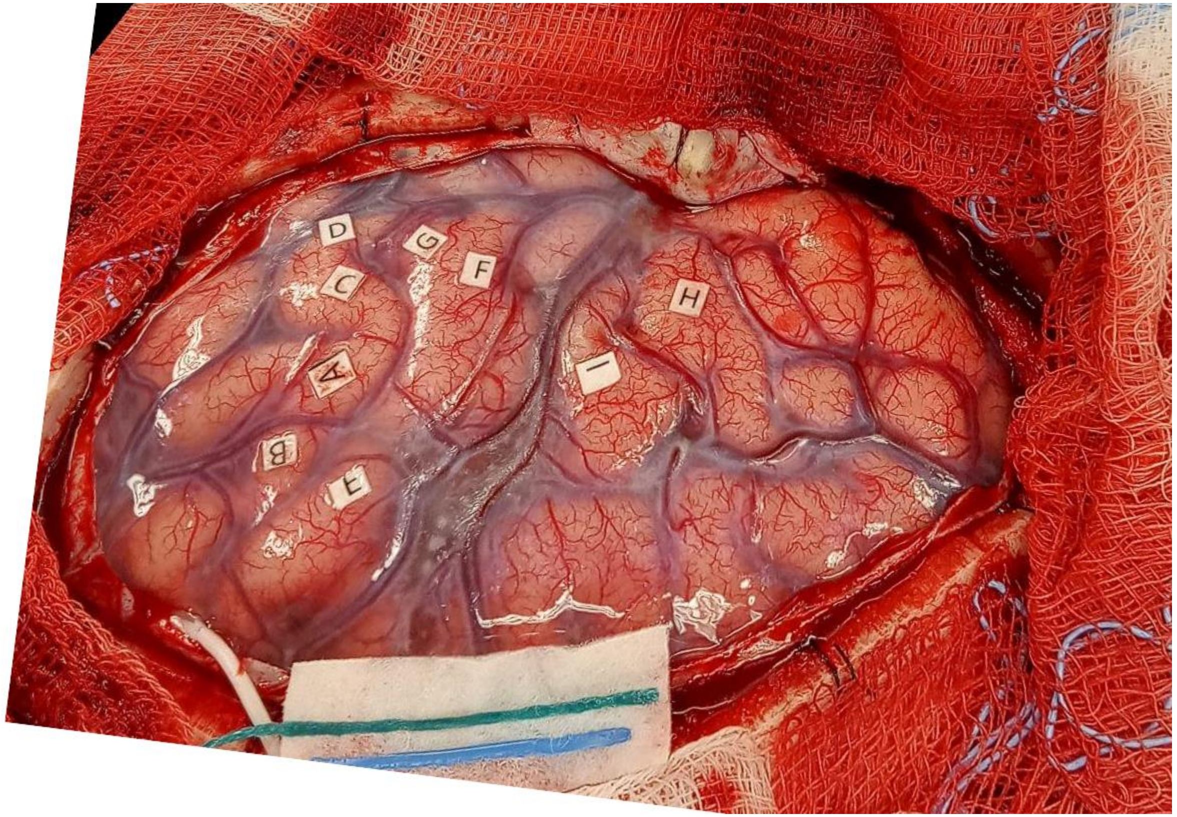

P1 (Lip Pursing) Group Component 1

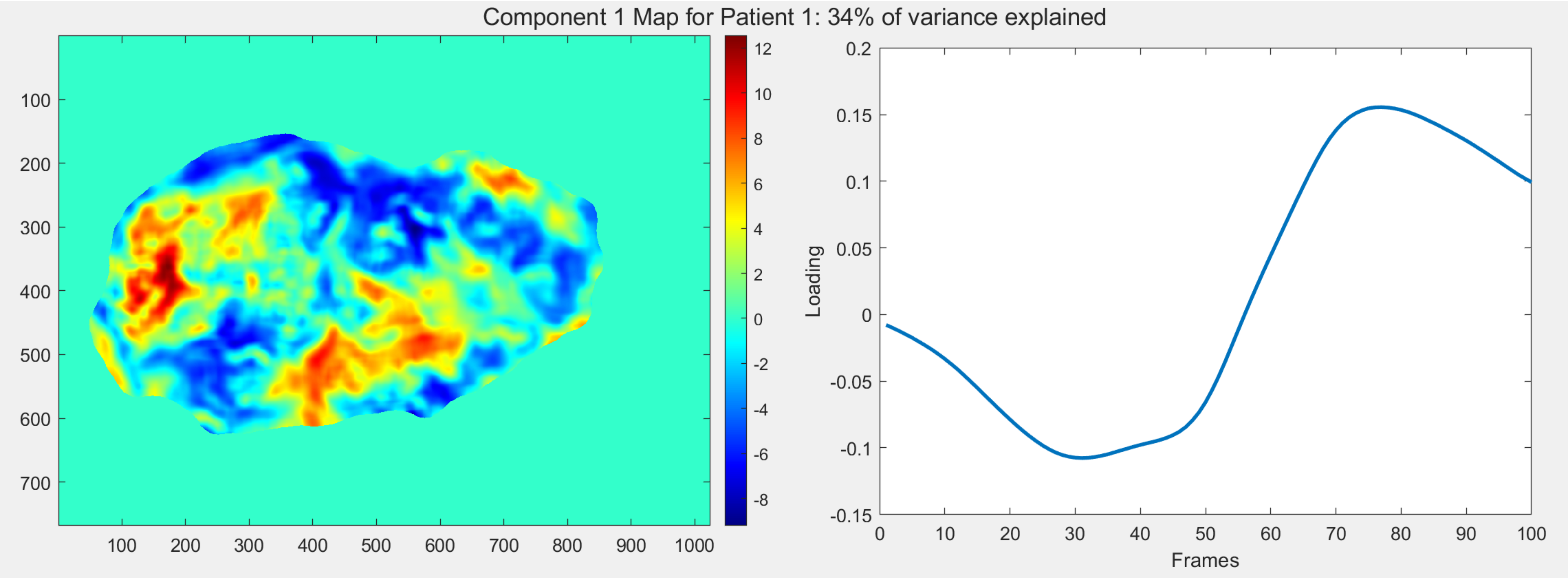

P1 (Lip Pursing) Group Component 2

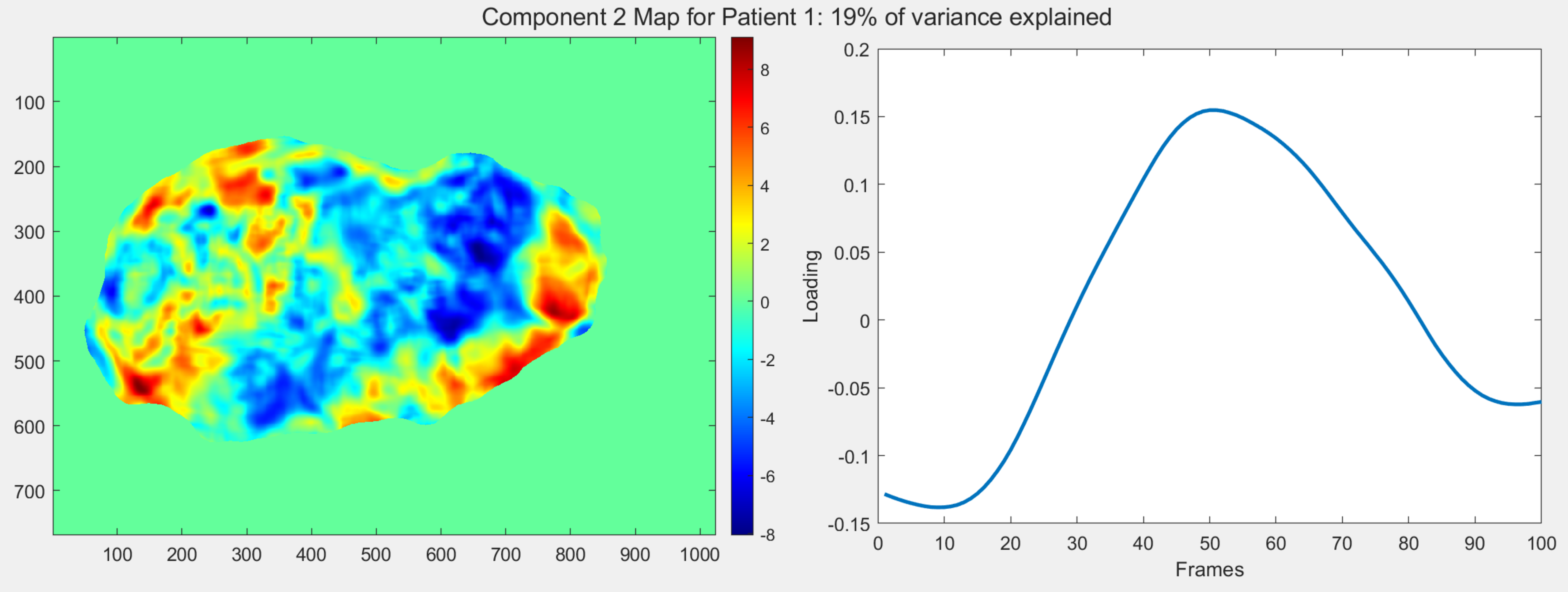

P1 (Lip Pursing) Group Component 3

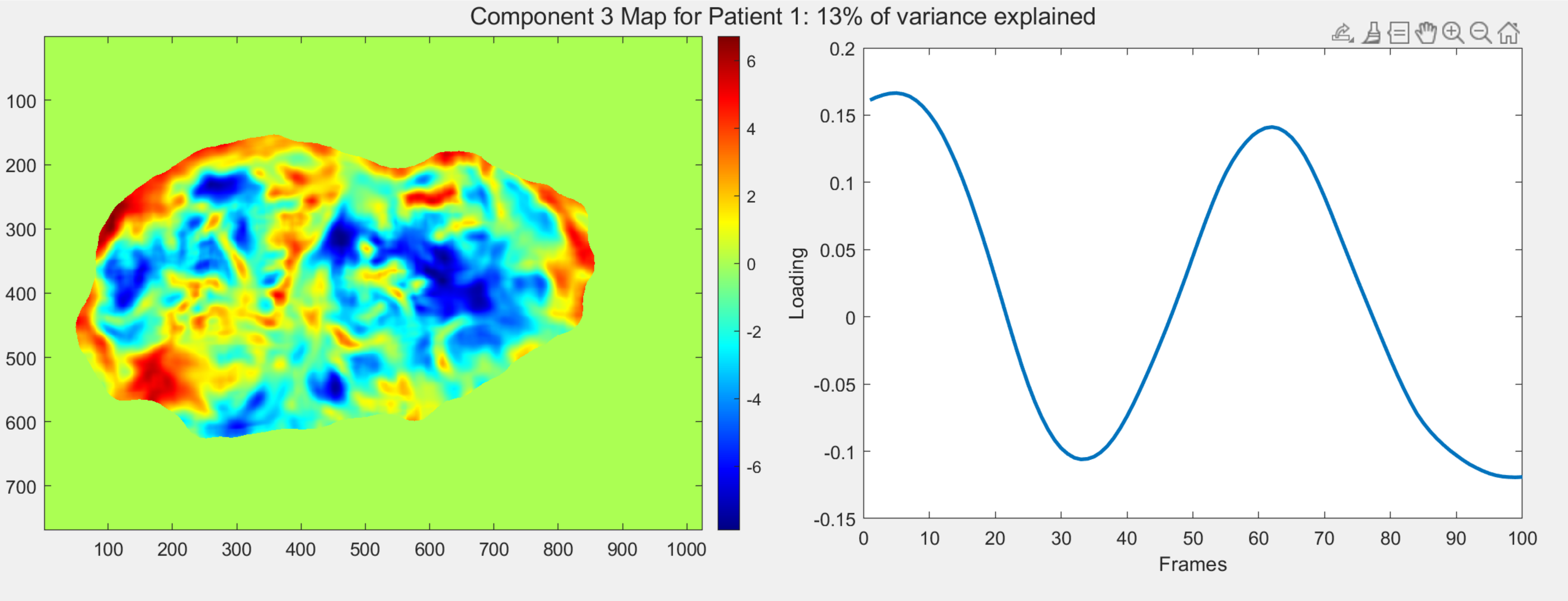

Patient 2: Lip Pursing

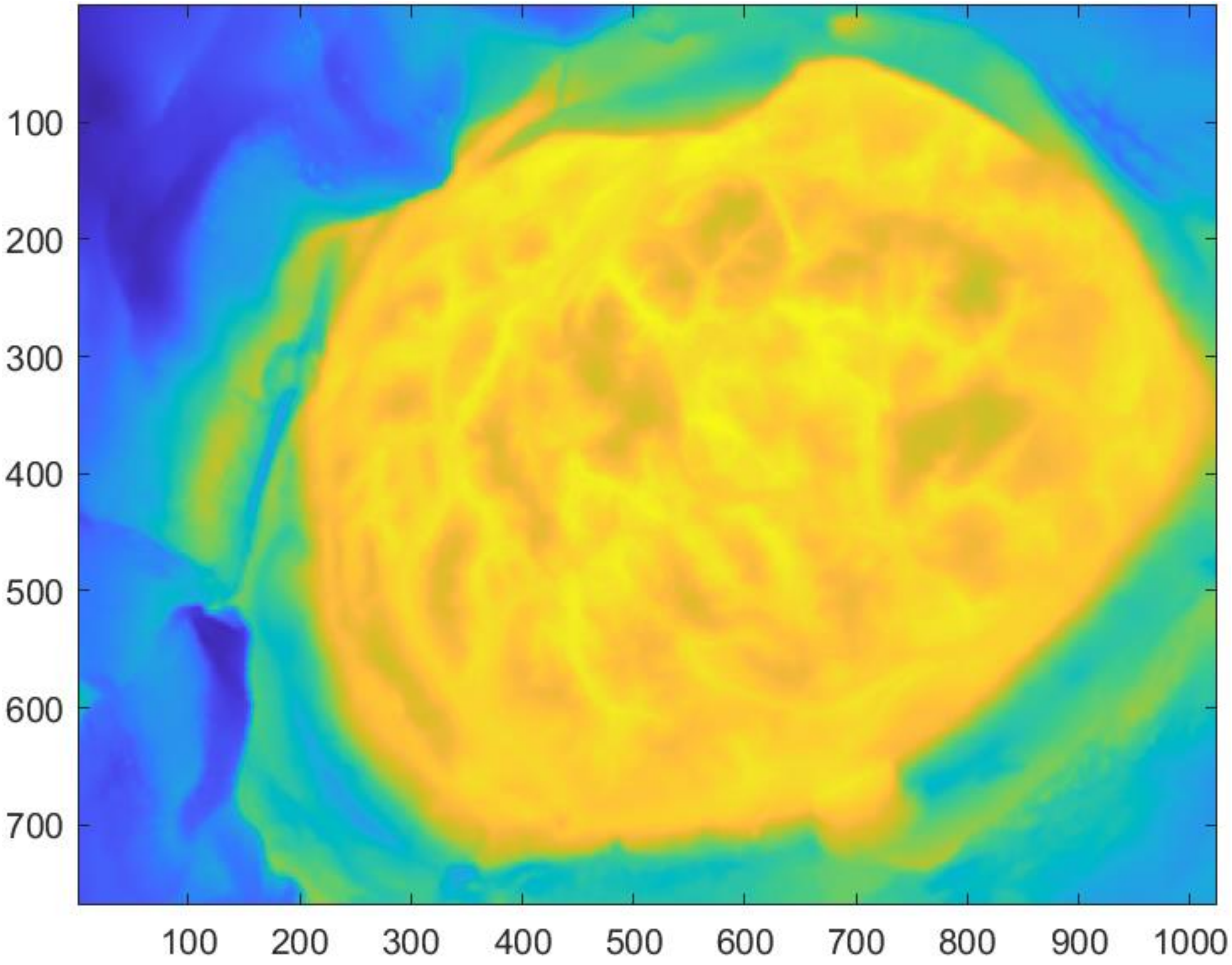
100
200
300
400
500
600
700
100
200
300
400
500
600
700
800
900
1000

P2 (Lip Pursing) DES Key

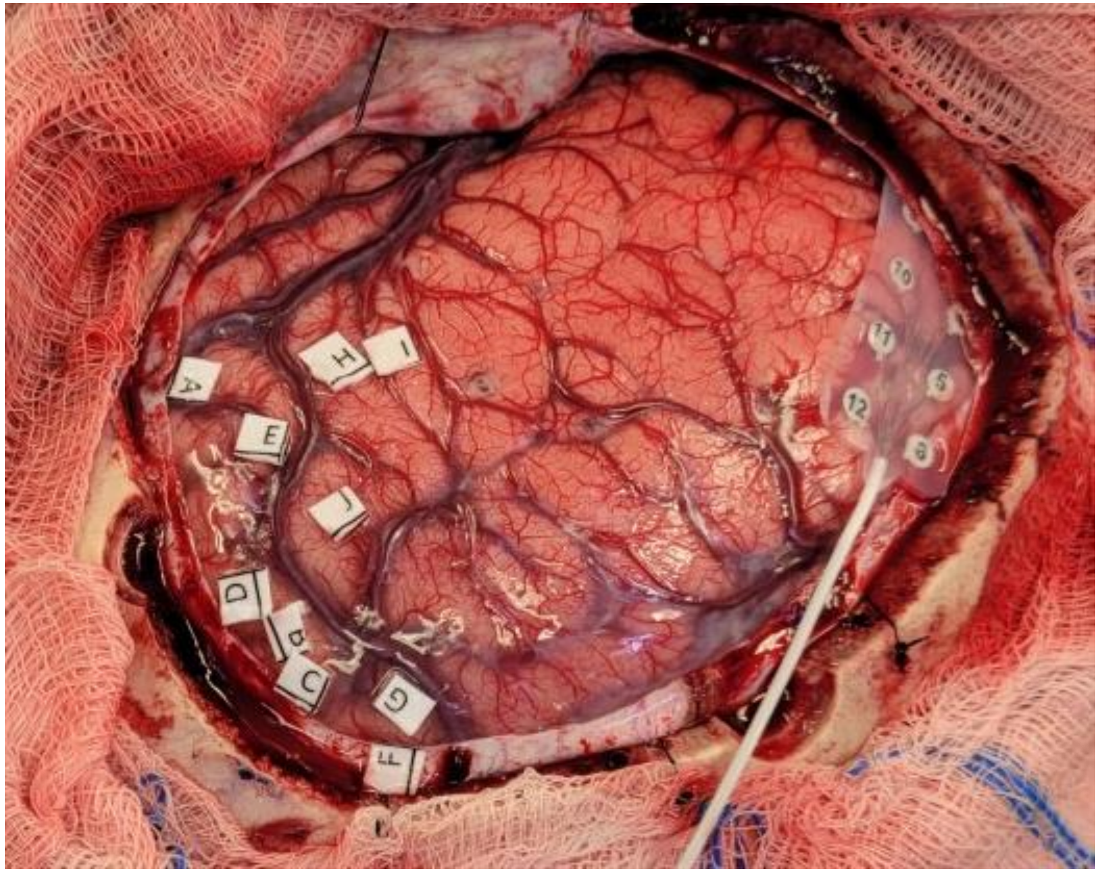

A – Tongue tingling all over
B – Thumb sensory
C – Right hand sensory (3 & 4)
D – Thumb tingling
E – Tongue tingling & movement
F – Wrist flexion
G – Thumb movement
H – Hesitation
I – Hesitation
J – Dysarthria

P2 (Lip Pursing) Group Component 1

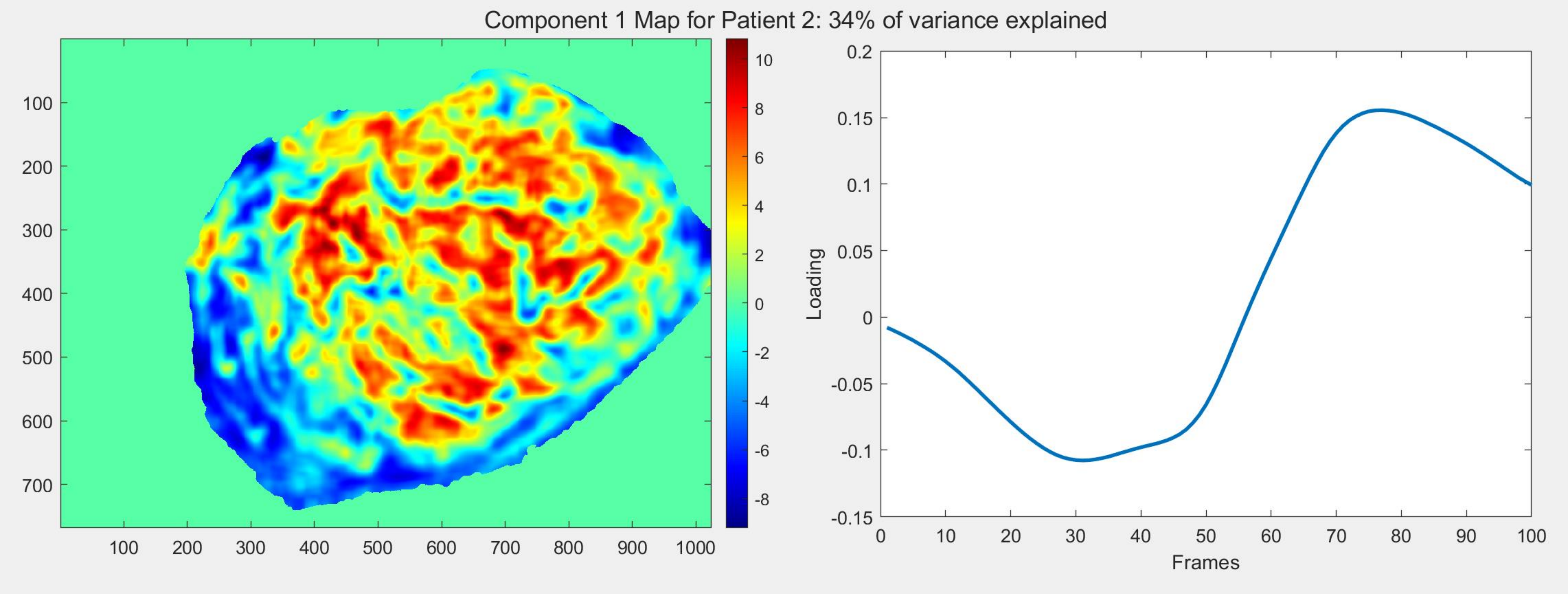

P2 (Lip Pursing) Group Component 2

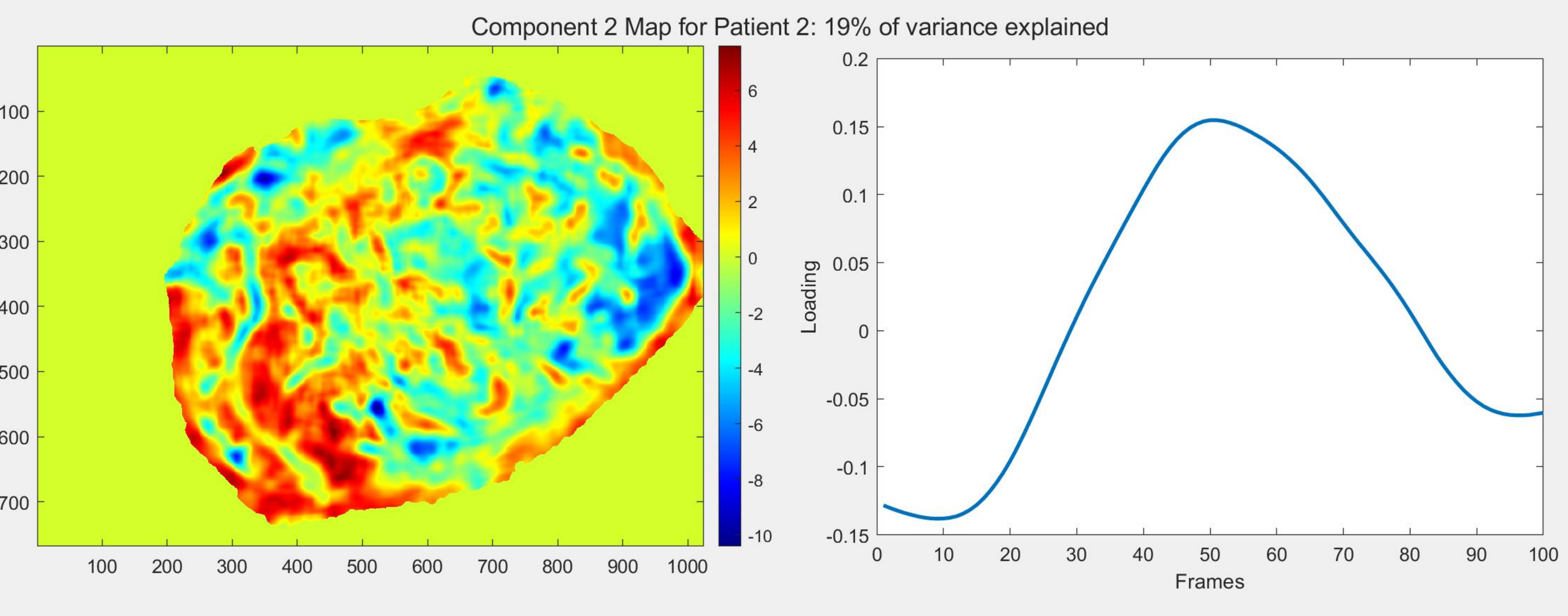

P2 (Lip Pursing) Group Component 3

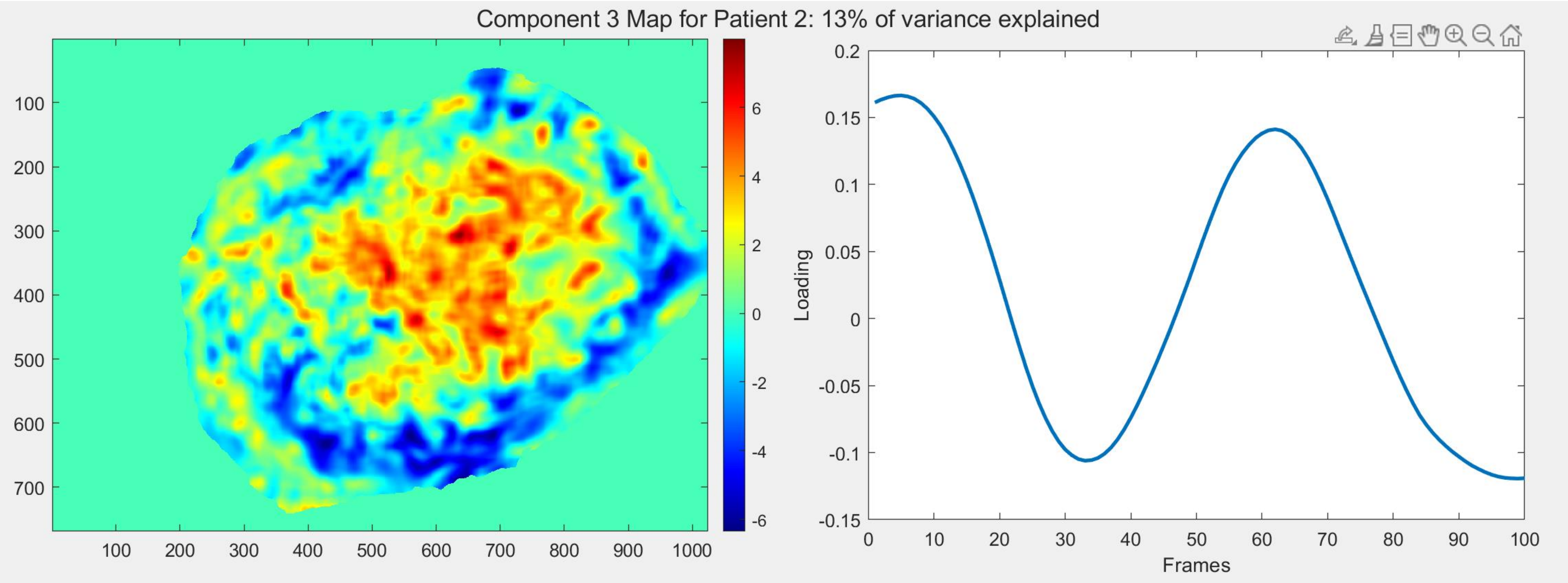

Patient 3: Hand Motor

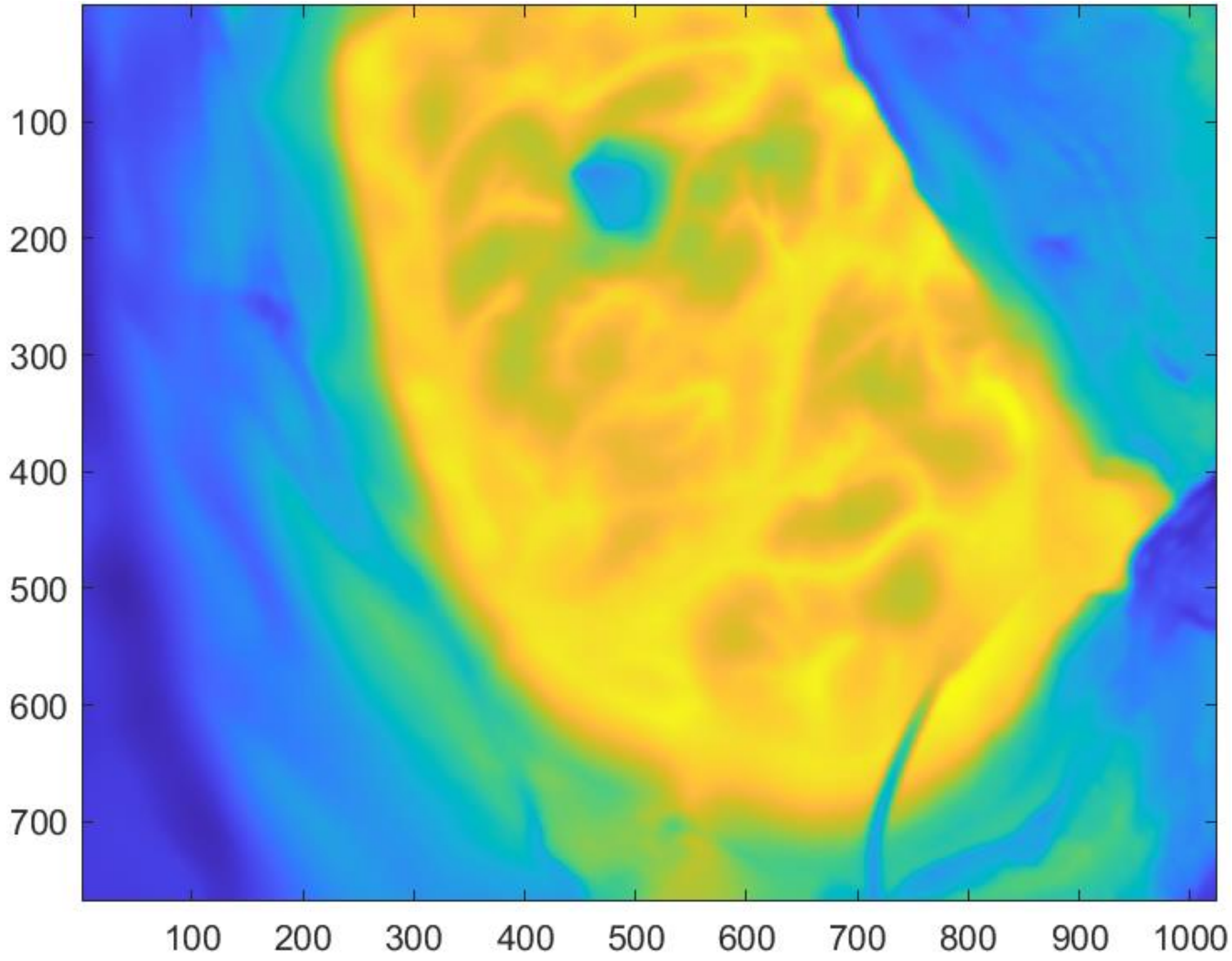

100
200
300
400
500
600
700
100
200
300
400
500
600
700
800
900
1000

P3 (Hand Motor) DES Key

- A – Middle finger tingling
- B – 2nd finger
- C – Thumb
- D – Left pinky / sensory
- **E – Left arm + hand motor**
- F – Left wrist
- 1 – wrist
- 2 – jaw sensation
- 3 – mouth motor
- 4 – lateral mouth motor

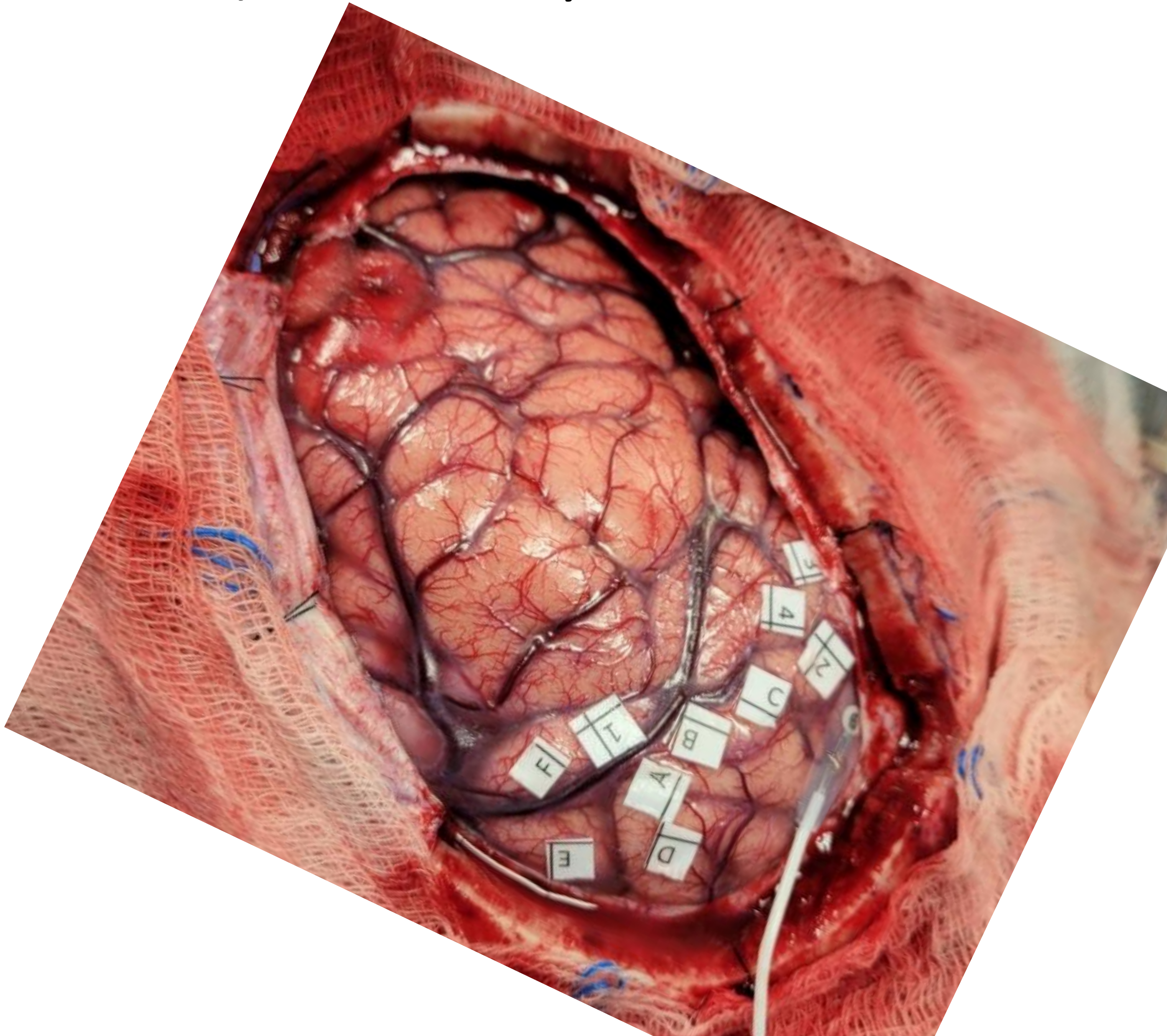

P3 (Hand Motor) Group Component 1

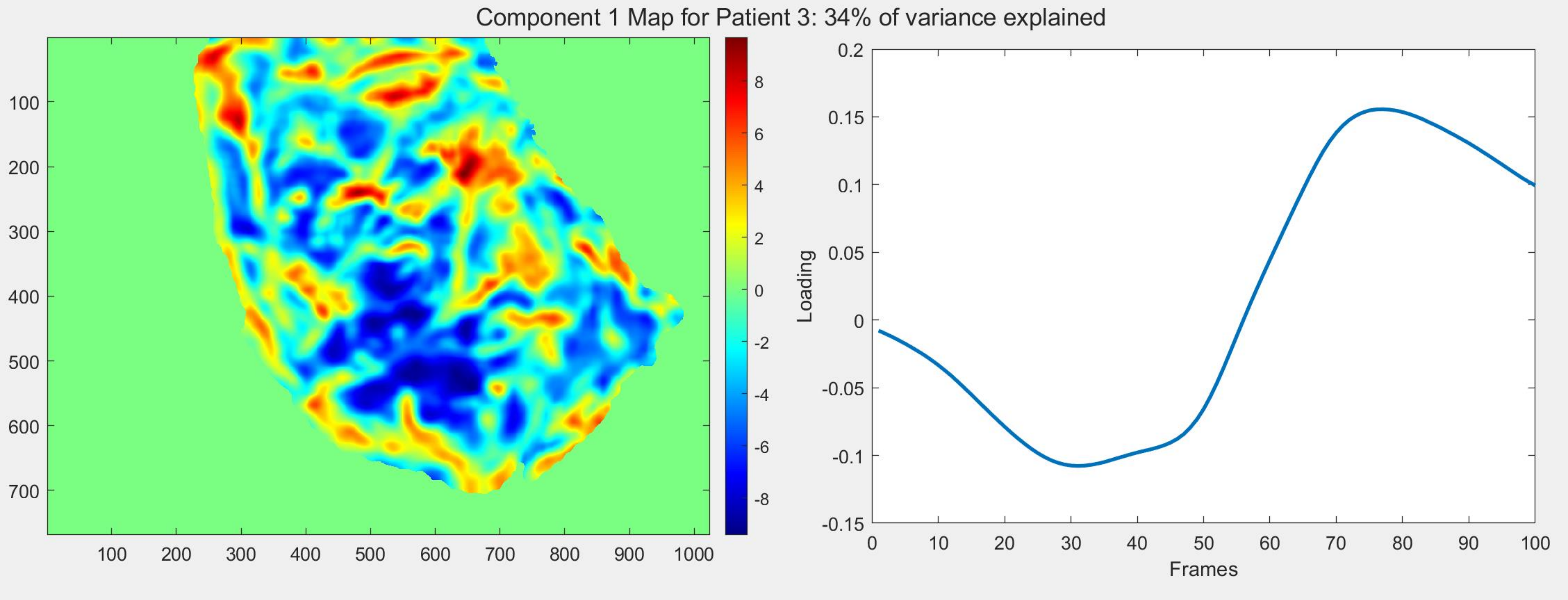

P3 (Hand Motor) Group Component 2

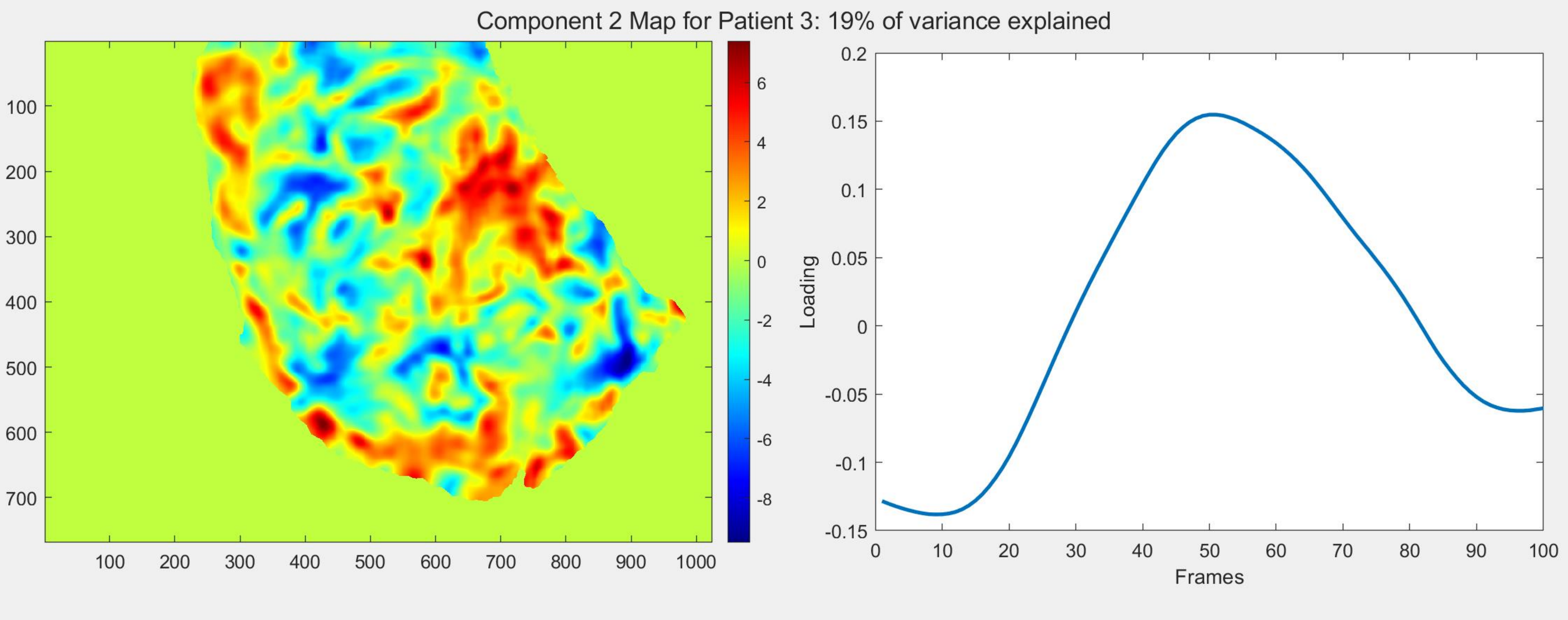

P3 (Hand Motor) Group Component 3

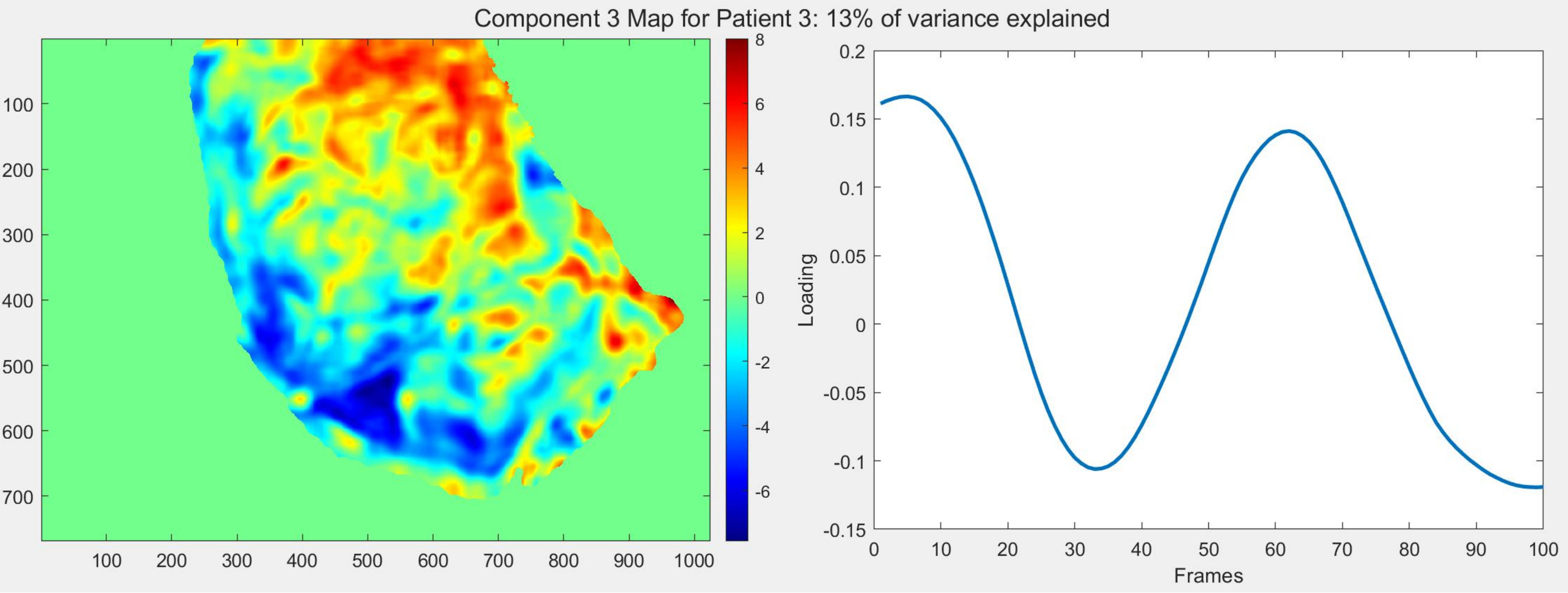

Patient 4: Hand Motor

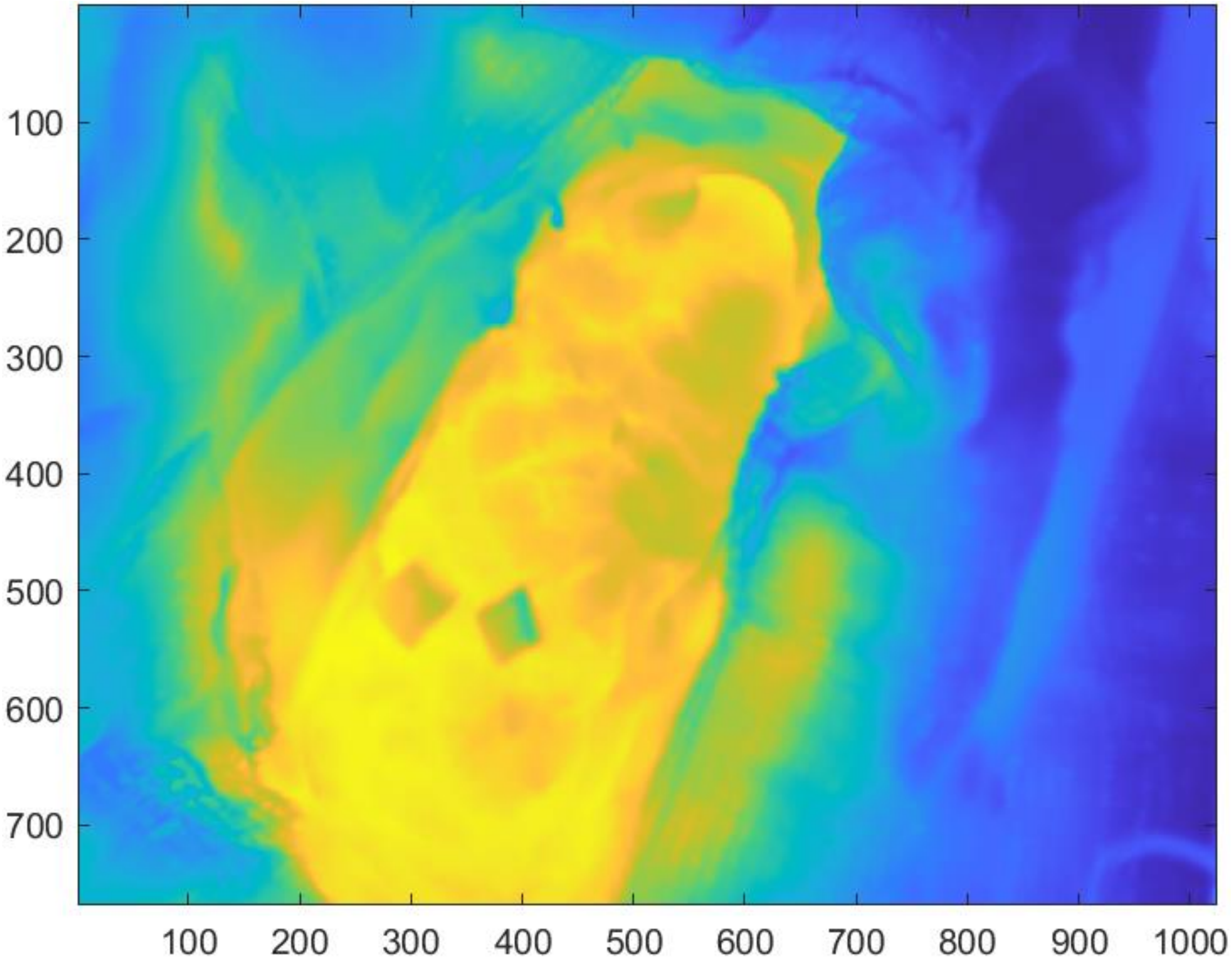
100
200
300
400
500
600
700
100
200
300
400
500
600
700
800
900
1000

P4 (Hand Motor) DES Key

- A – Finger Adduction
- B – Finger Extension
- C – Finger Sensory
- D – Finger Sensory
- E – Finger Sensory
- G – Thumb Sensory
- H – Wrist Motor
- I – Forearm Motor
- J – Hand Motor

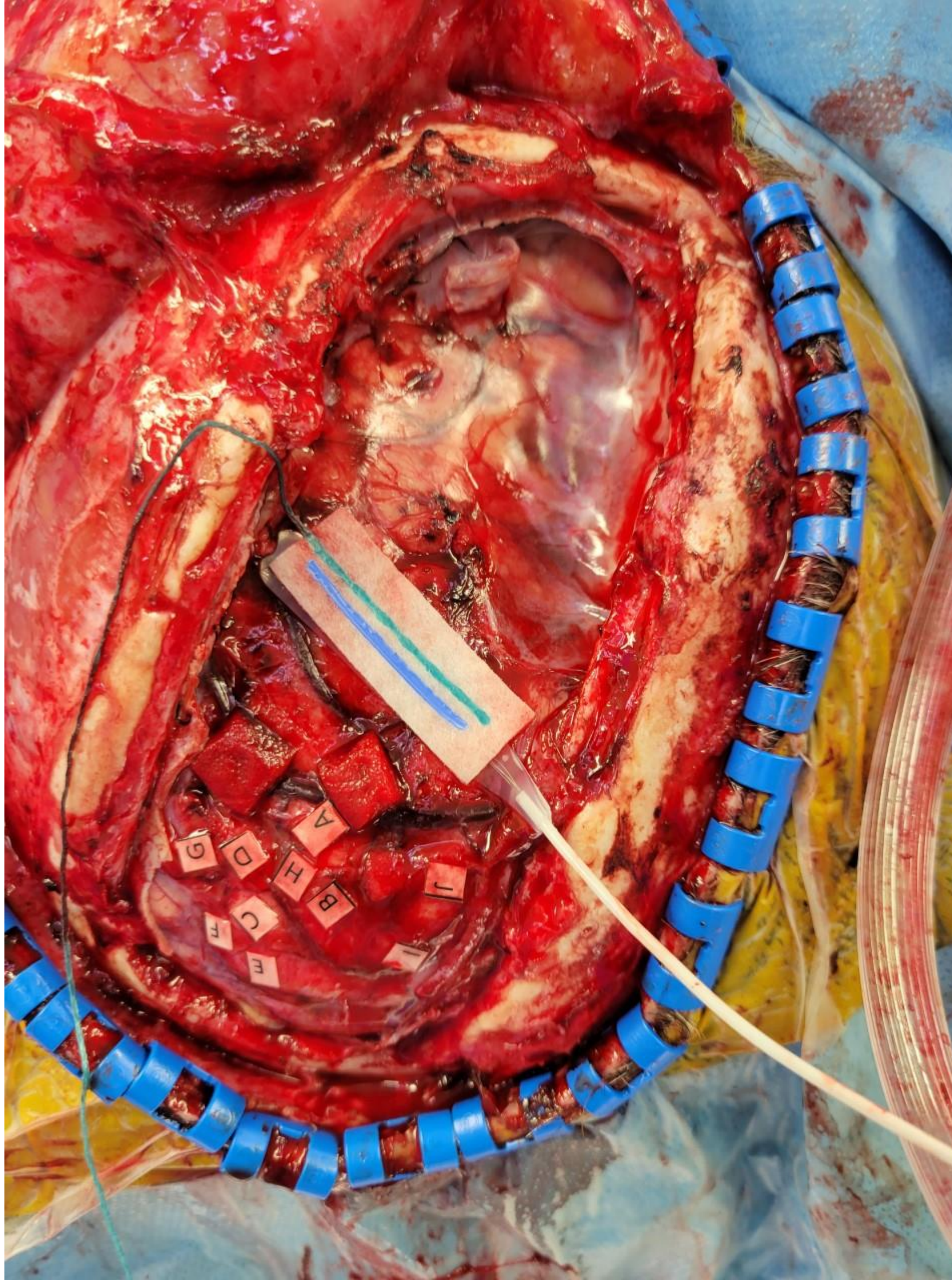

P4 (Hand Motor) Group Component 1

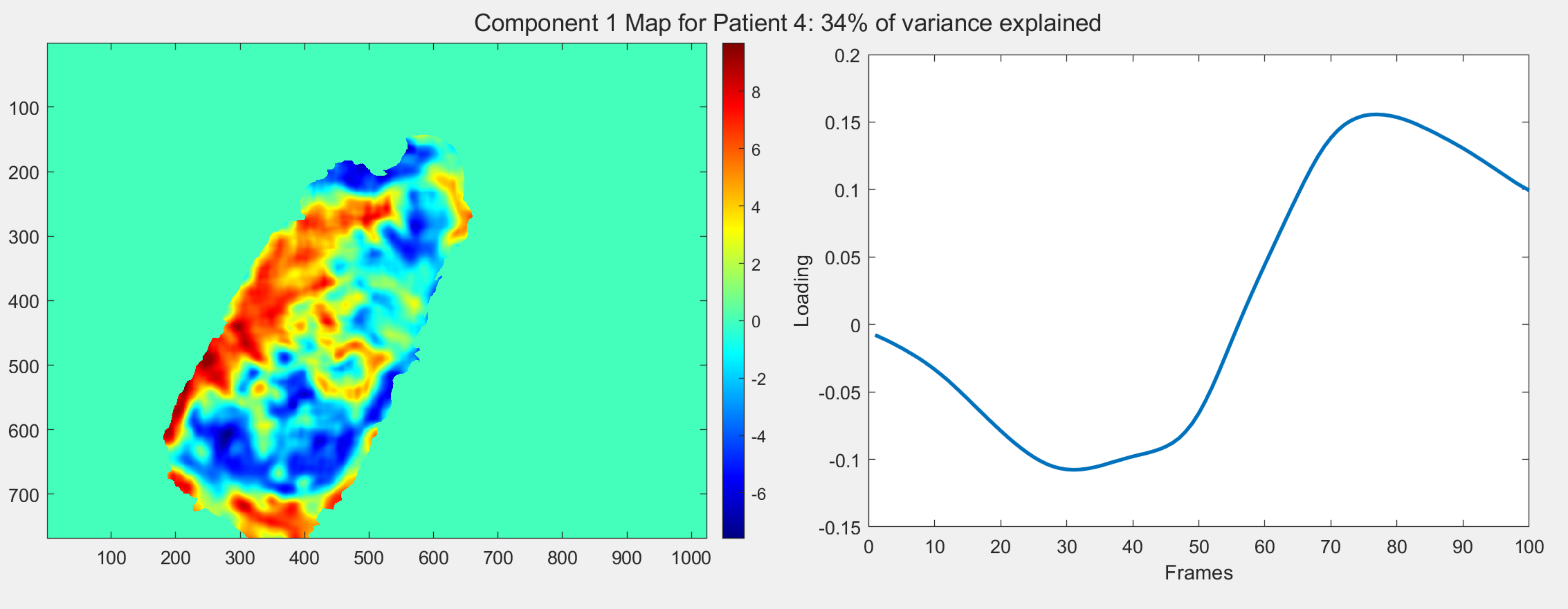

P4 (Hand Motor) Group Component 2

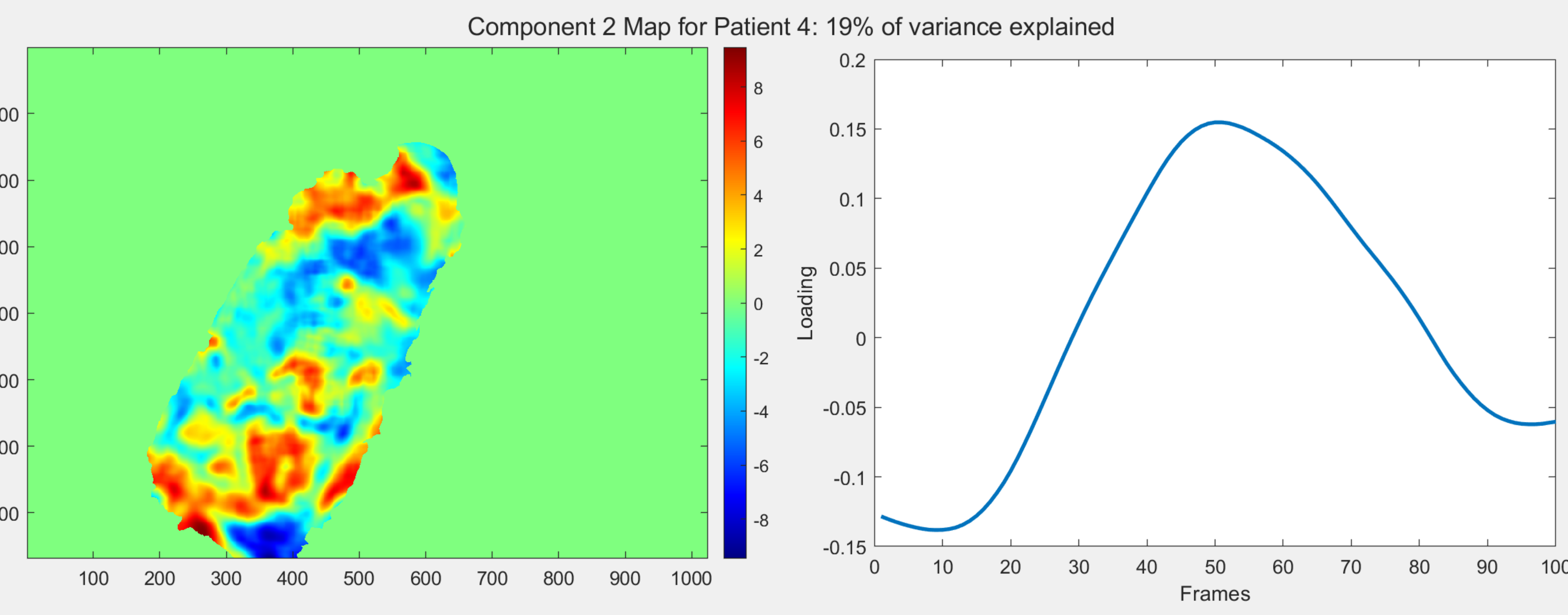

P4 (Hand Motor) Group Component 3

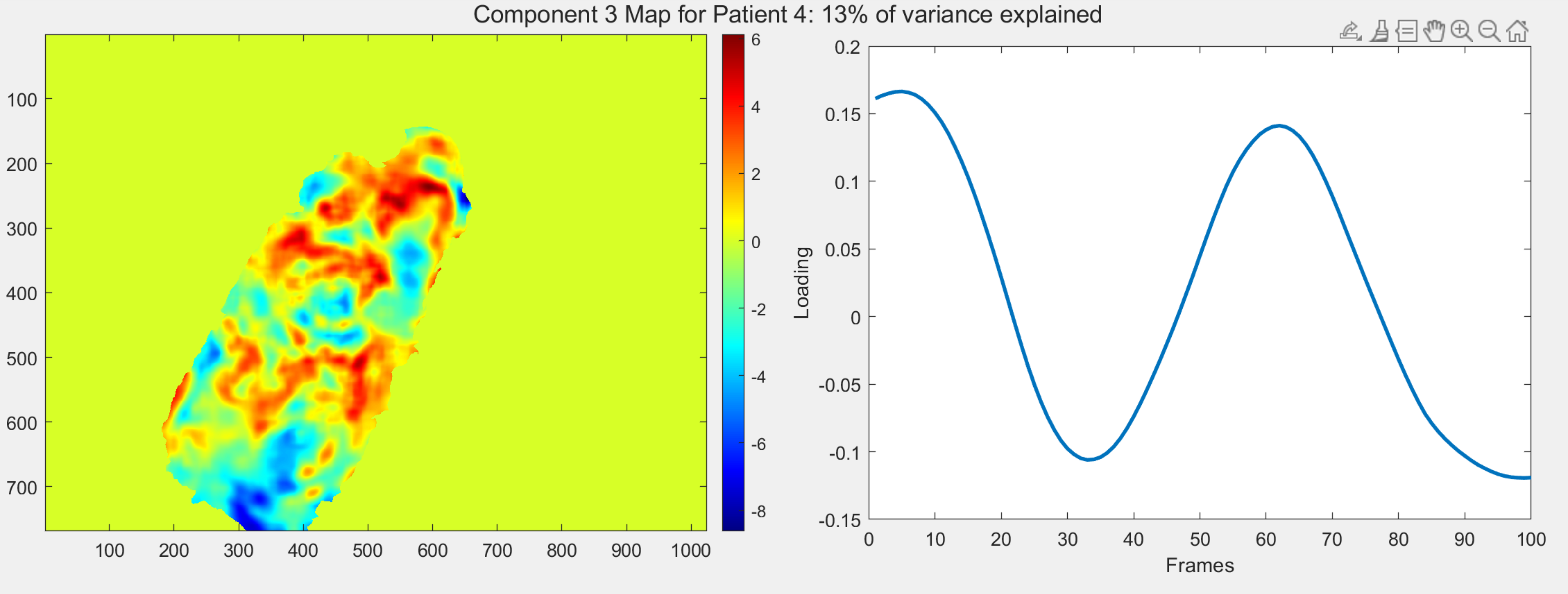

Patient 5: Hand Sensory

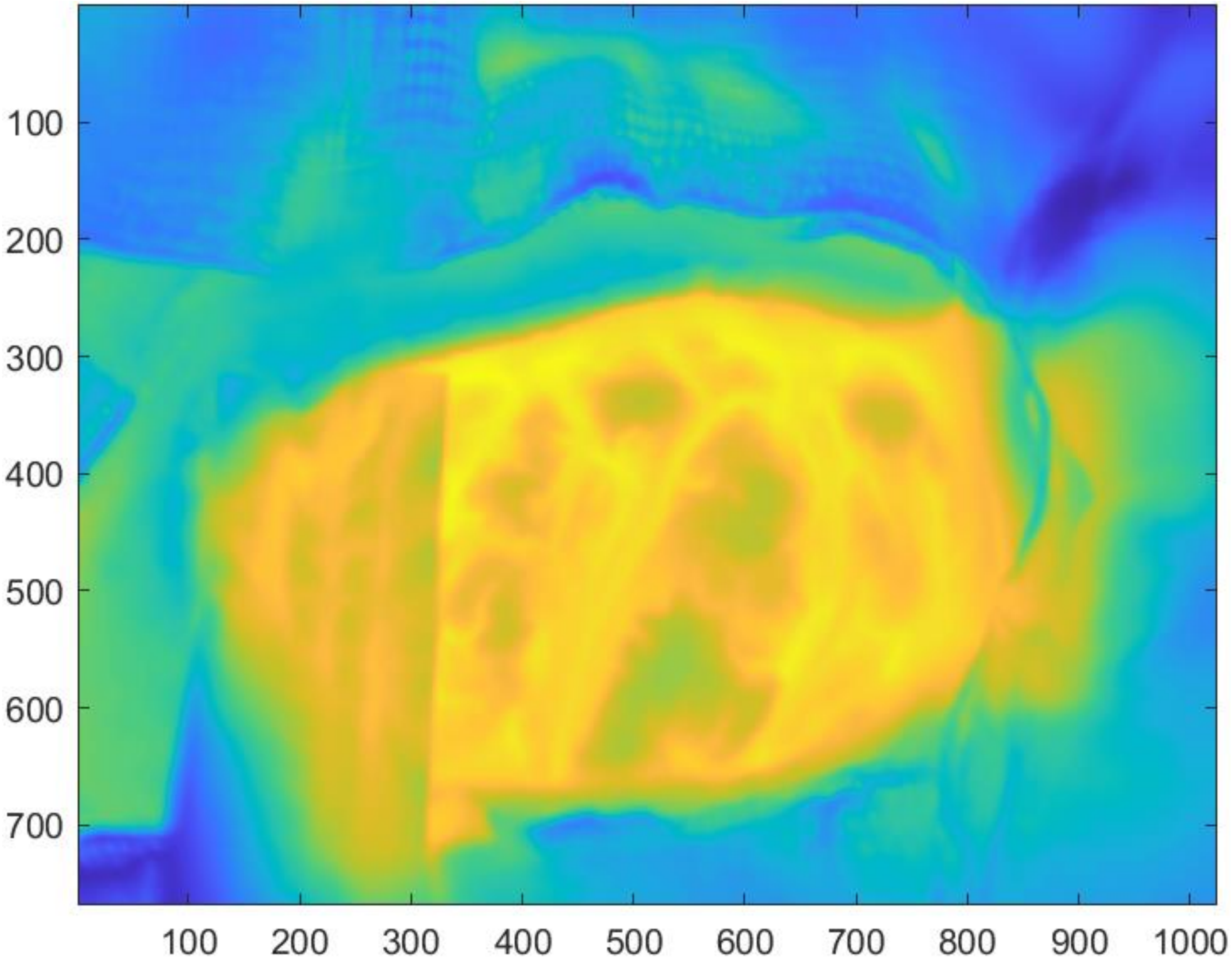
100
200
300
400
500
600
700
100
200
300
400
500
600
700
800
900
1000

1. Forearm Sensory
2. Forearm Tingling
3. Left Palm Tingling
4. 3,4,5 Finger Flexion
5. **Finger Tingling**
6. Left Upper Arm Tingling
7. Torso Tingling (left)
8. Left Upper Side Tingling
9. Left Wrist / Arm Movement
10. Left Wrist Flexion
11. Face Tinging
12. Elbow Flexion
13. Left Shoulder Movement

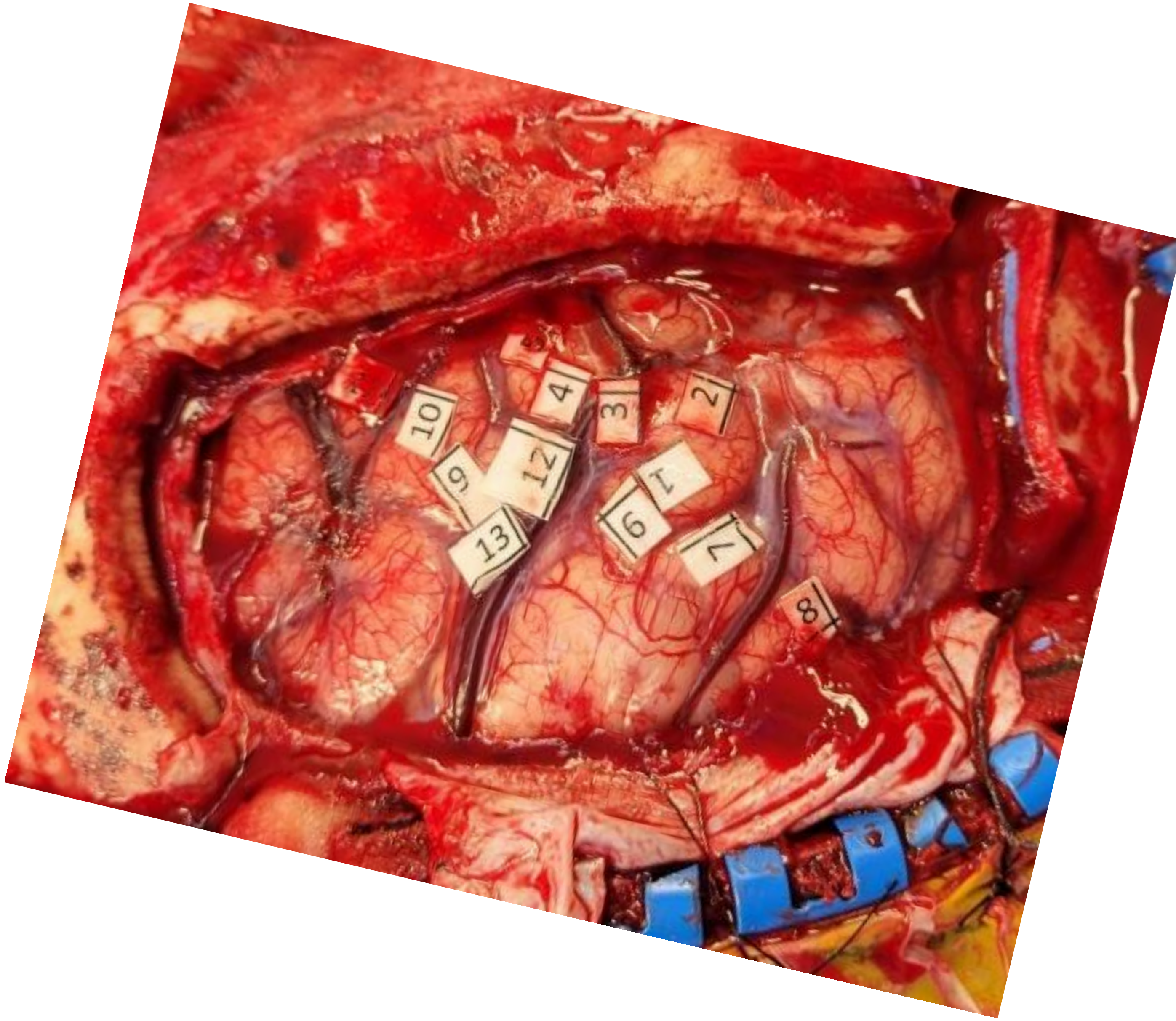

P5 (Hand Sensory) Group Component 1

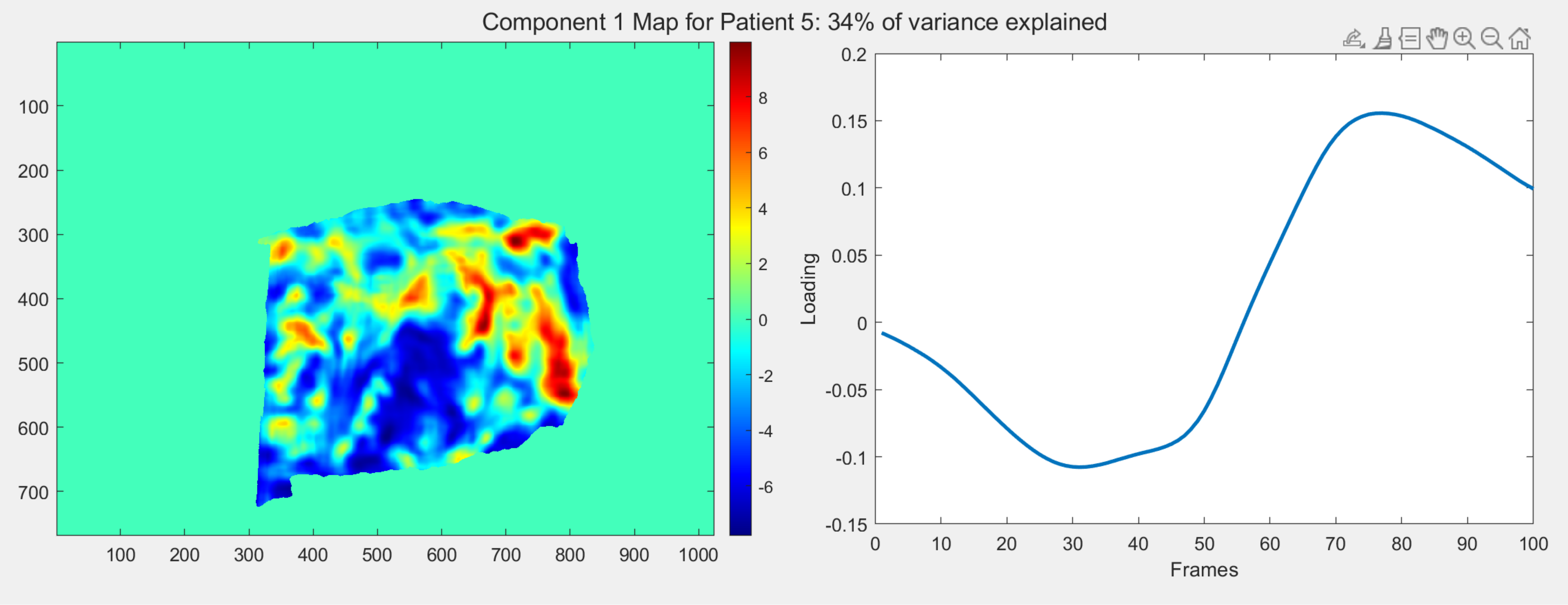

P5 (Hand Sensory) Group Component 2

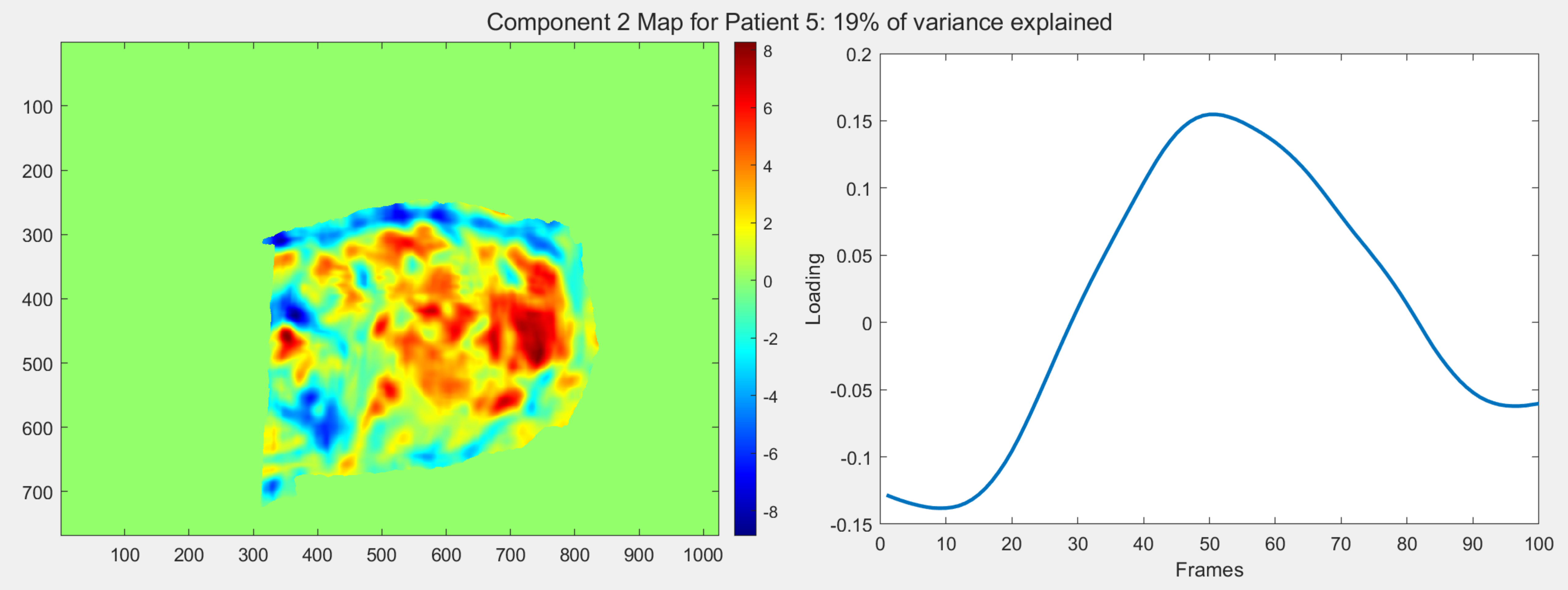

P5 (Hand Sensory) Group Component 3

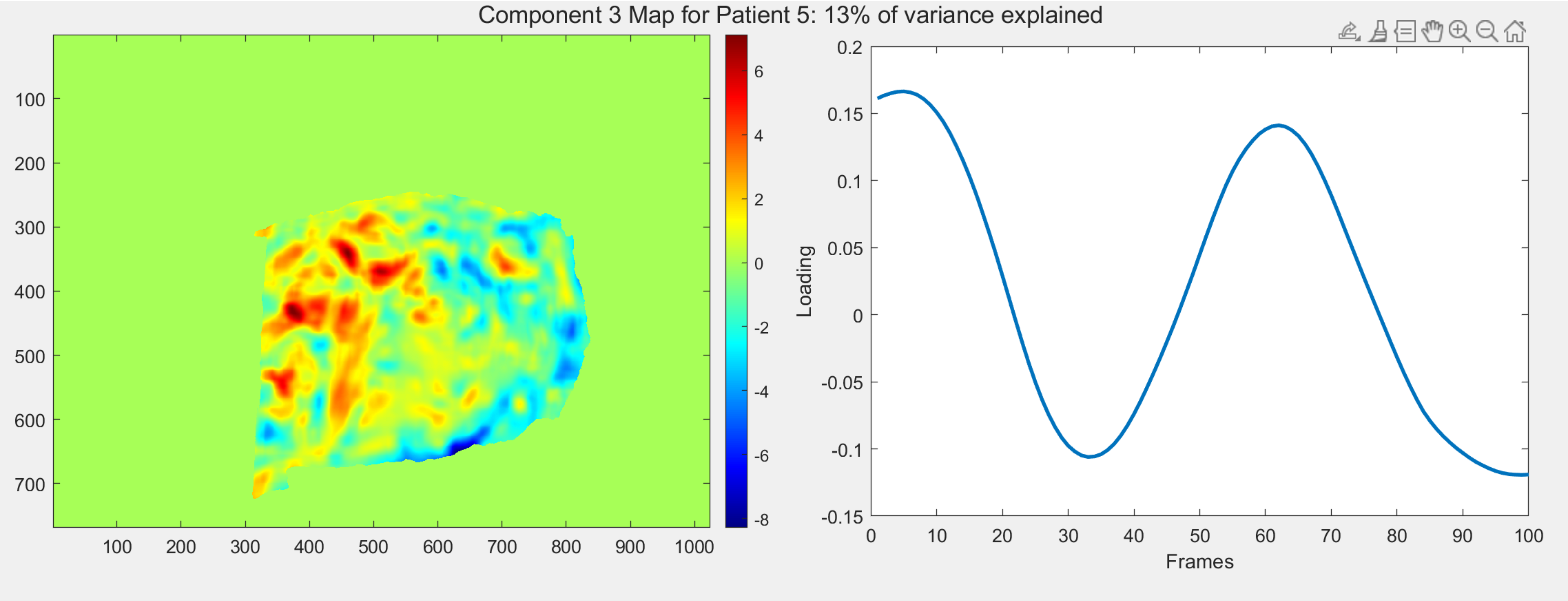

Patient 6: Hand Sensory

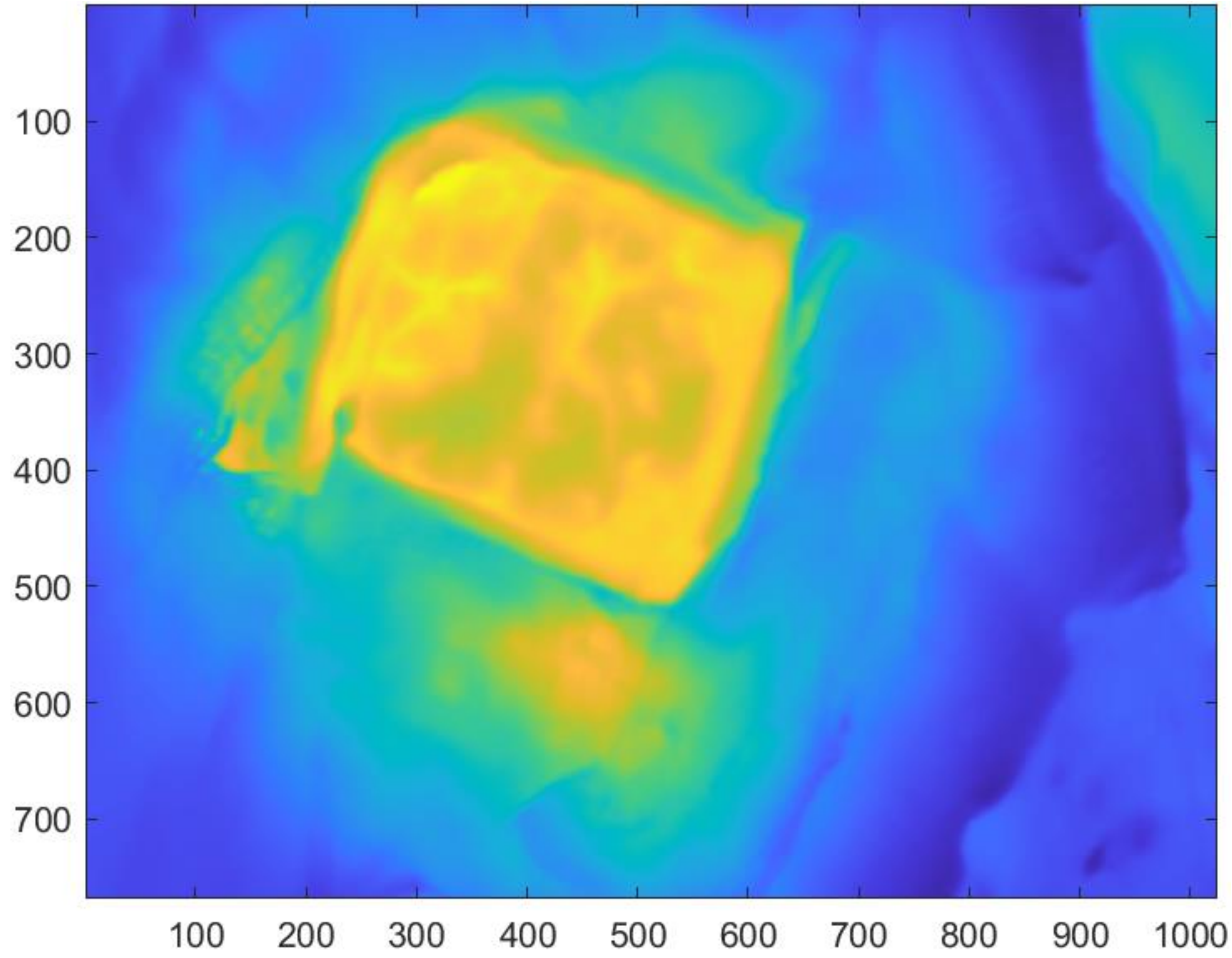
100
200
300
400
500
600
700
100
200
300
400
500
600
700
800
900
1000

P6 (Hand Sensory) DES Key

- A – Left finger extension
- B – Left hand extension
- C – Cheek tingling
- D – Thumb numbness
- E – Pinky Numbness

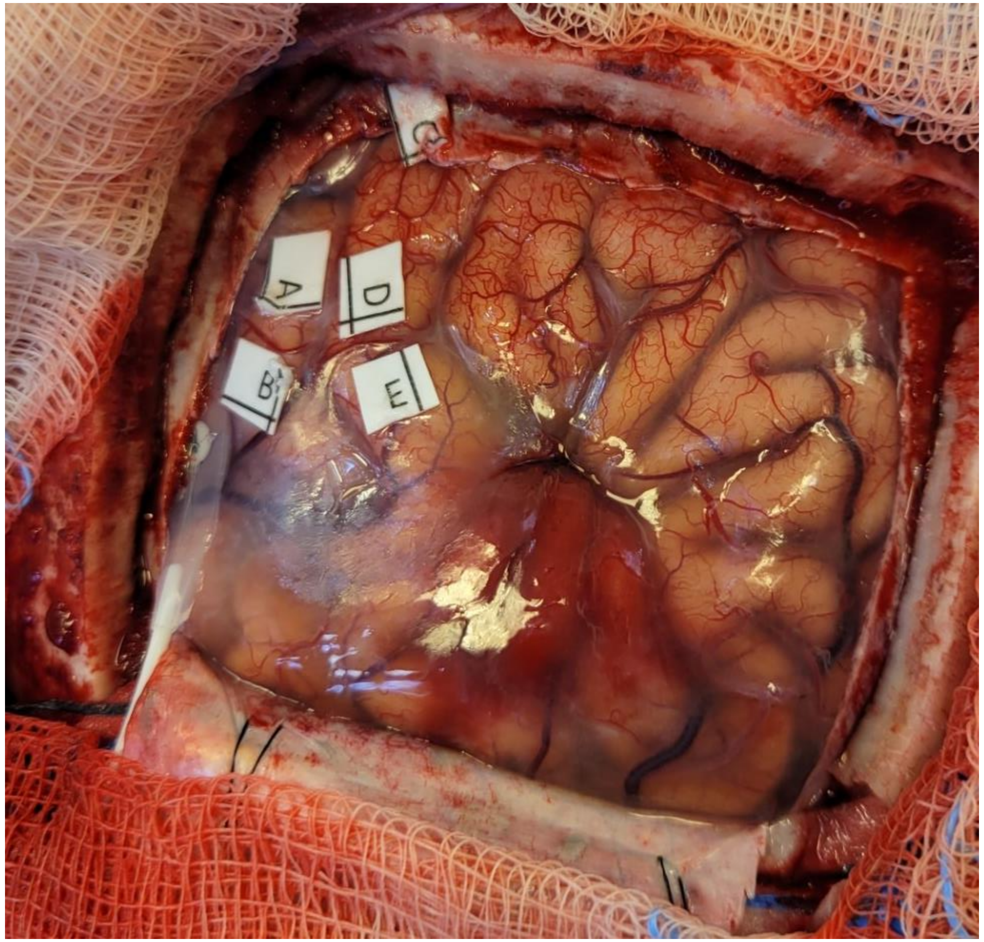

P6 (Hand Sensory) Group Component 1

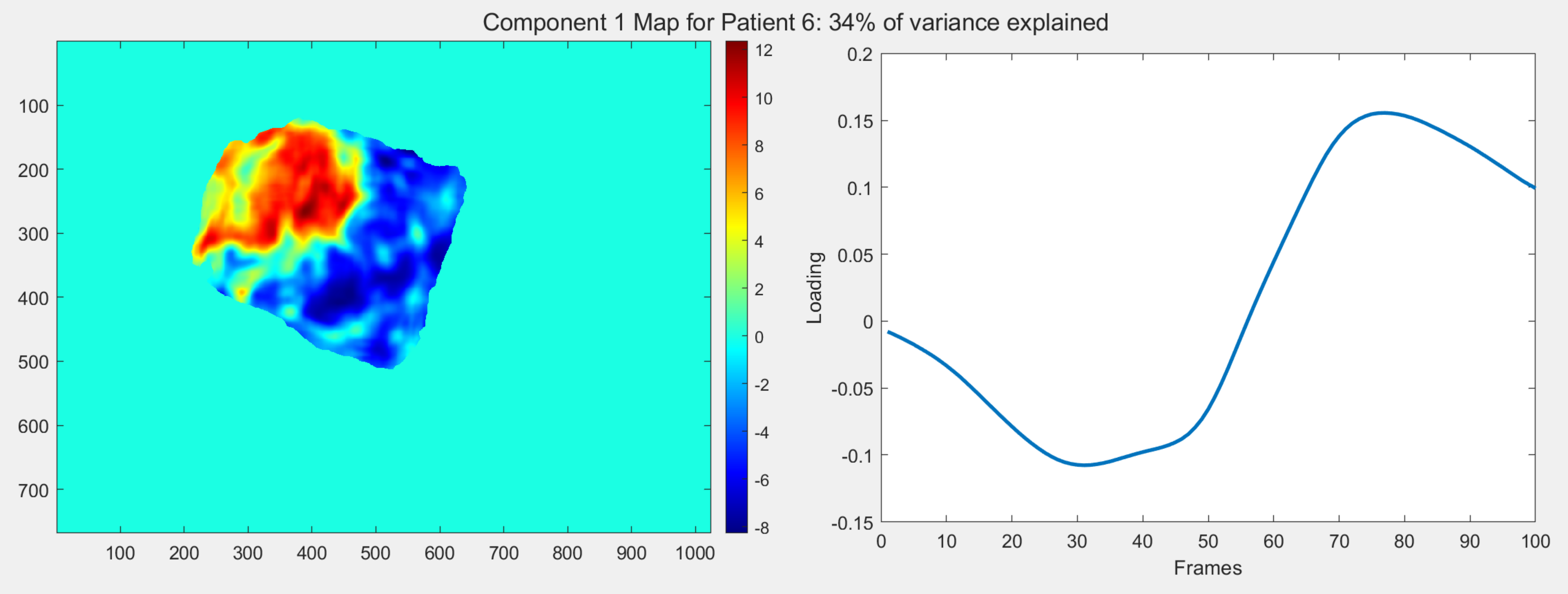

P6 (Hand Sensory) Group Component 2

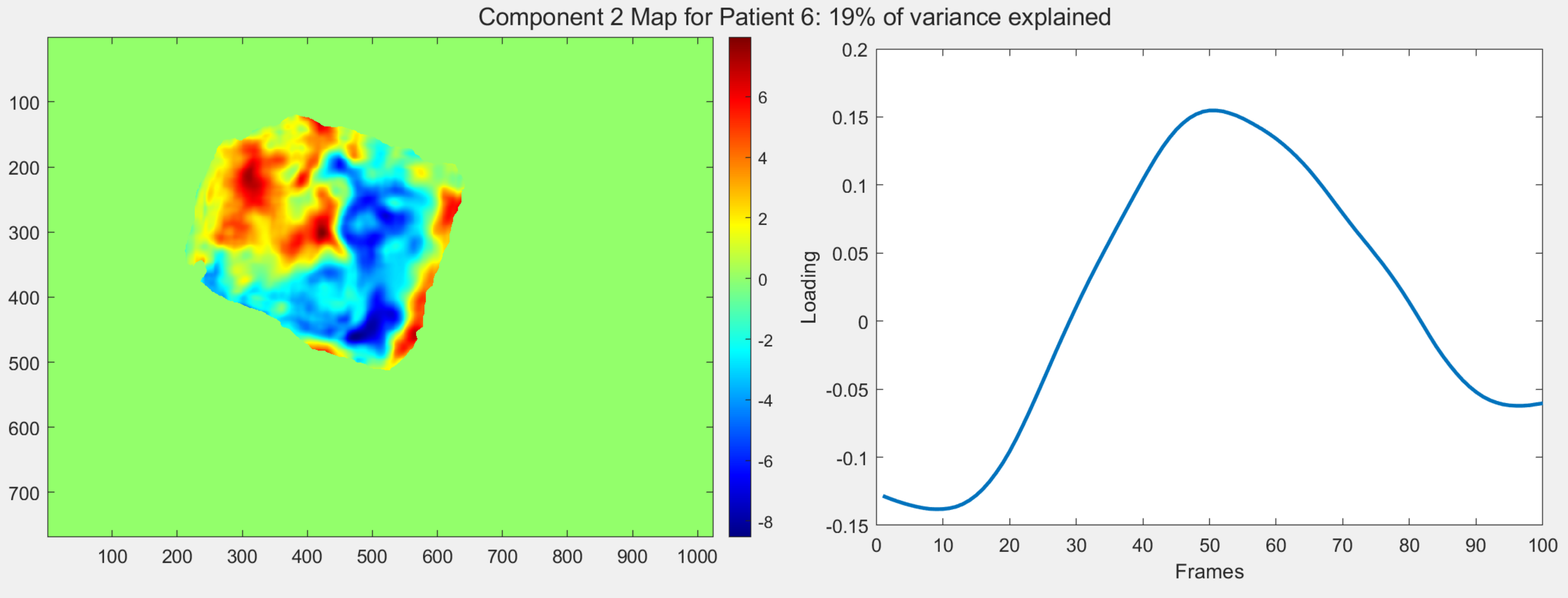

P6 (Hand Sensory) Group Component 3

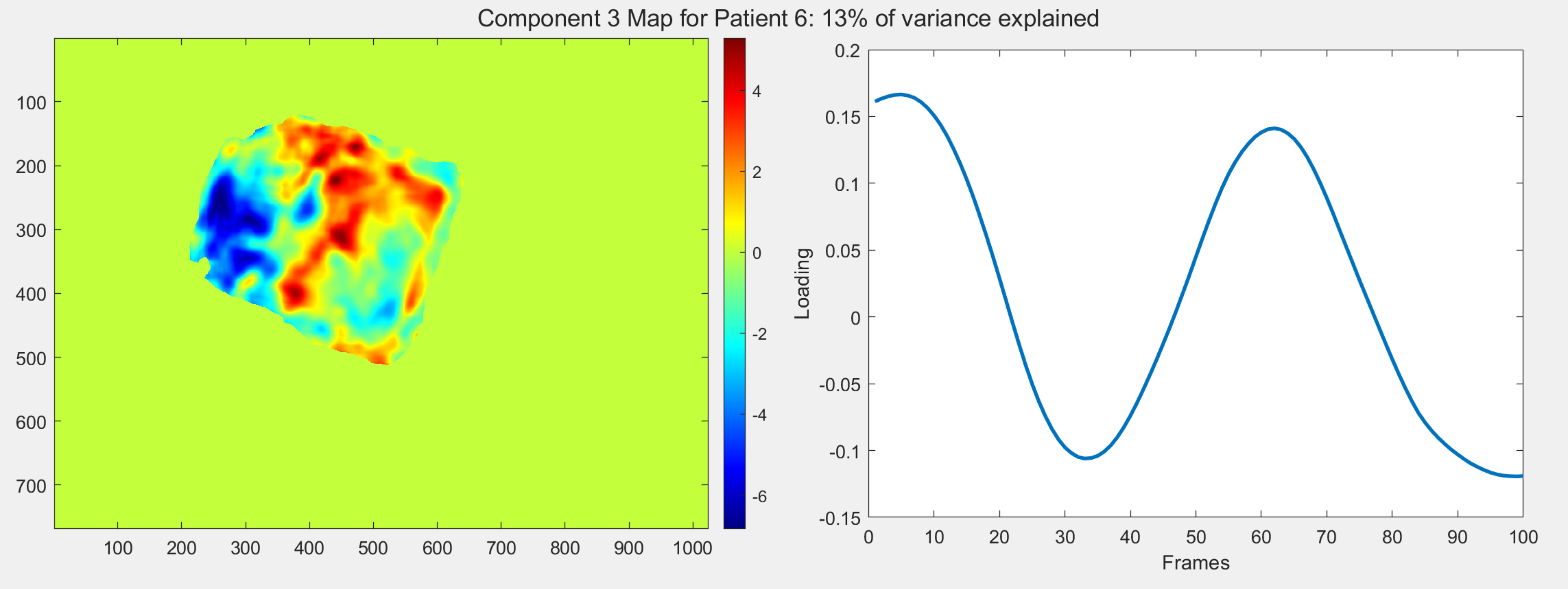

Supplement: Supplementary file 1 [file brainsci-13-01091-s001.zip › brainsci-2480105-supplementary.pdf]
